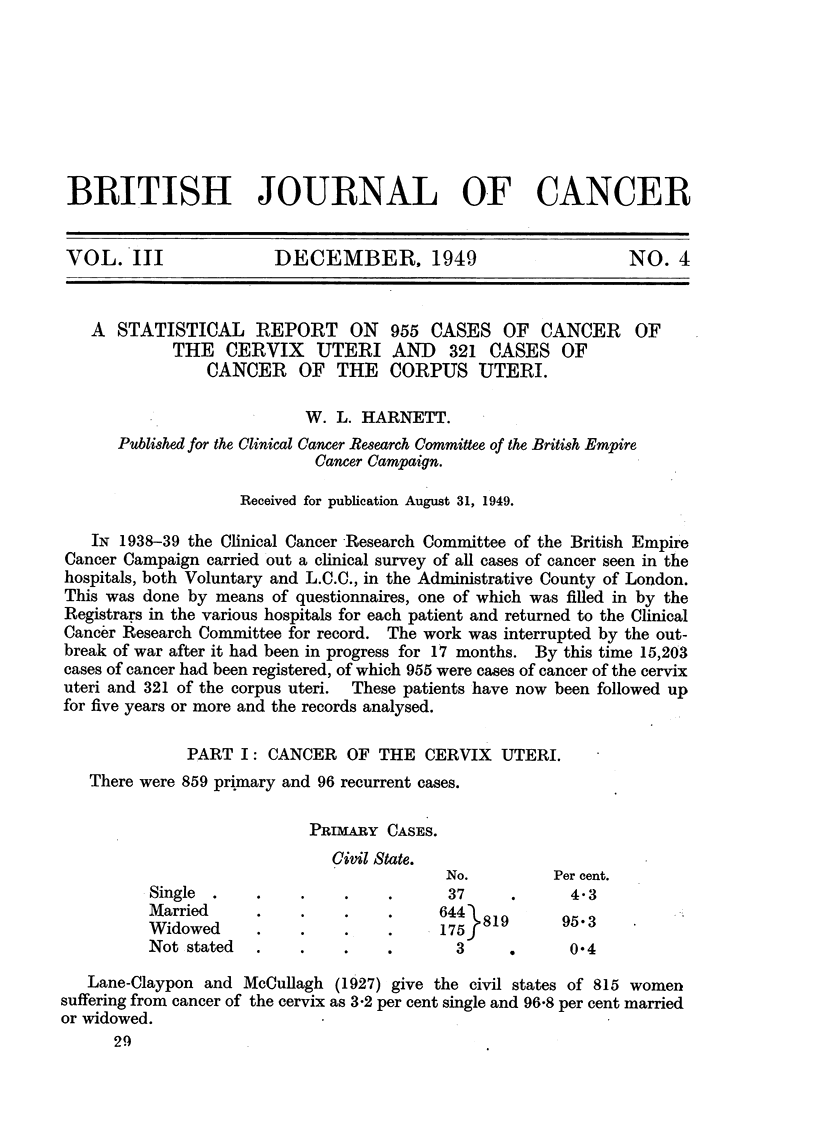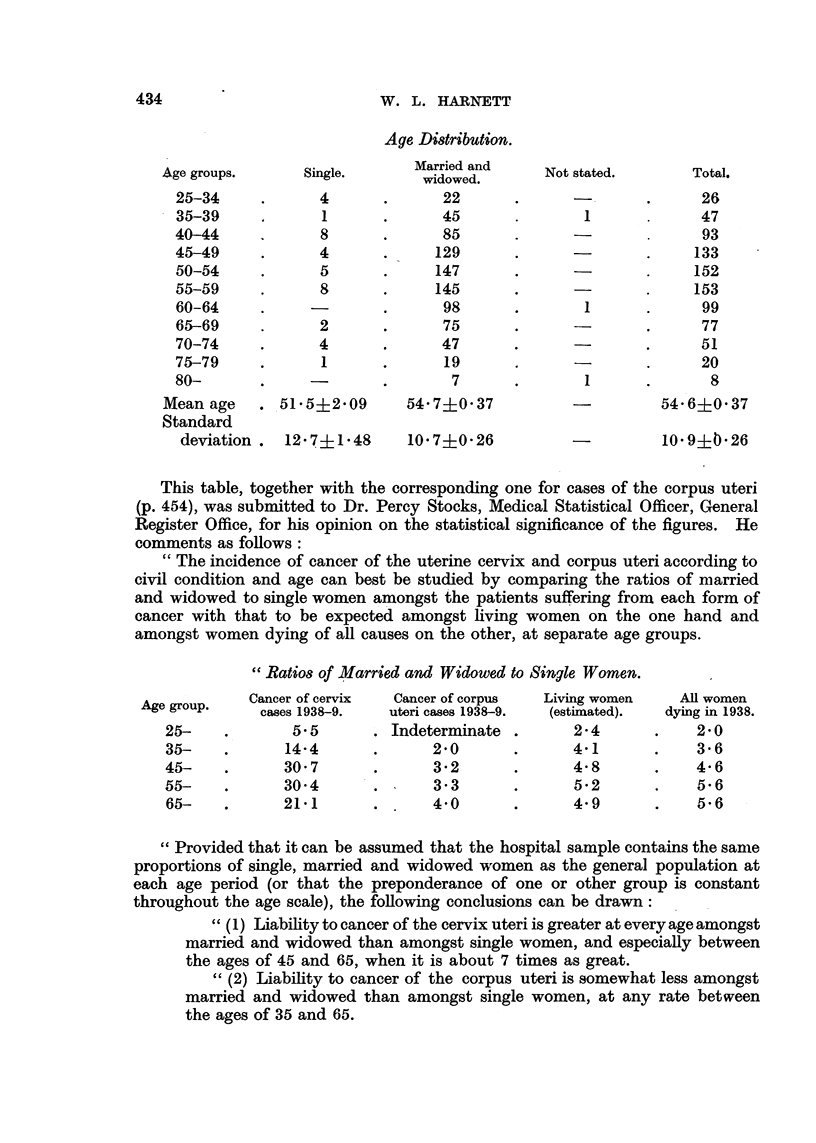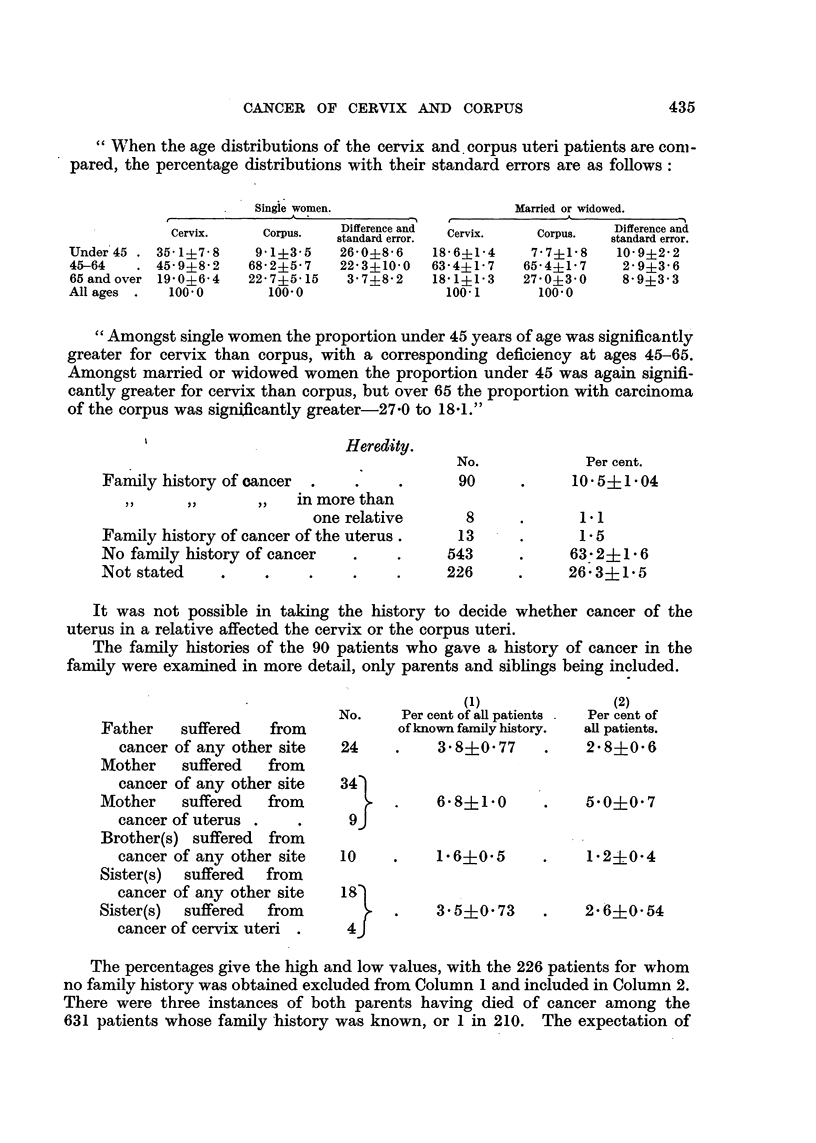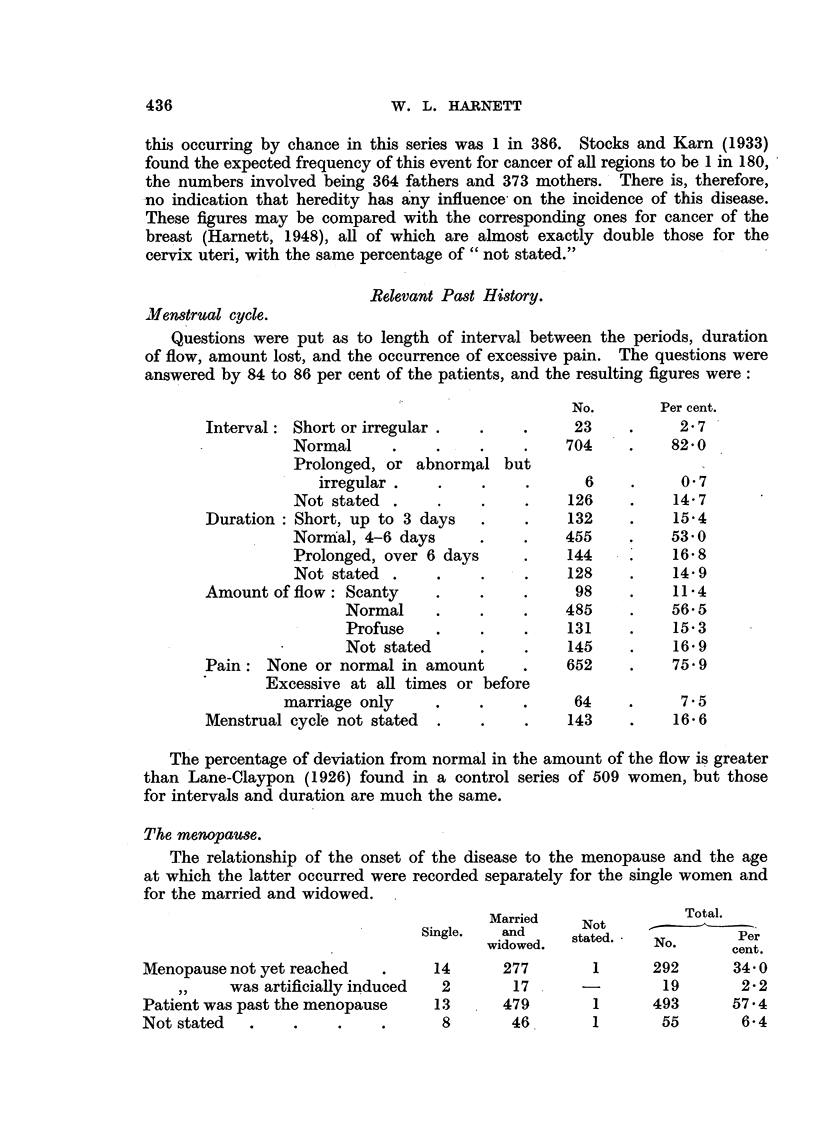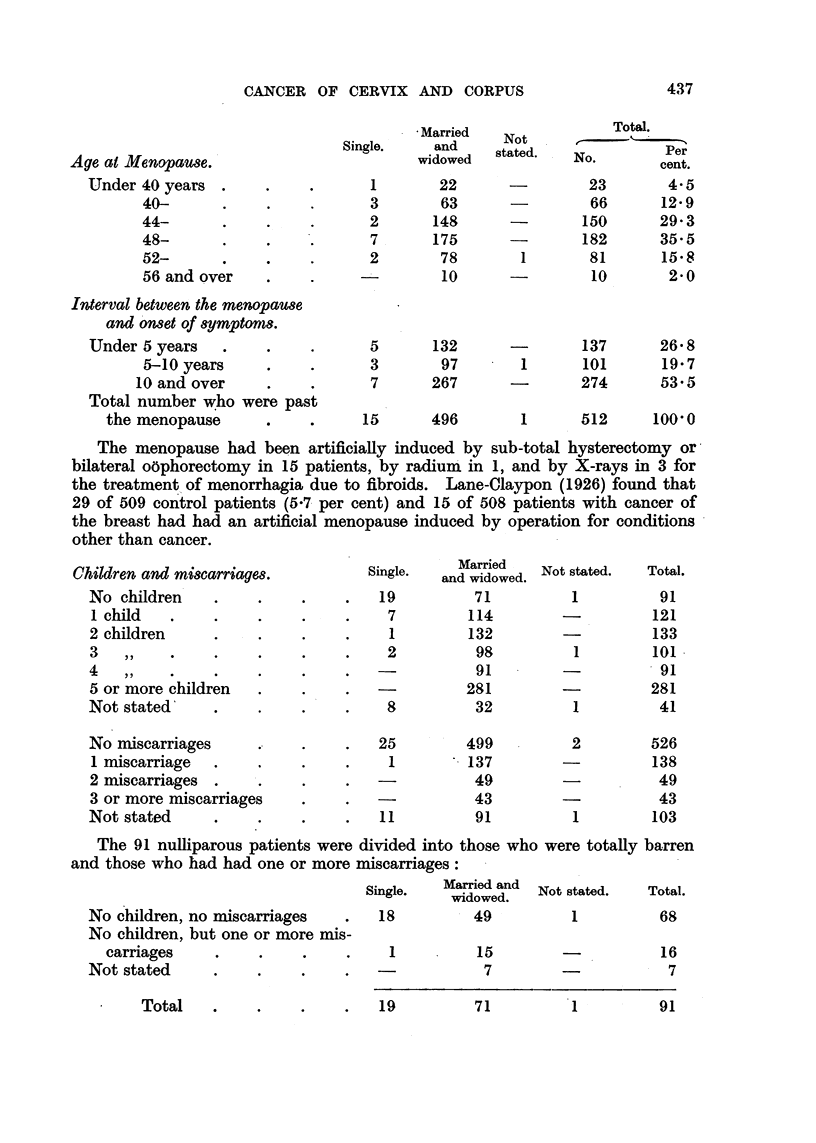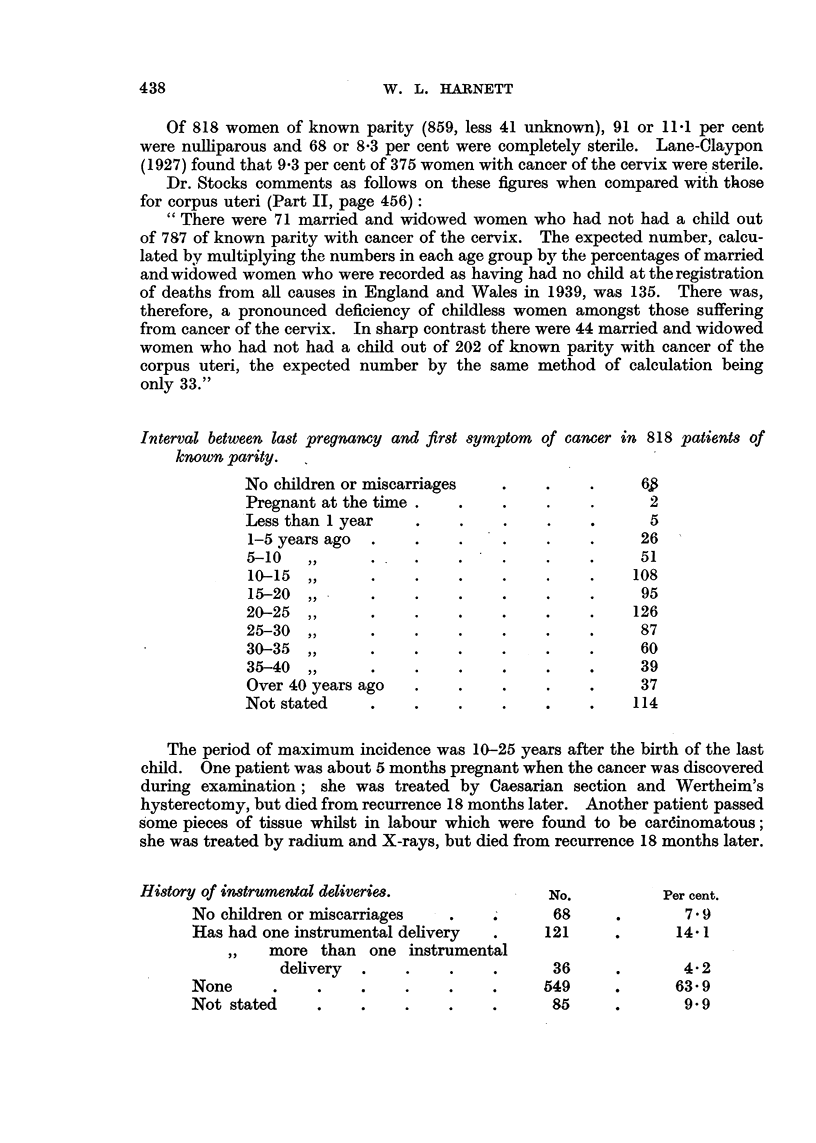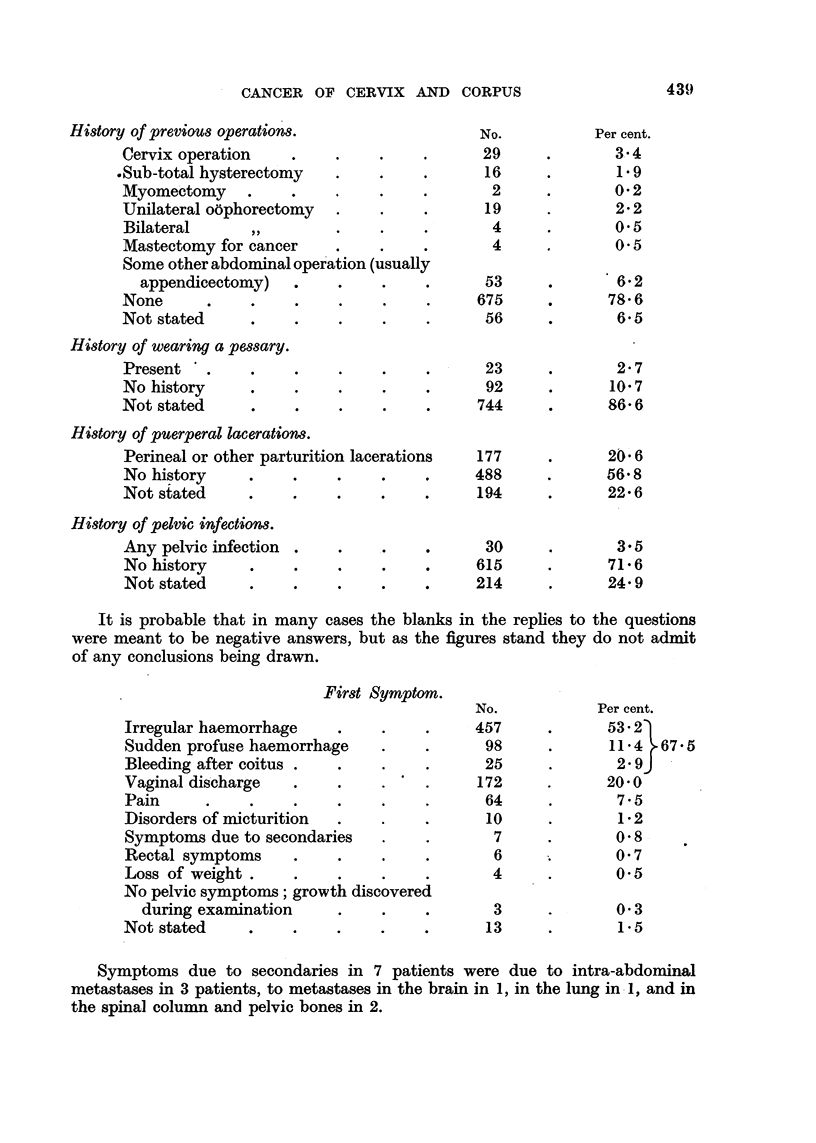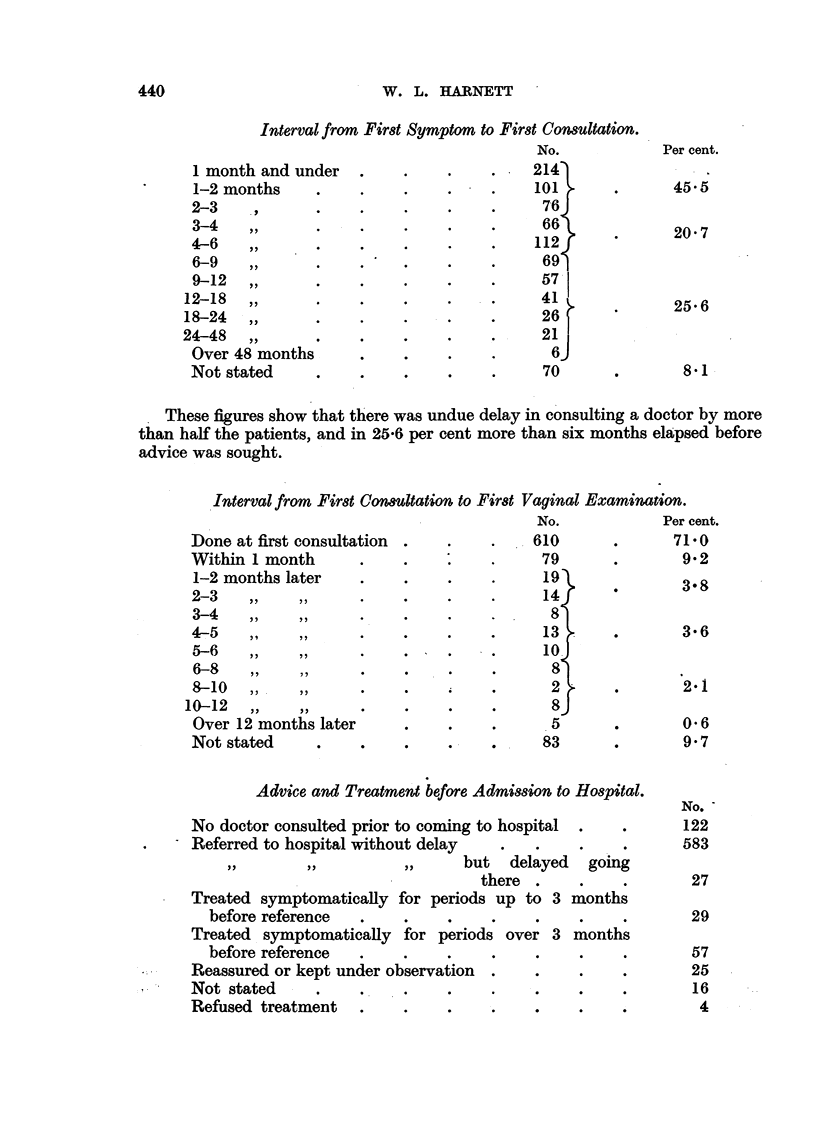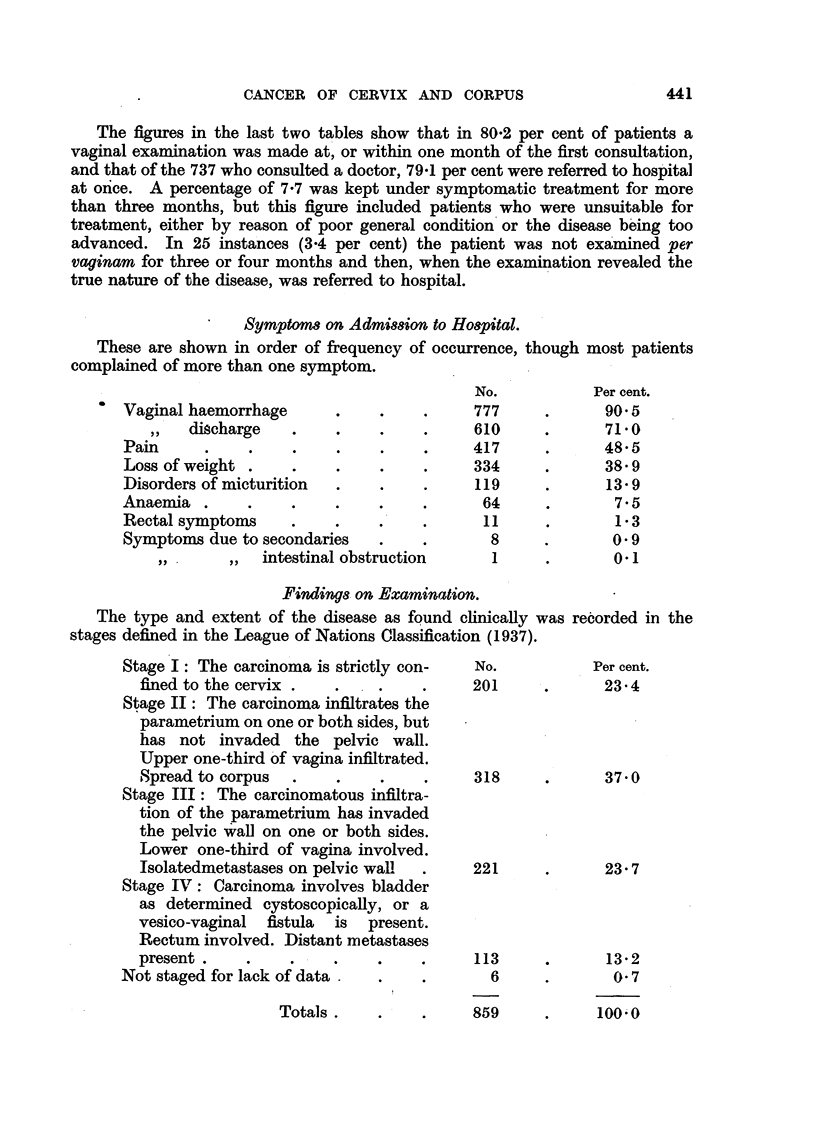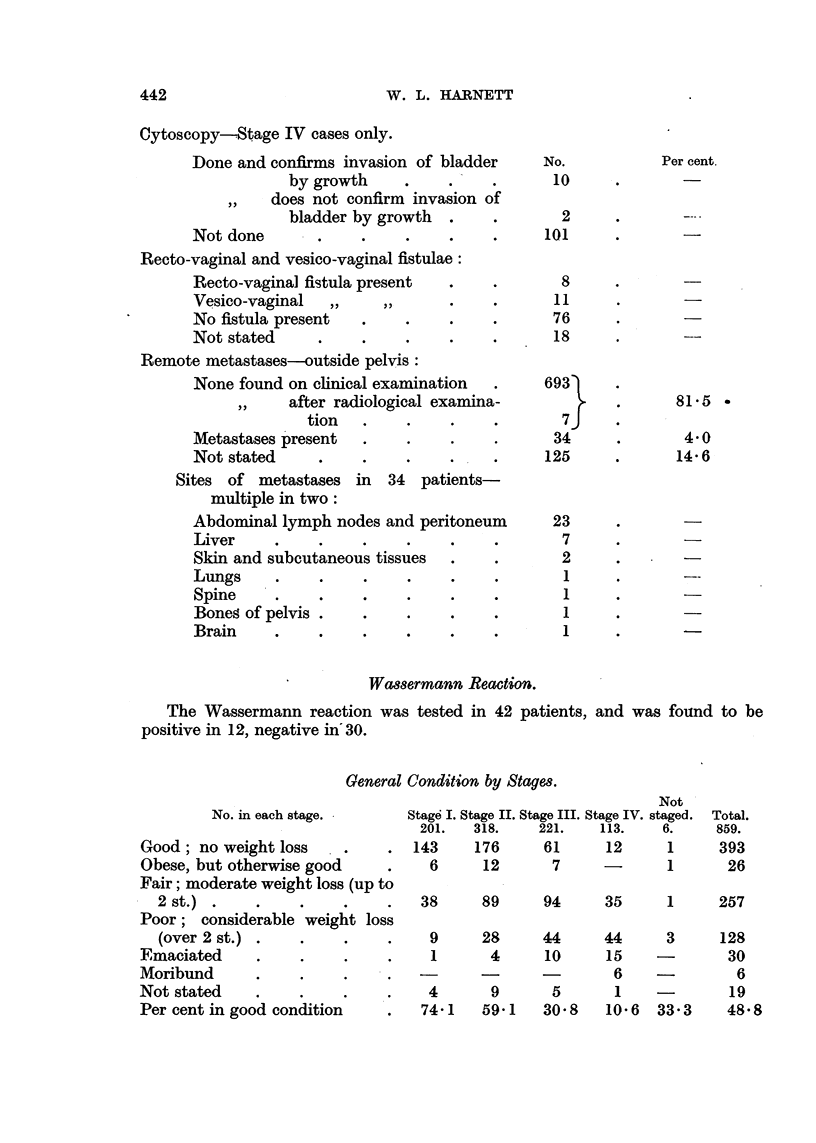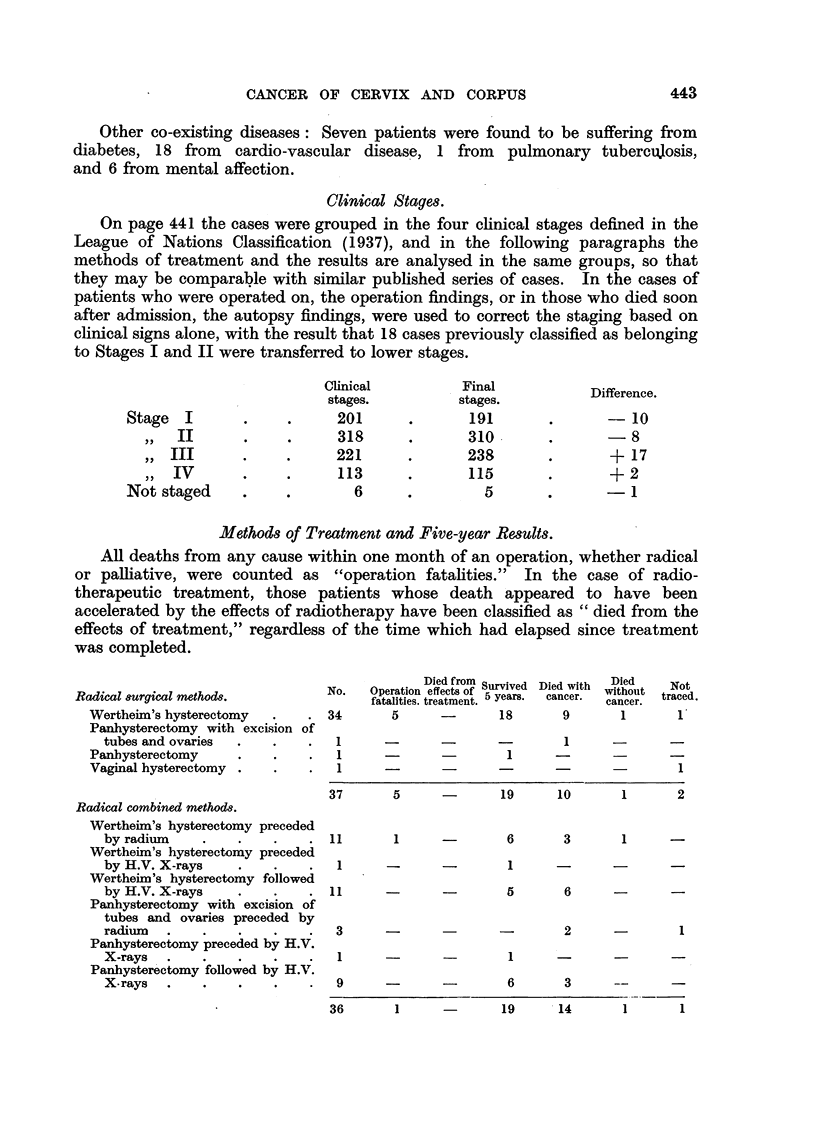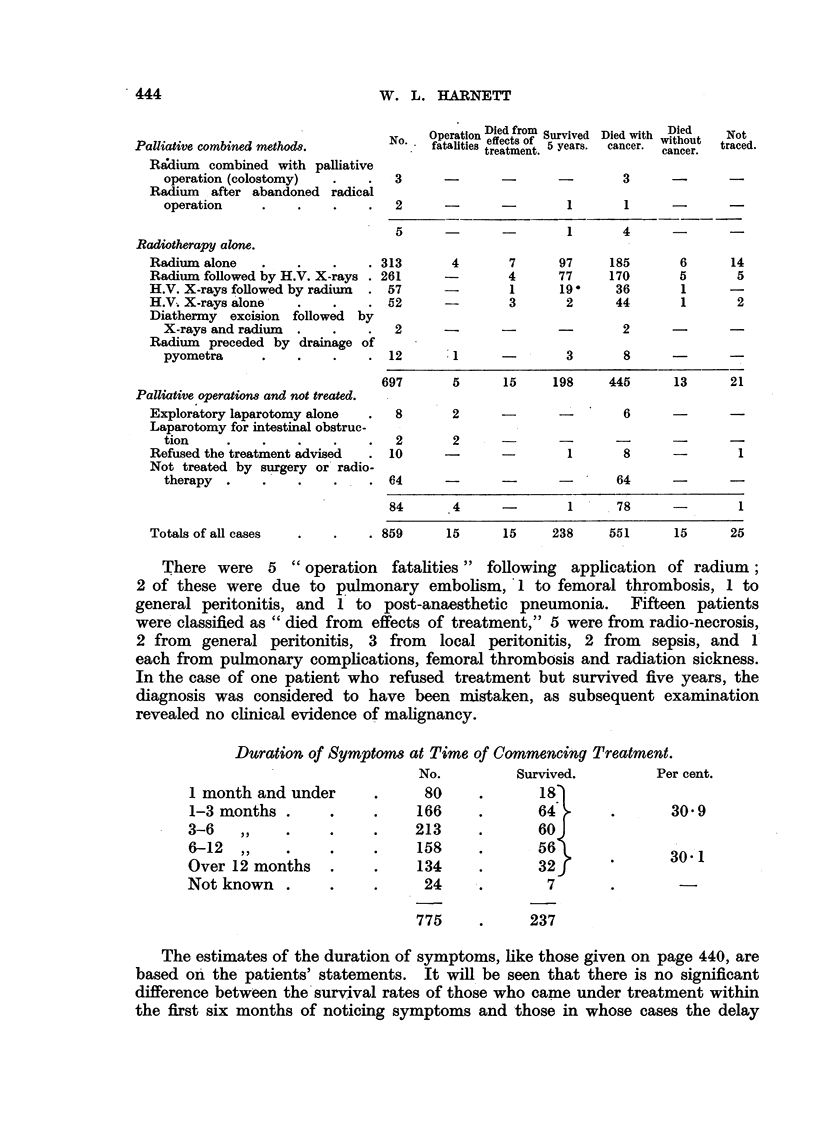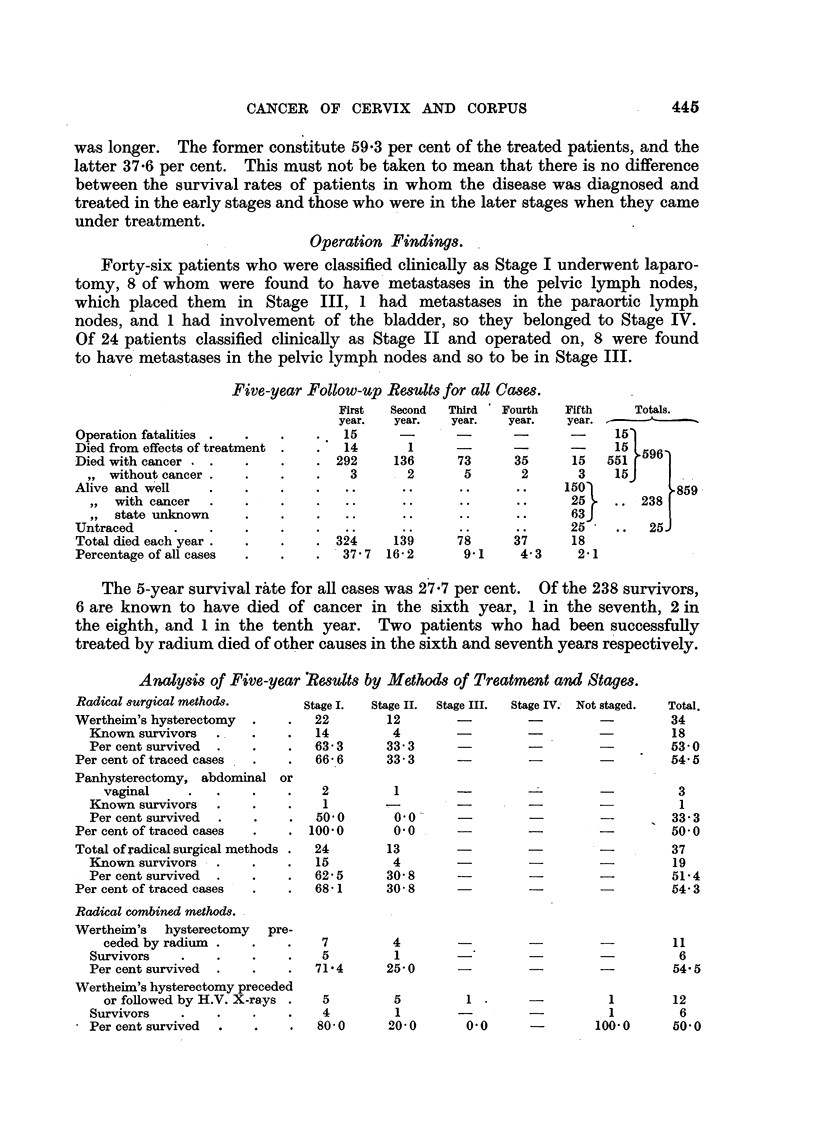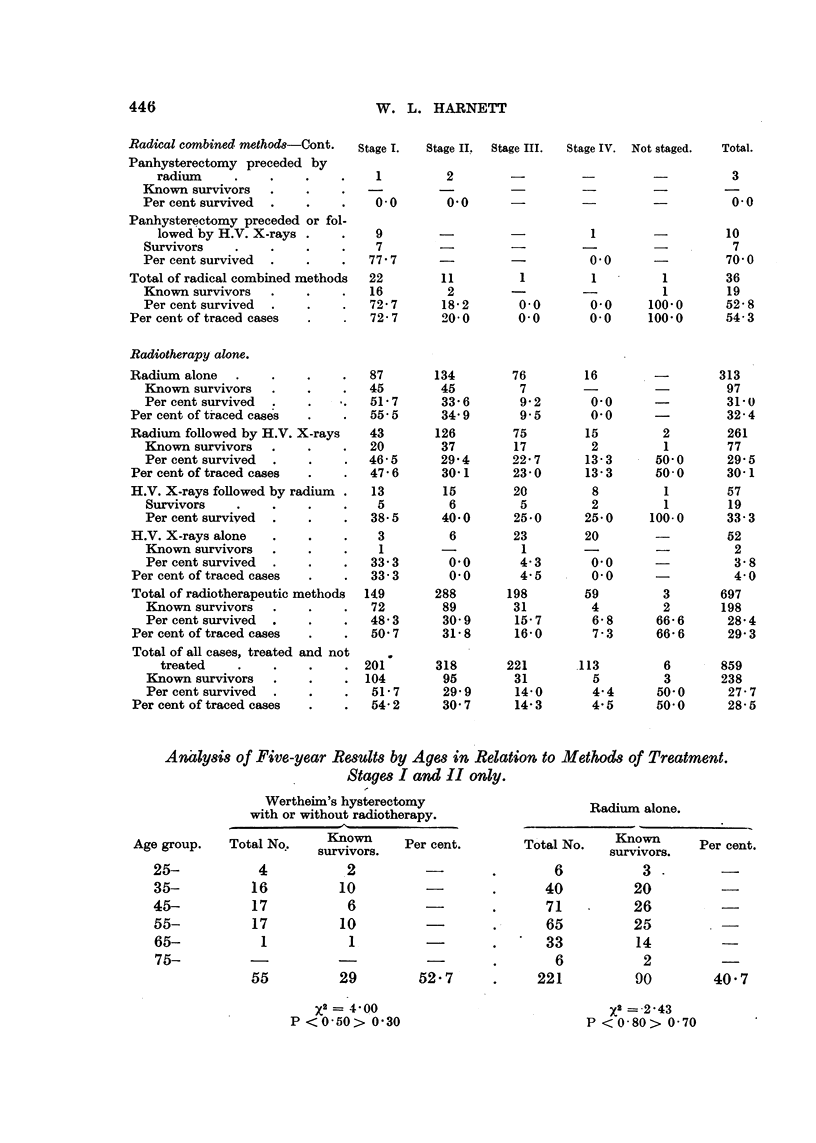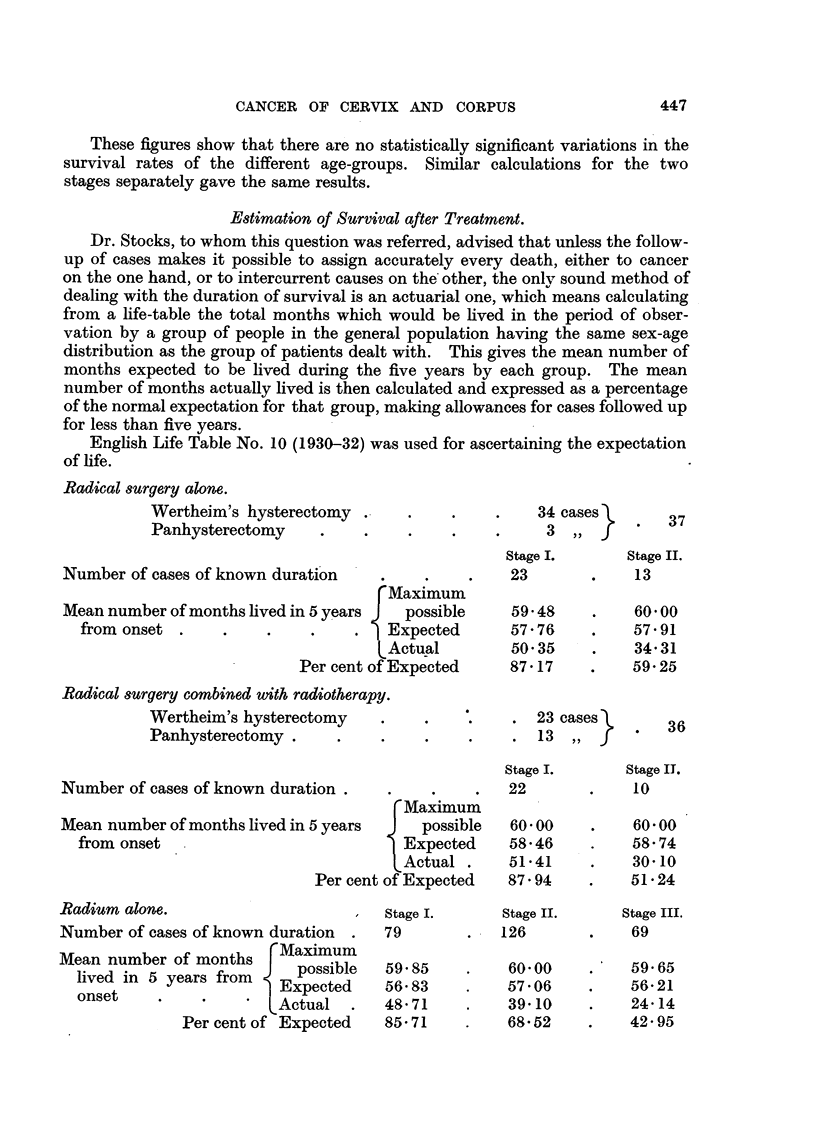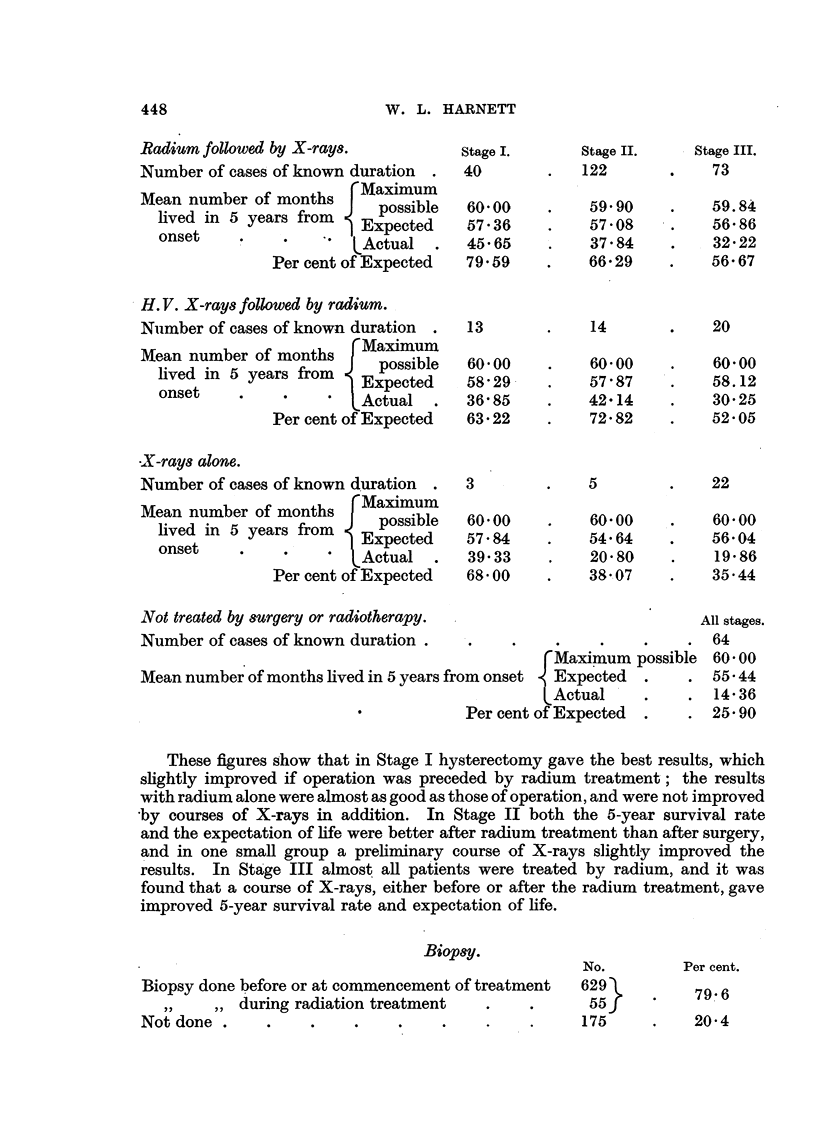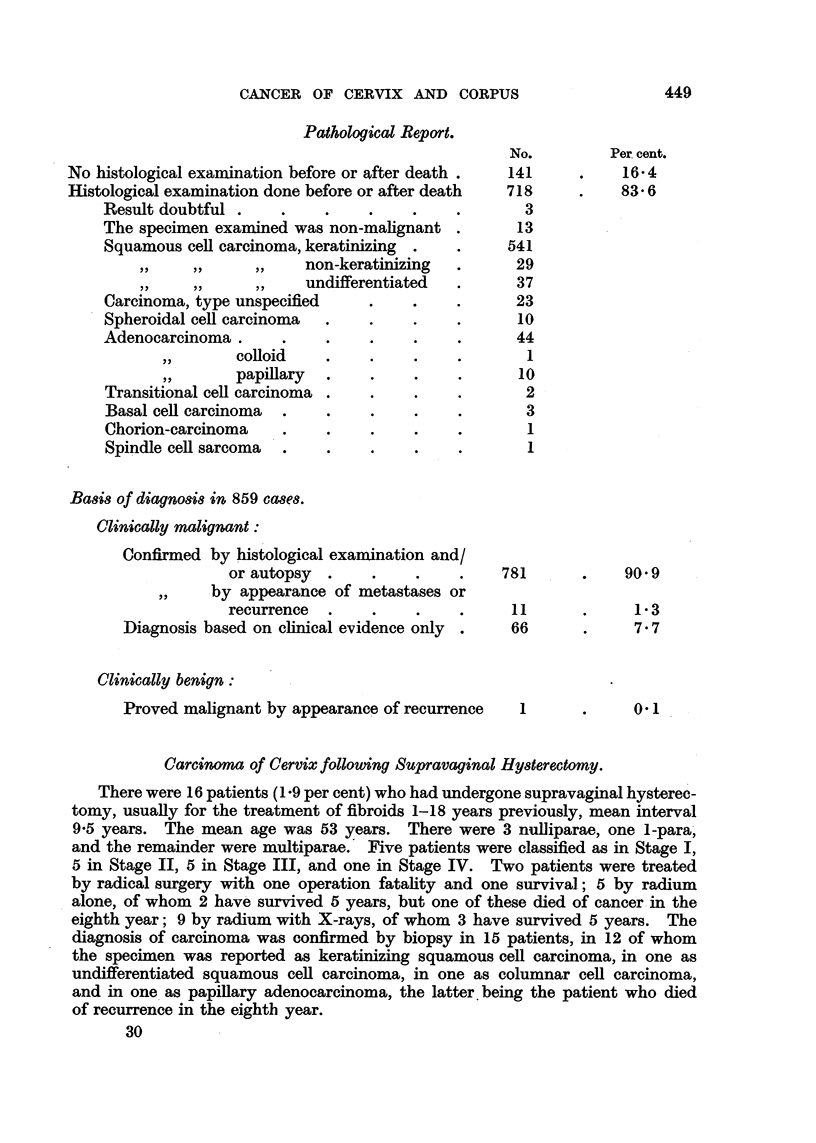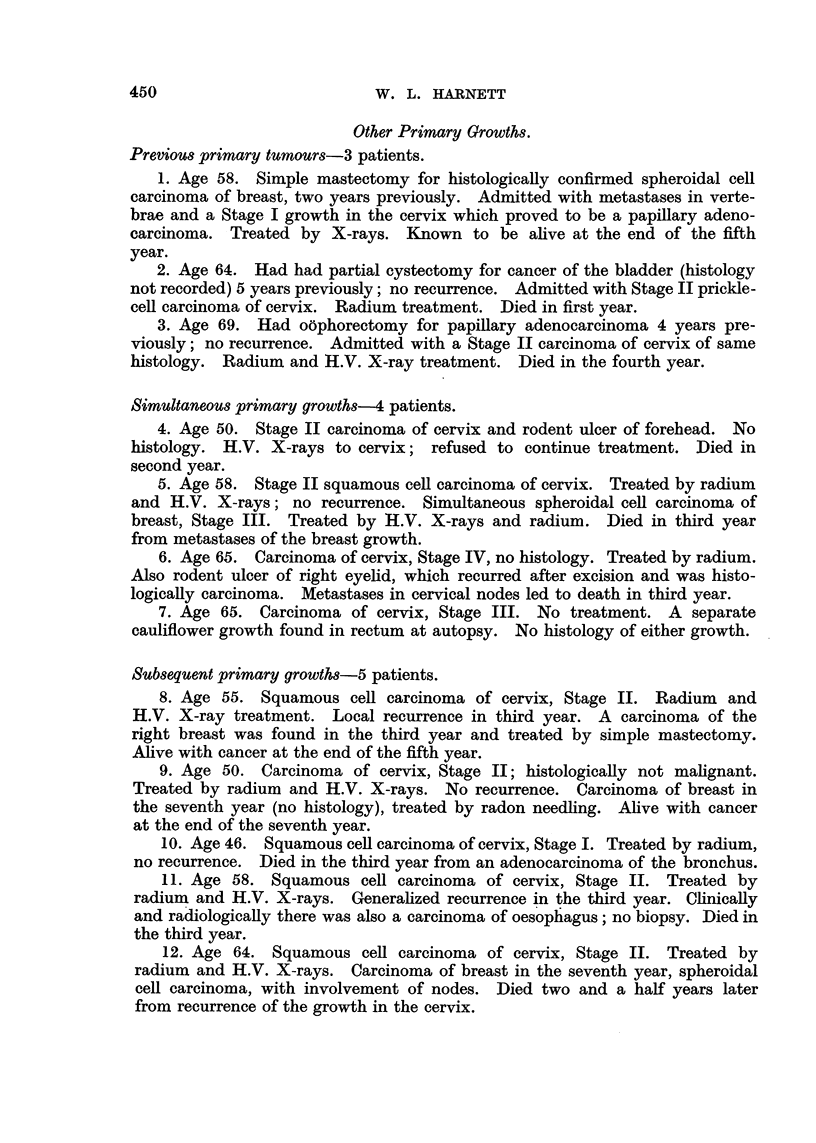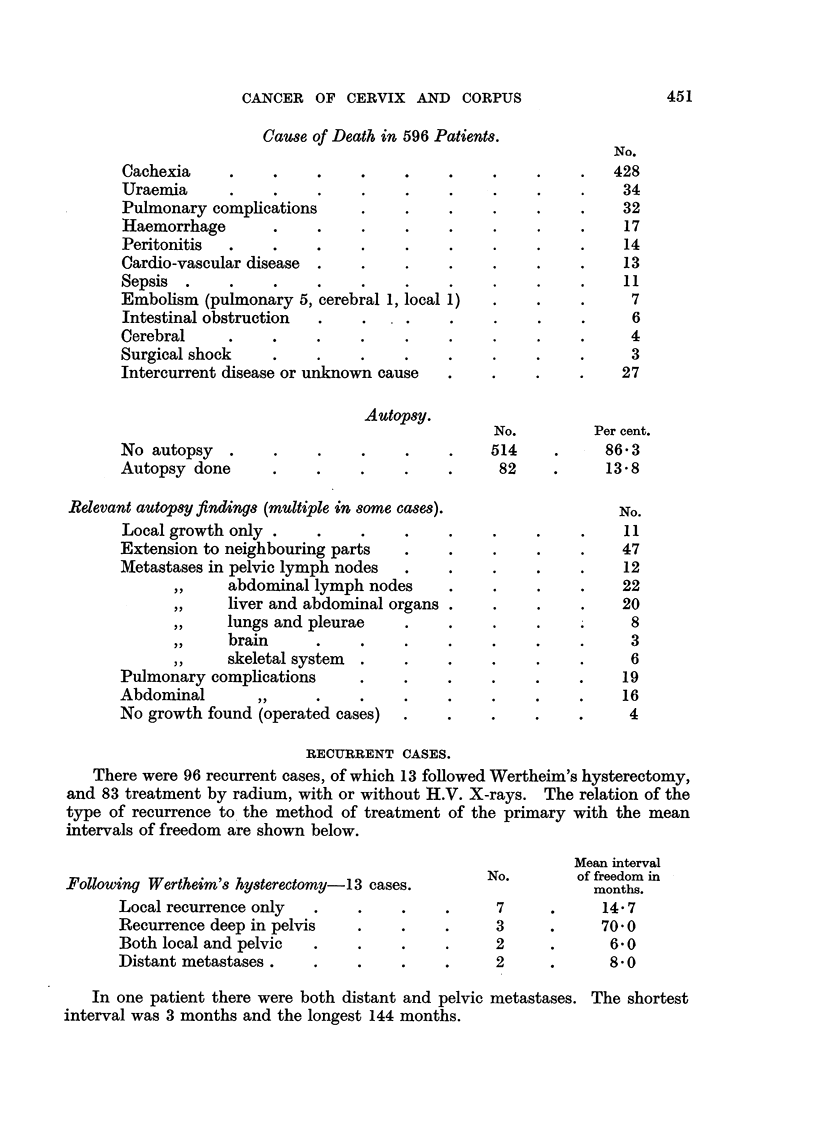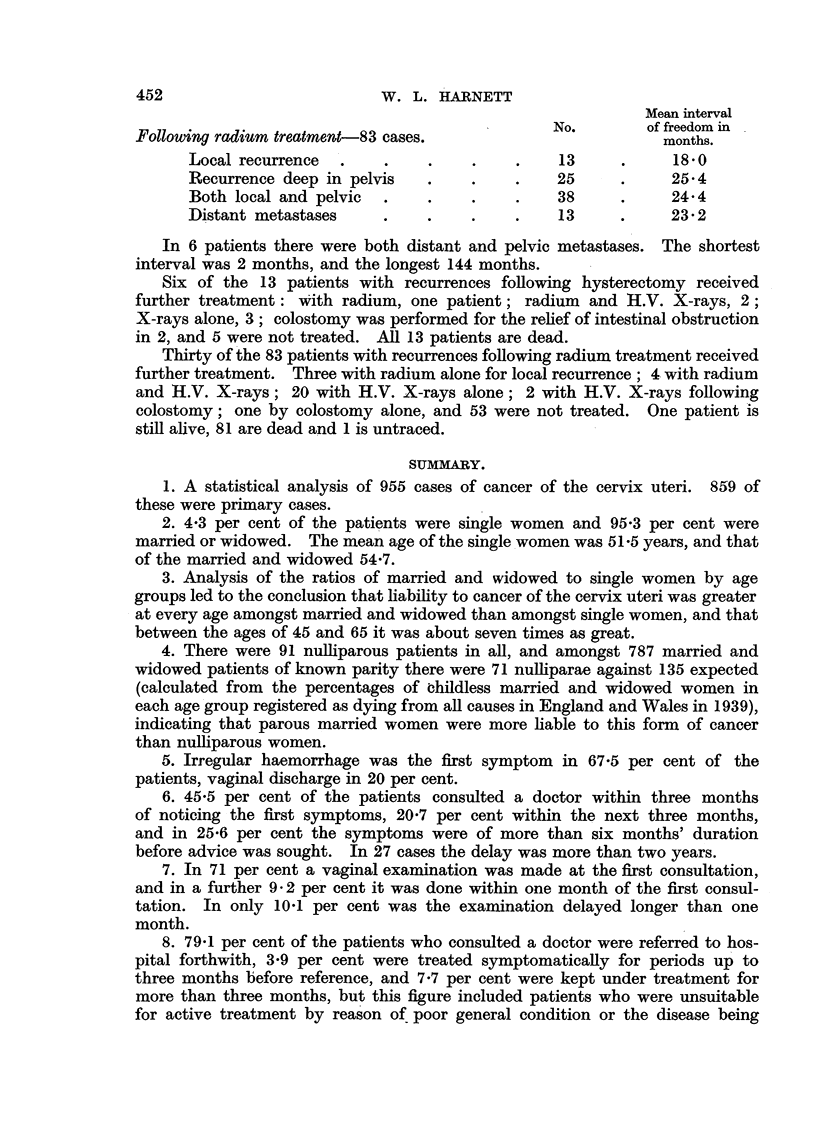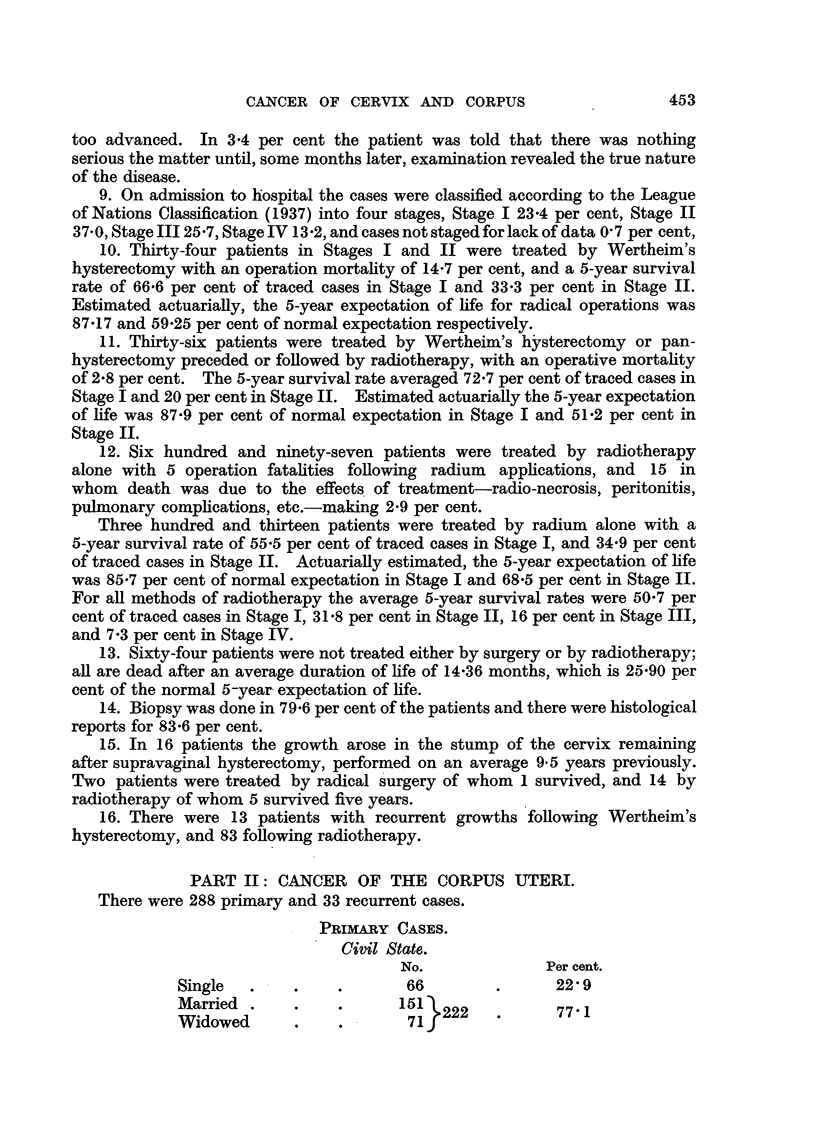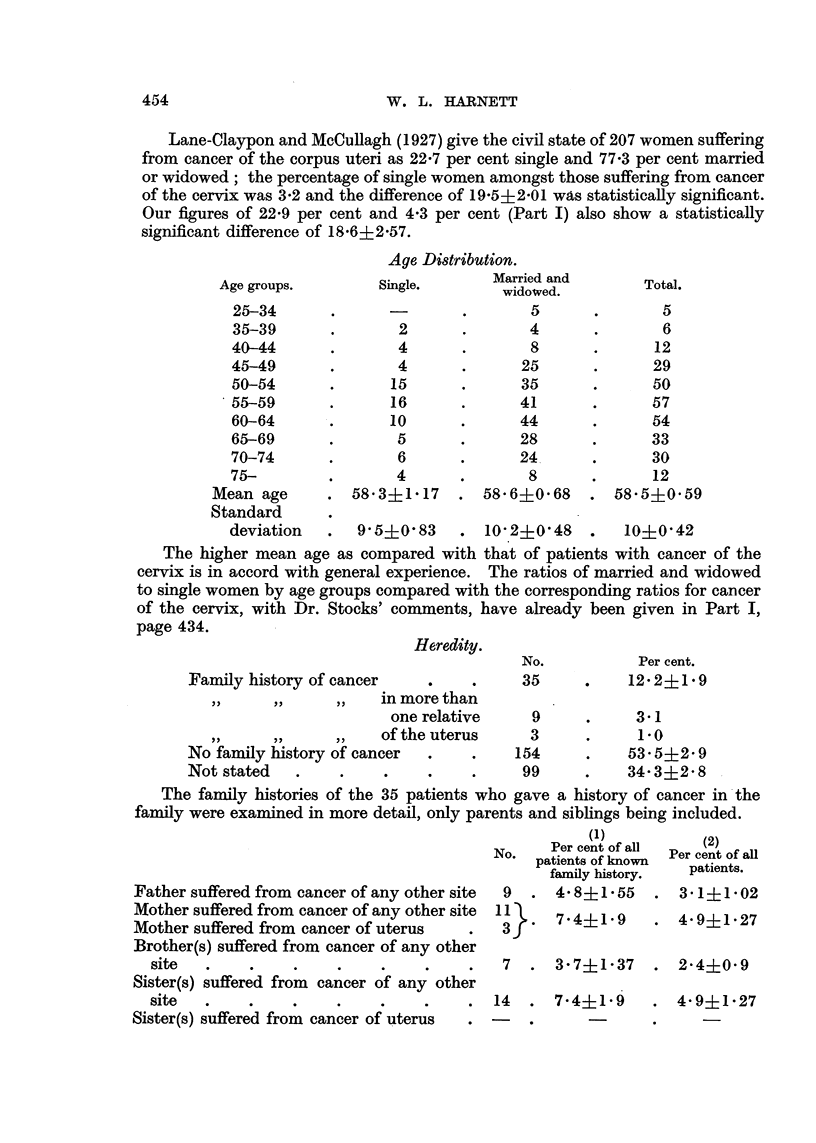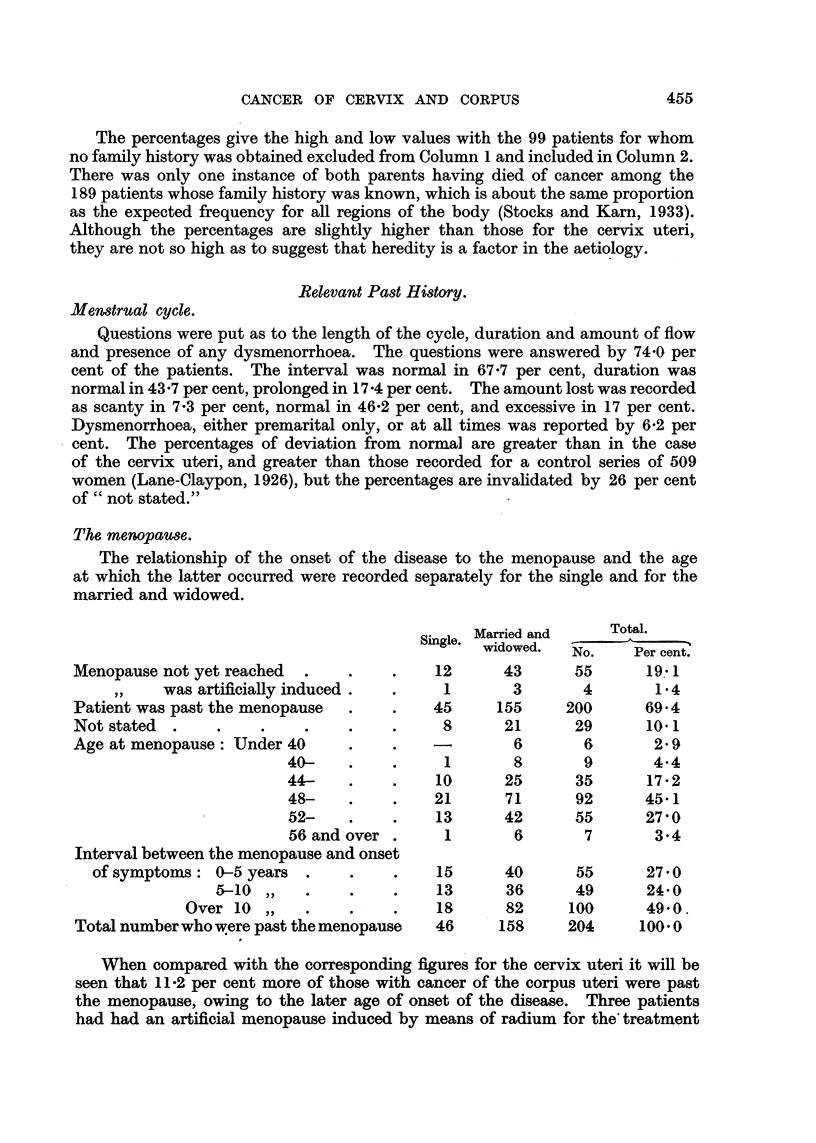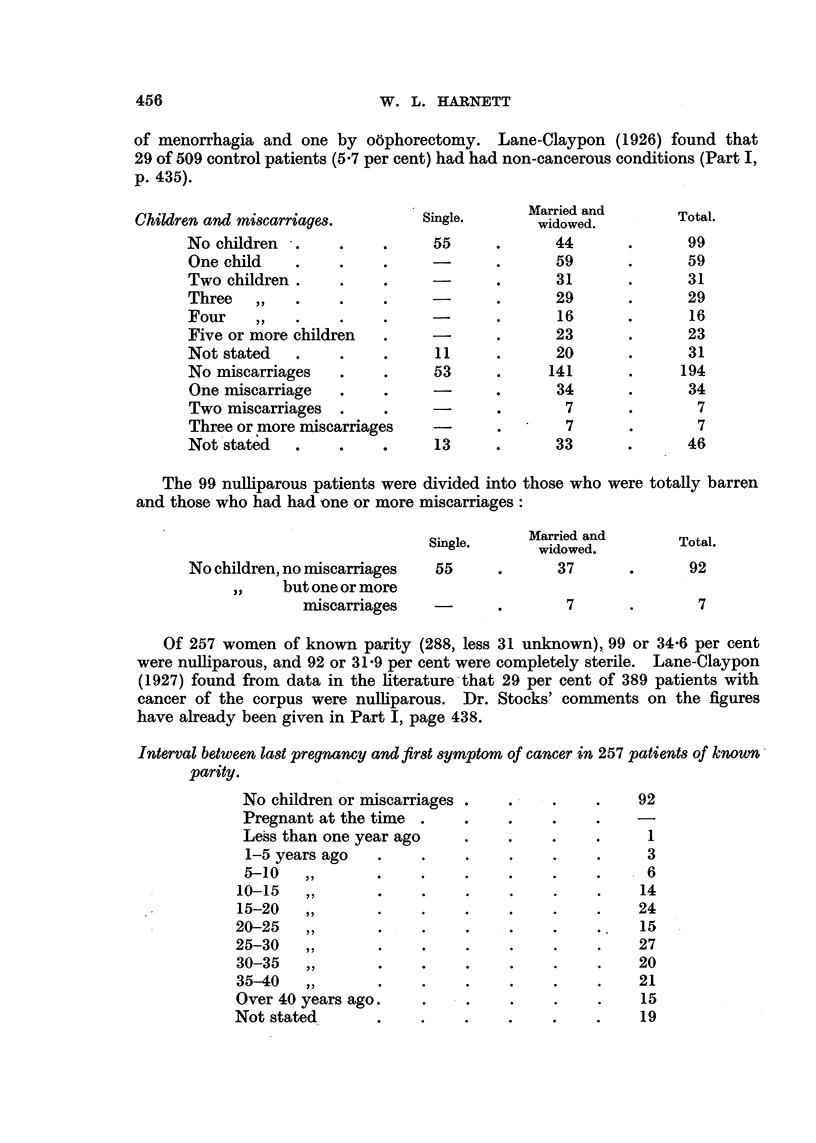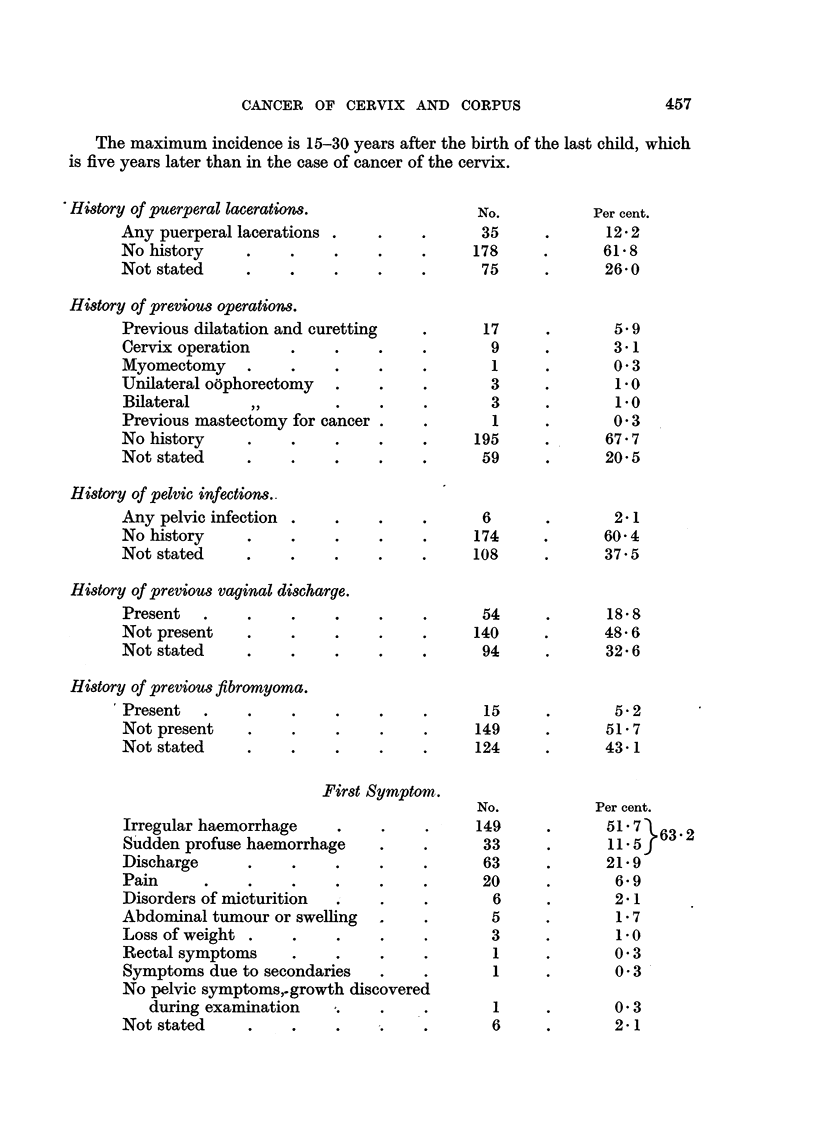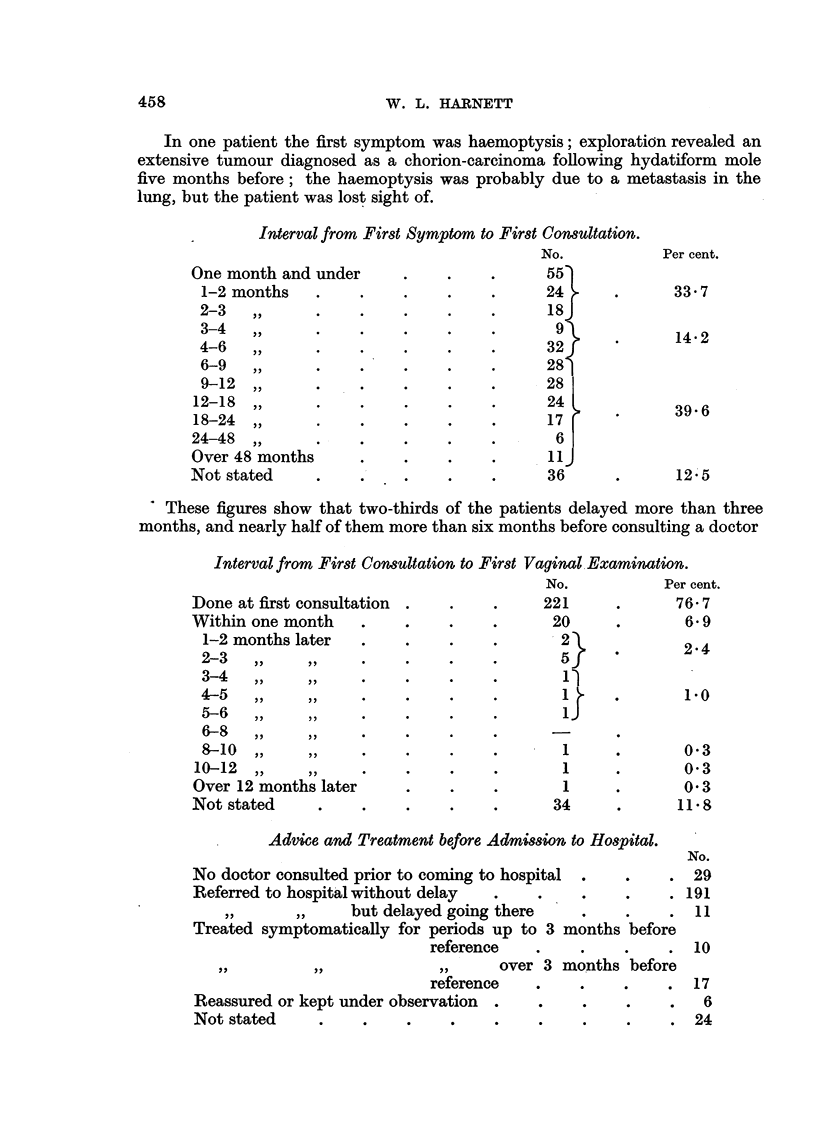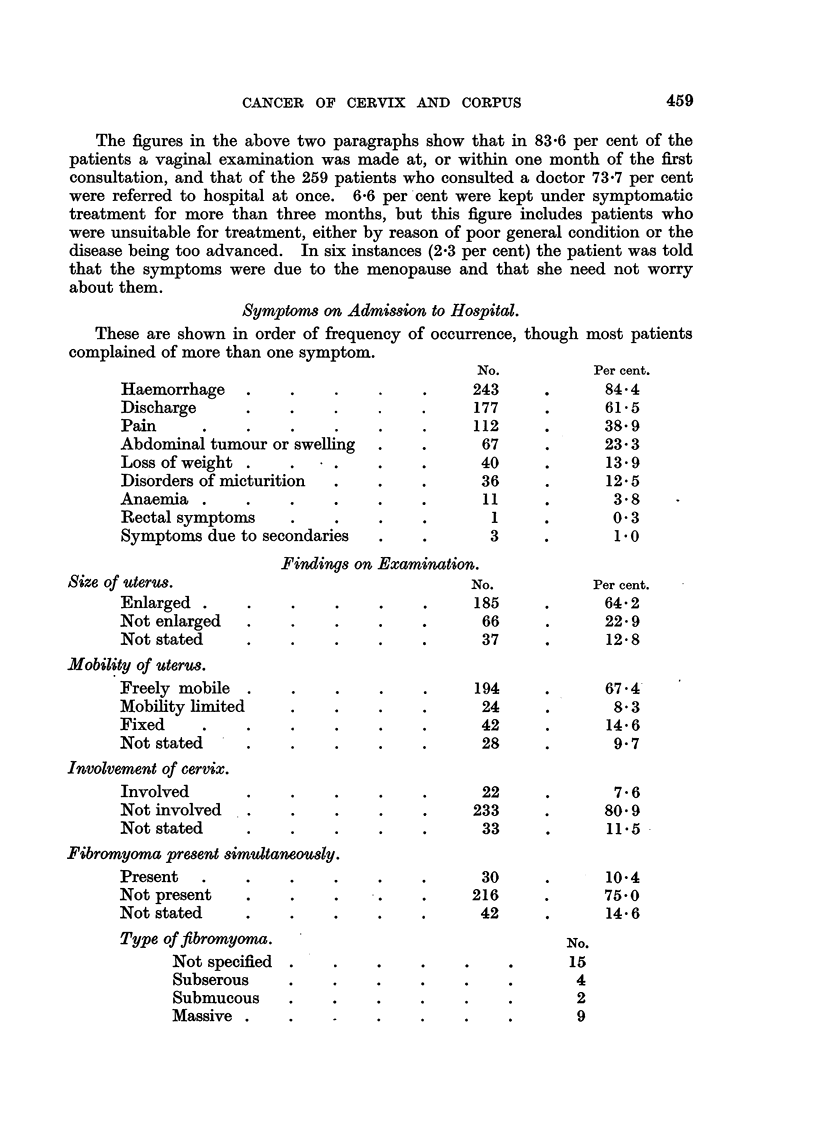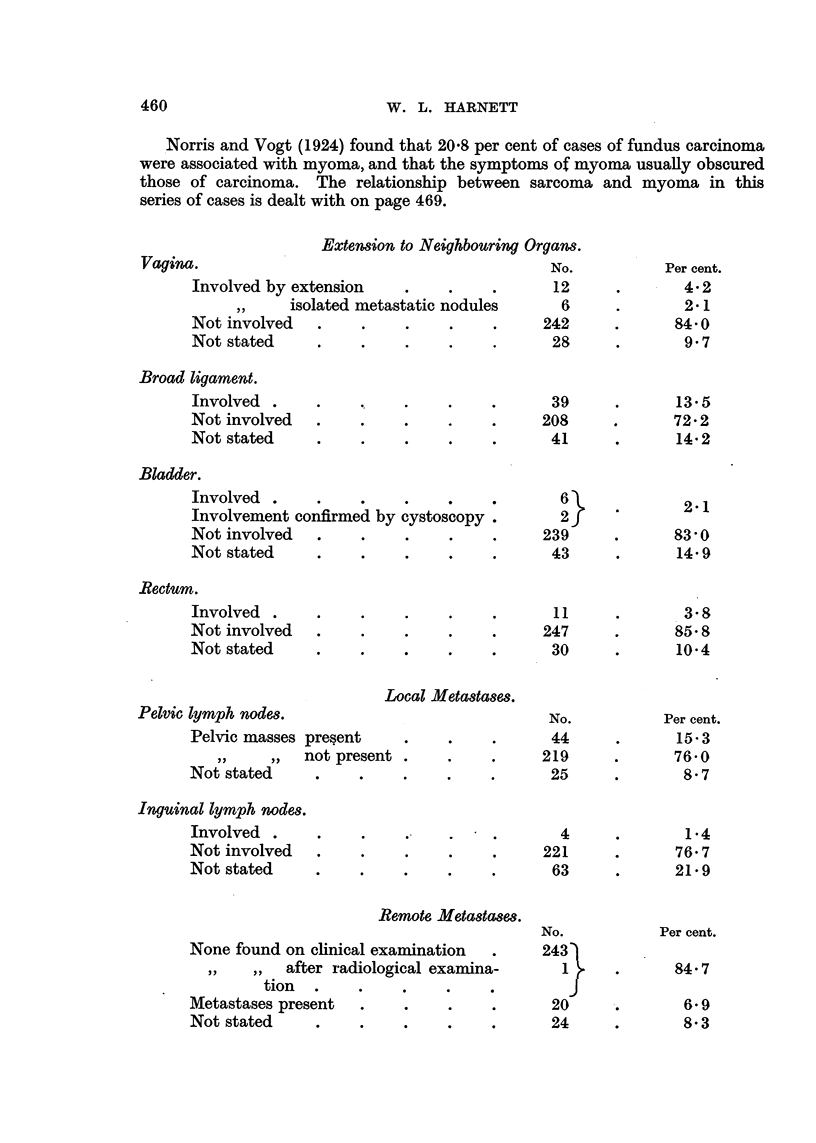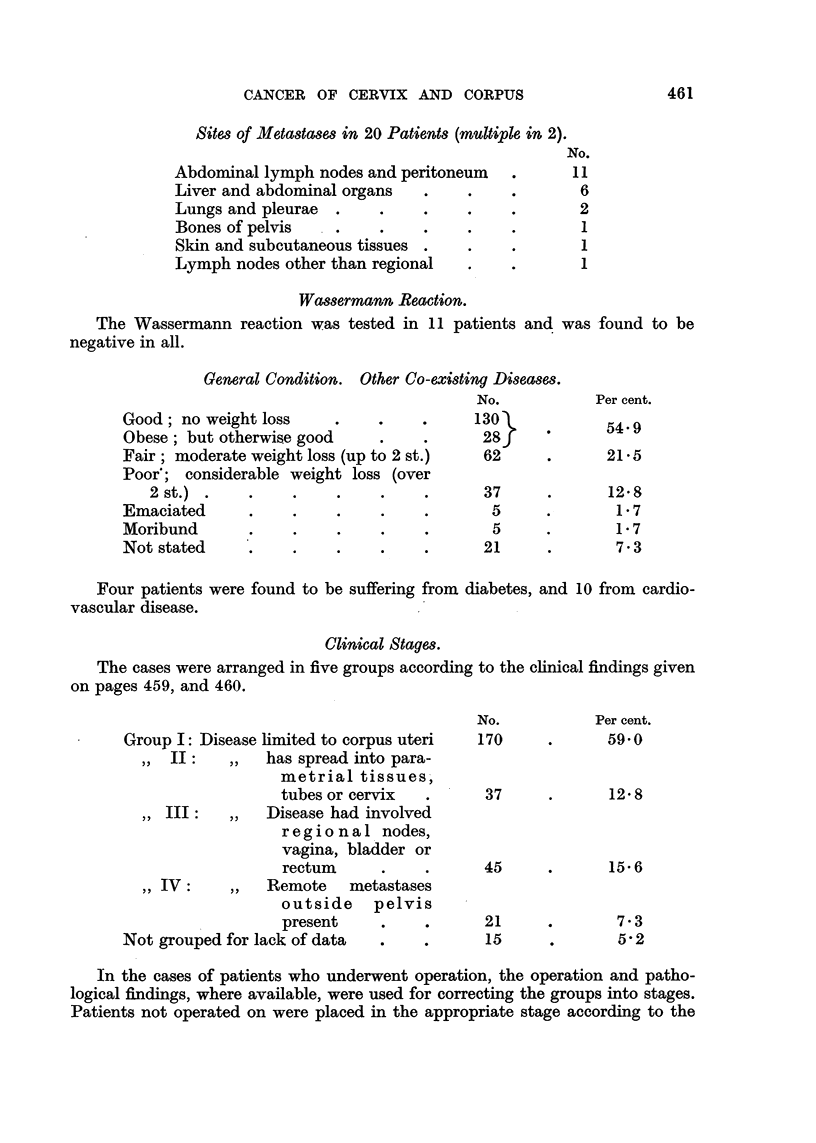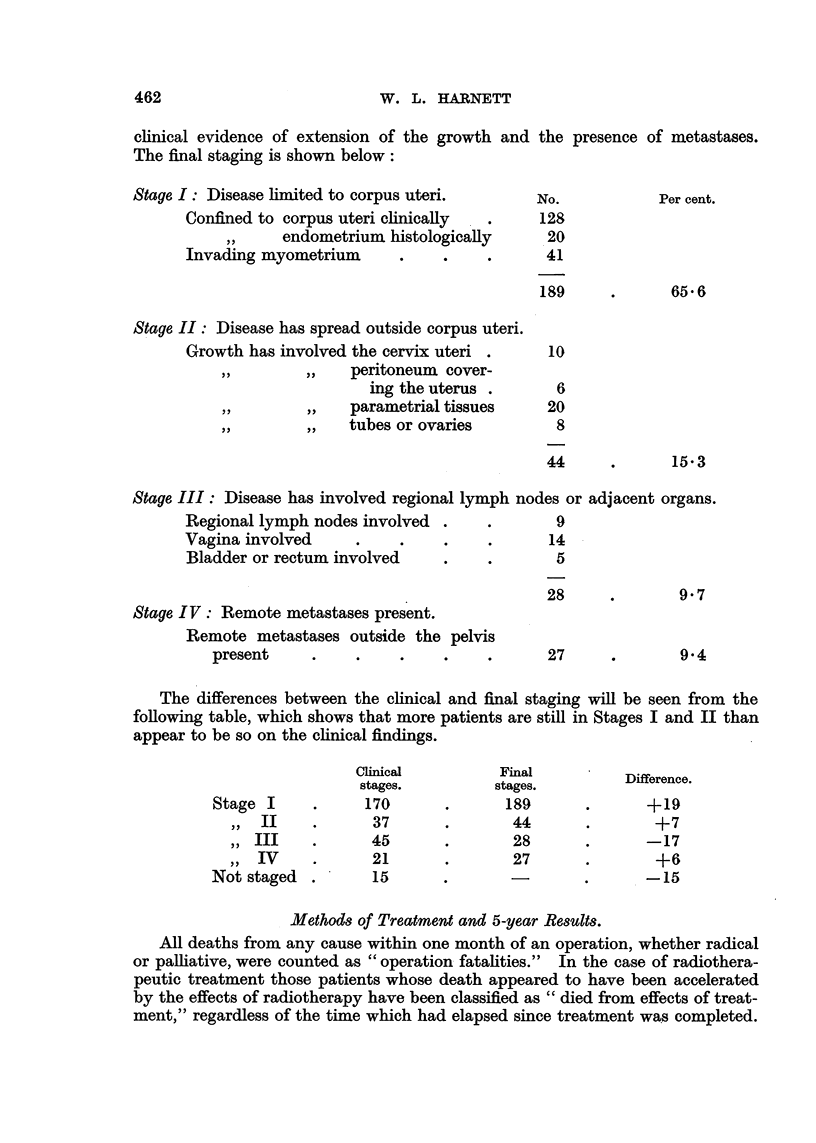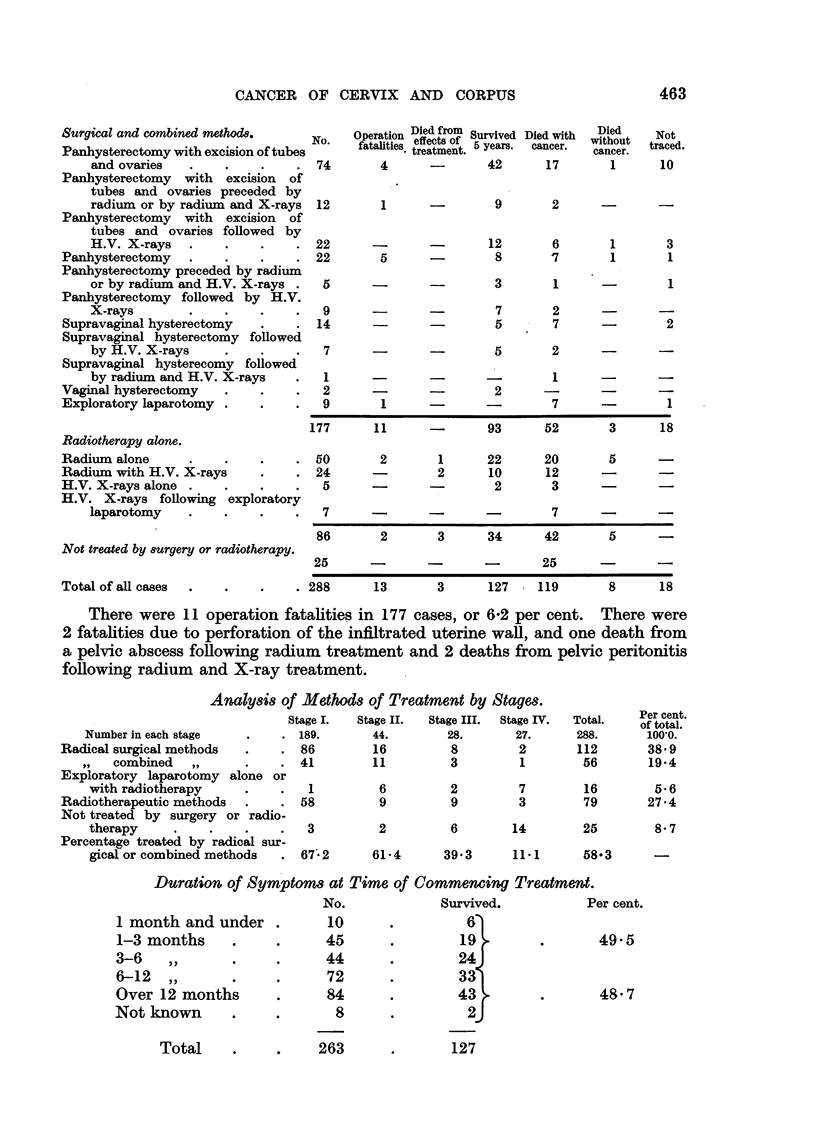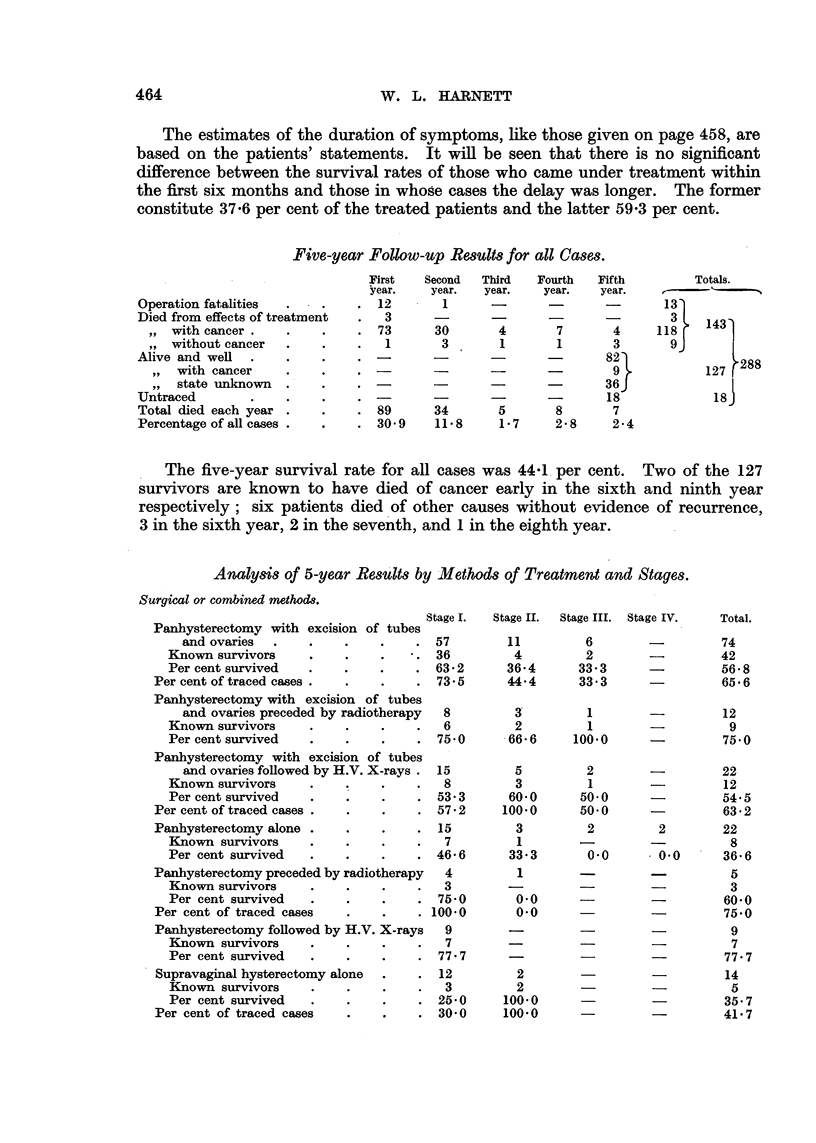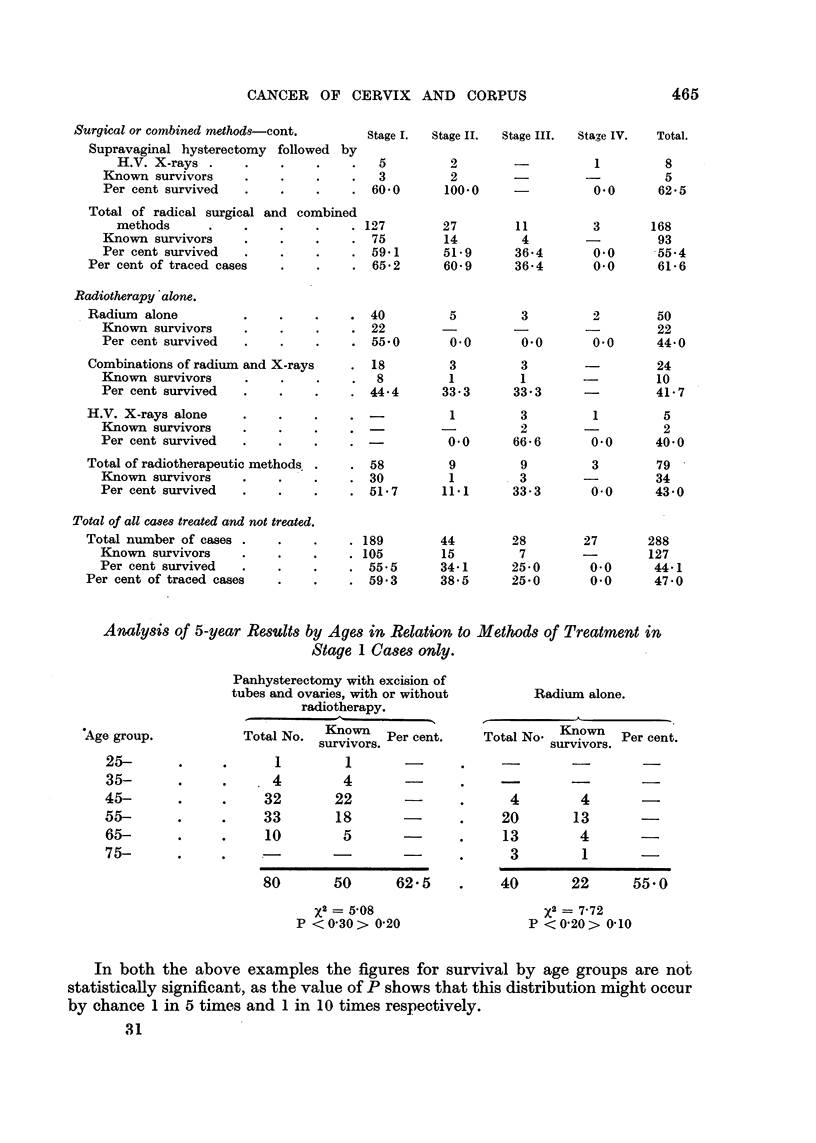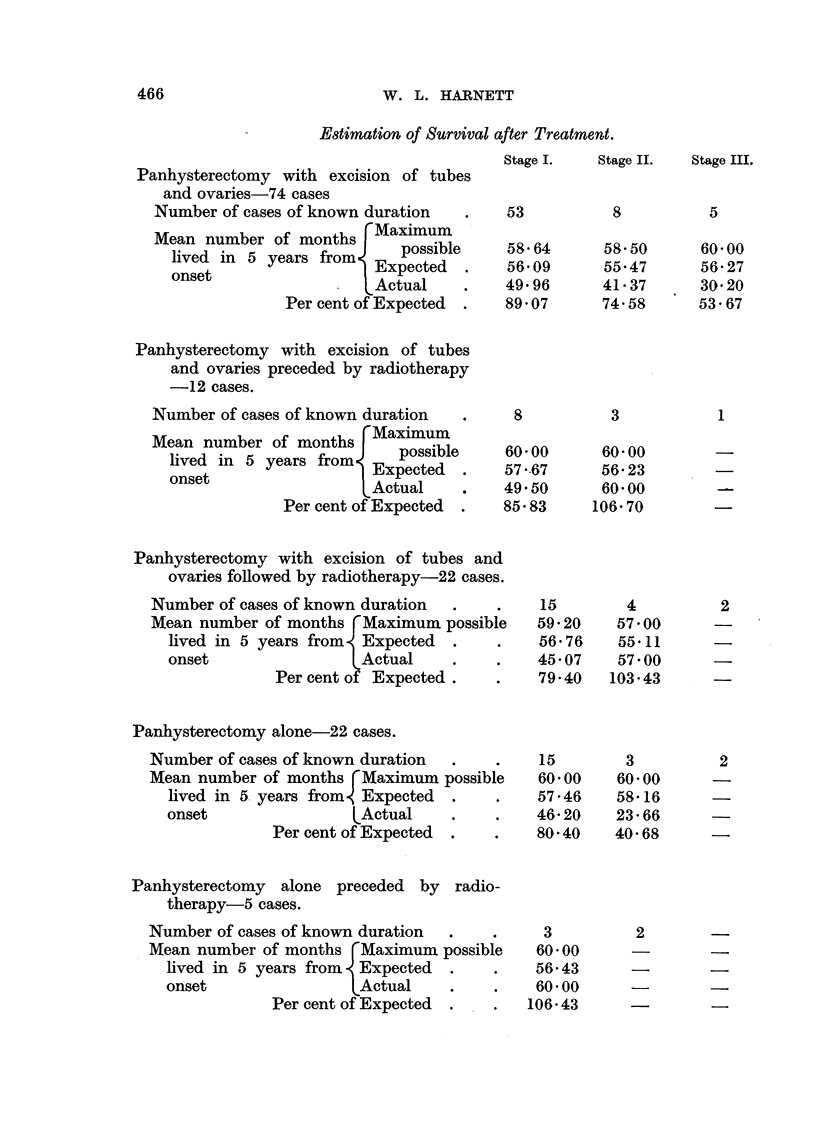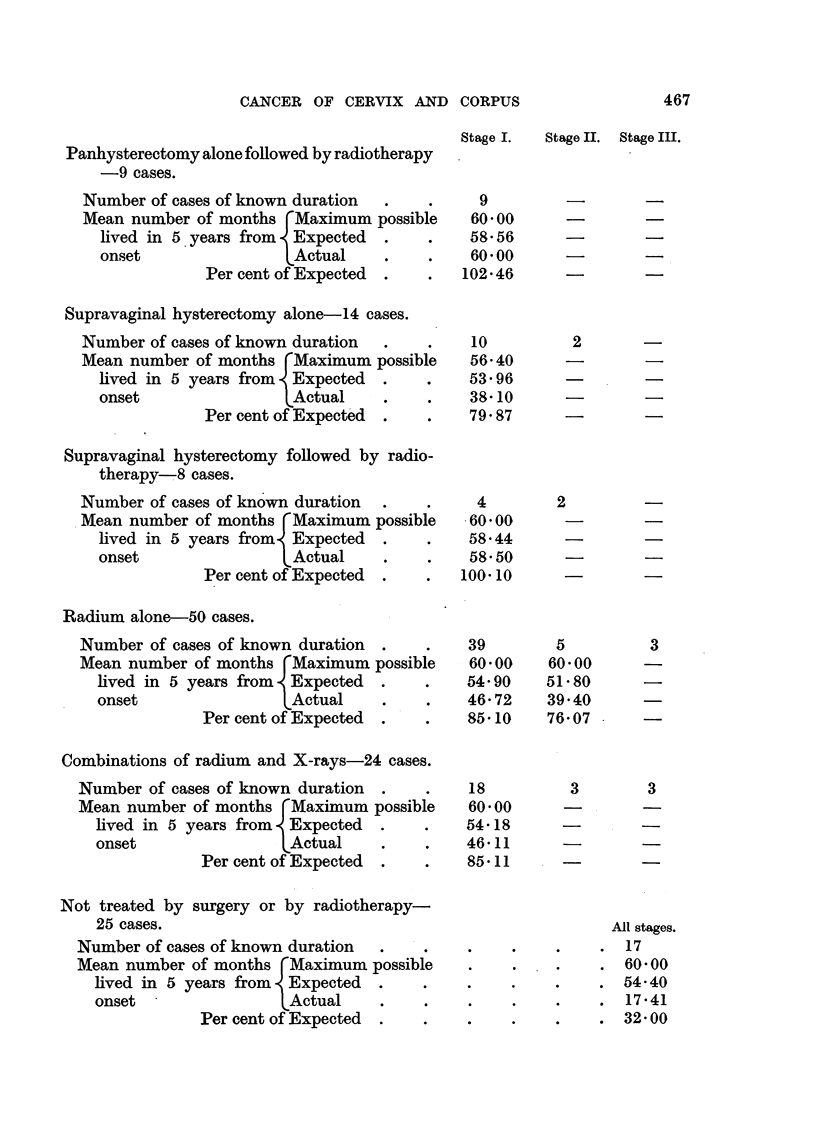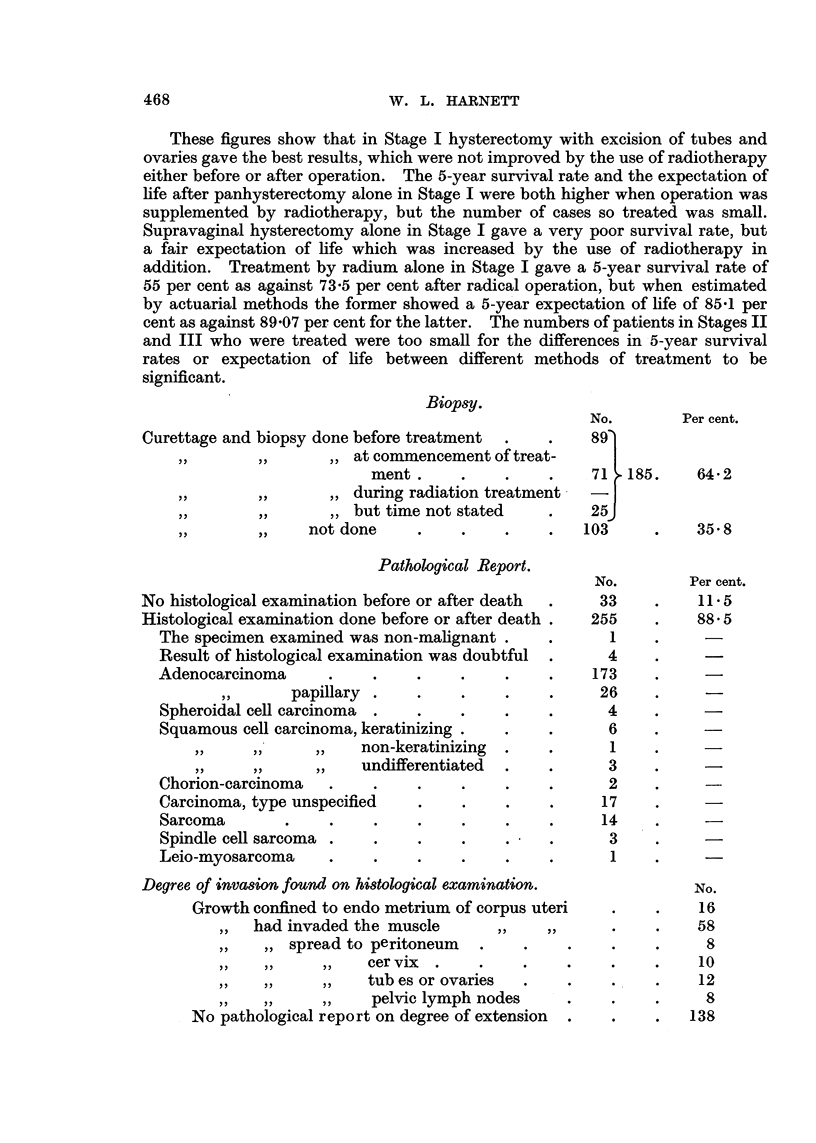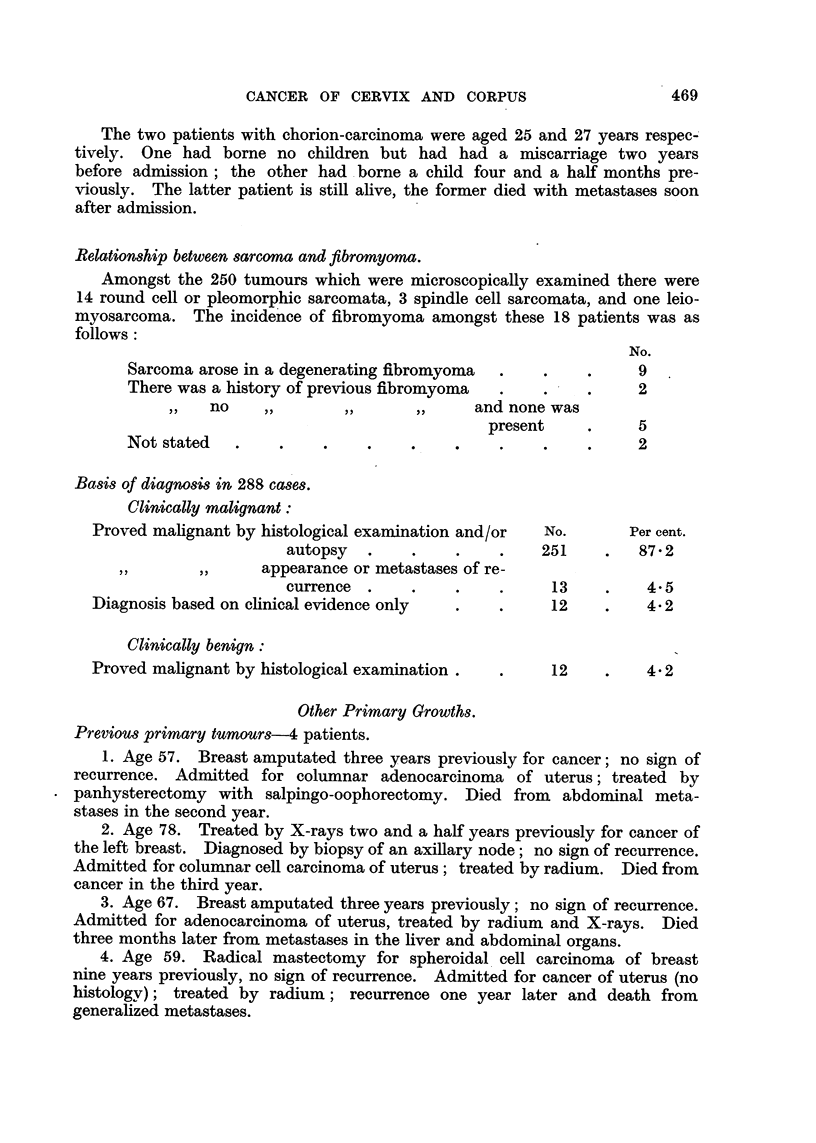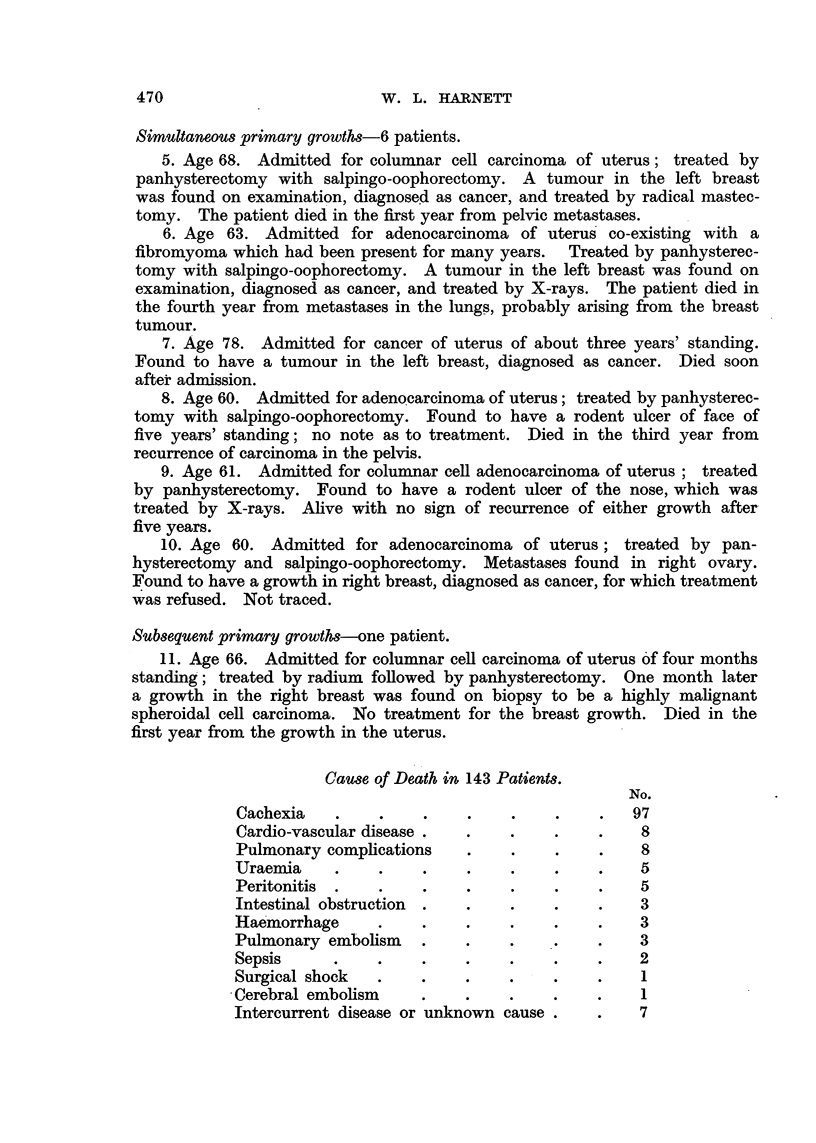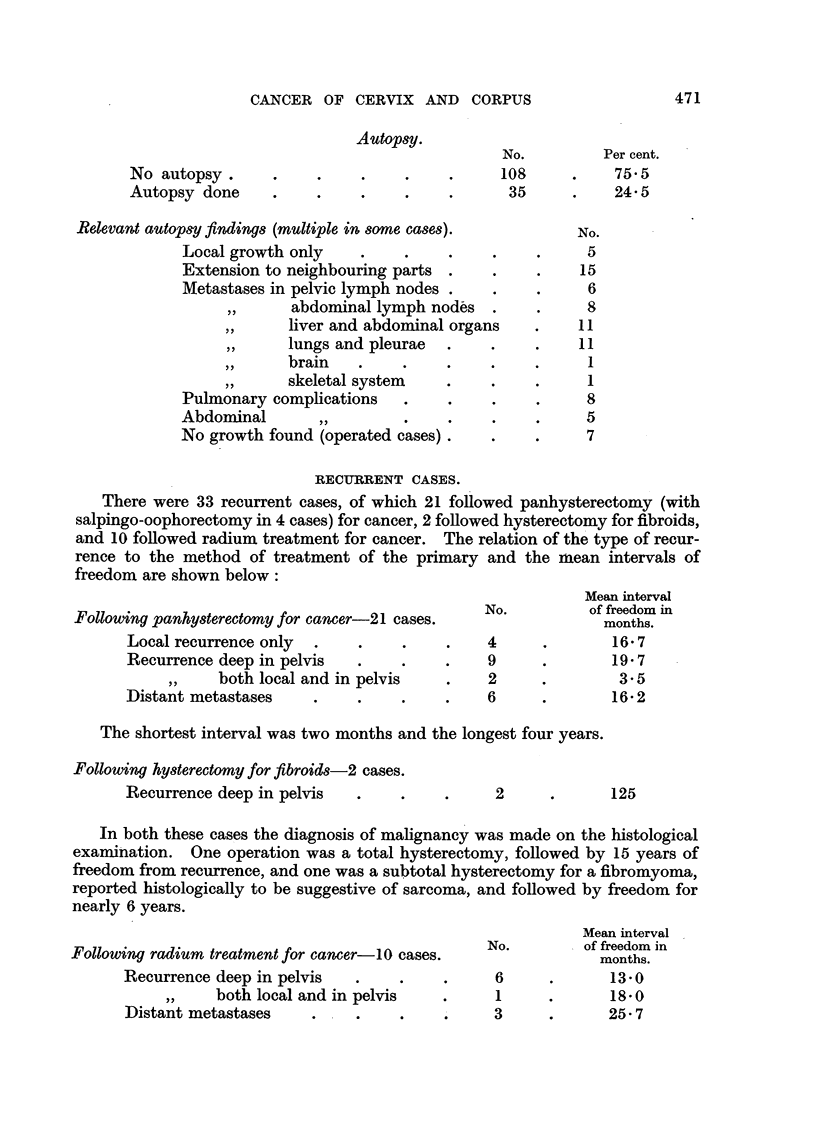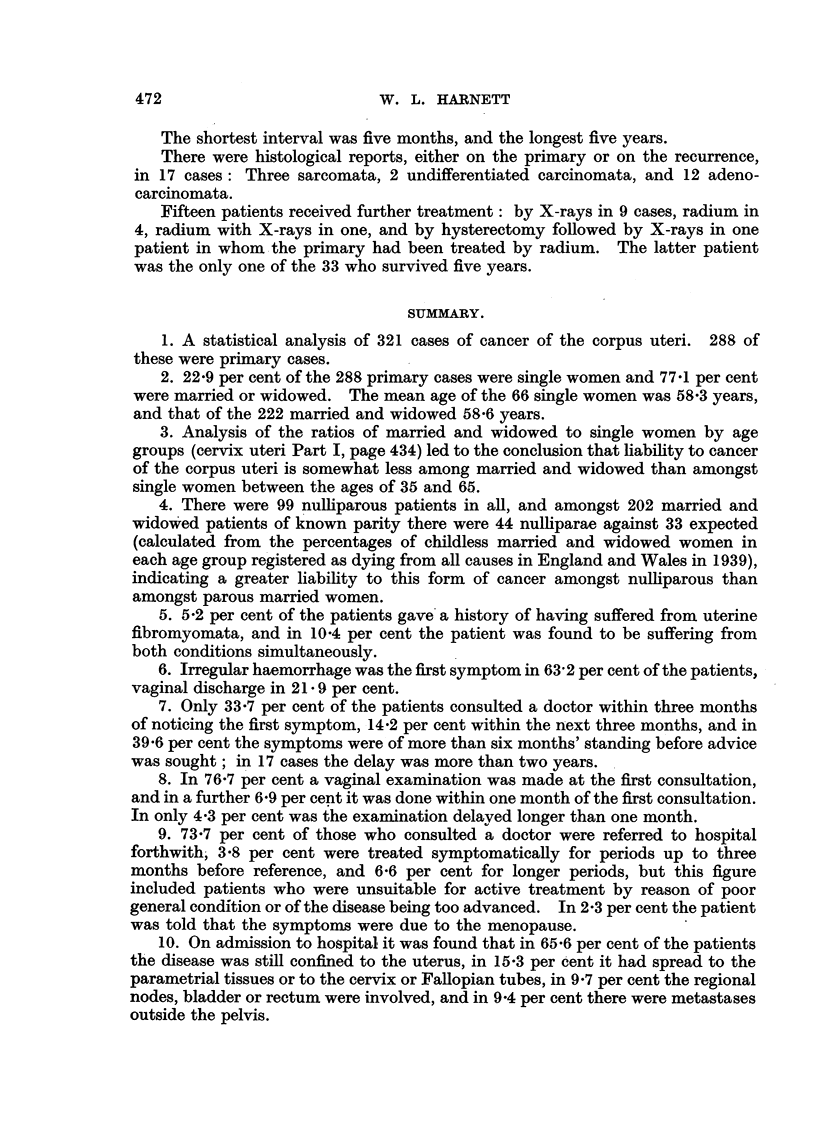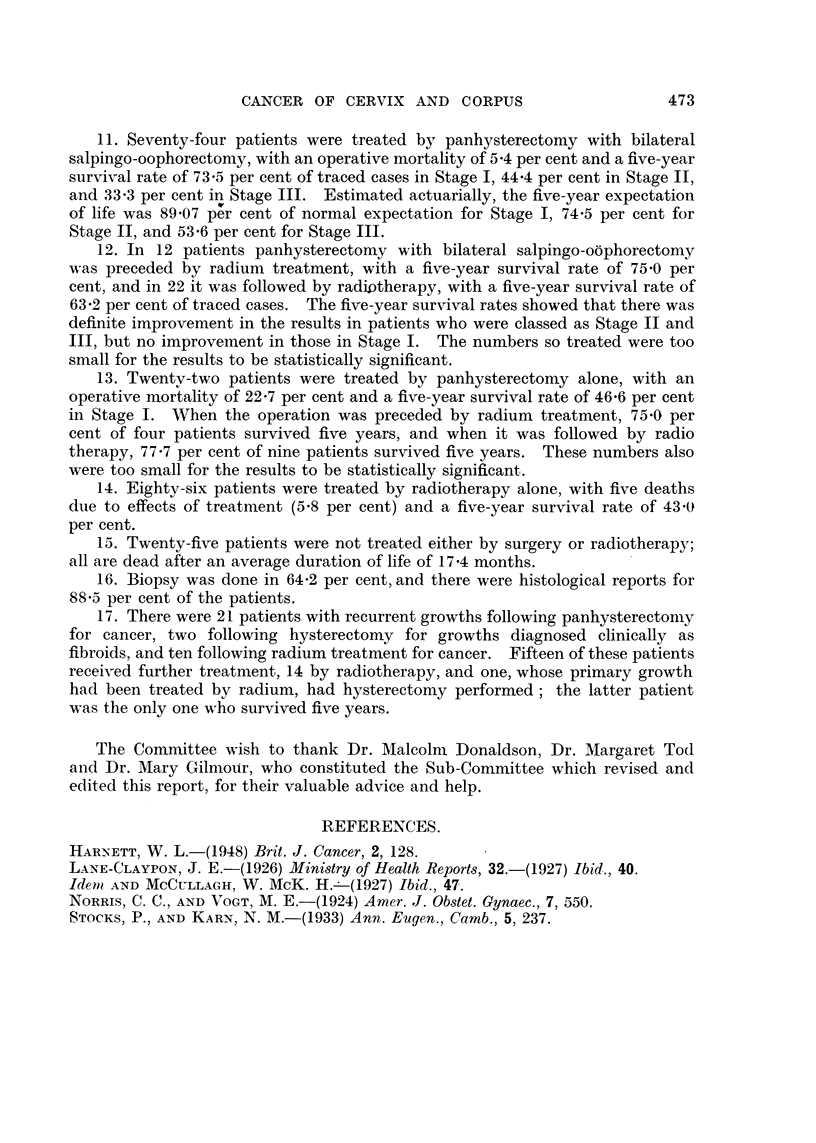# A Statistical Report on 955 Cases of Cancer of the Cervix Uteri and 321 Cases of Cancer of the Corpus Uteri

**DOI:** 10.1038/bjc.1949.49

**Published:** 1949-12

**Authors:** W. L. Harnett


BRITISH JOURNAL OF CANCER

VOL. III | DECEMBER, 1949 | NO. 4

### A STATISTICAL REPORT ON 955 CASES OF CANCER OF THE CERVIX UTERI AND 321 CASES OF CANCER OF THE CORPUS UTERI.

W. L. HARNETT.

*Published for the Clinical Cancer Research Committee of the British Empire Cancer Campaign.*

Received for publication August 31, 1949.

IN 1938–39 the Clinical Cancer Research Committee of the British Empire Cancer Campaign carried out a clinical survey of all cases of cancer seen in the hospitals, both Voluntary and L.C.C., in the Administrative County of London. This was done by means of questionnaires, one of which was filled in by the Registrars in the various hospitals for each patient and returned to the Clinical Cancer Research Committee for record. The work was interrupted by the outbreak of war after it had been in progress for 17 months. By this time 15,203 cases of cancer had been registered, of which 955 were cases of cancer of the cervix uteri and 321 of the corpus uteri. These patients have now been followed up for five years or more and the records analysed.

### PART I: CANCER OF THE CERVIX UTERI.

There were 859 primary and 96 recurrent cases.

### PRIMARY CASES.

*Civil State.*

| | No. | | Per cent. |
|---|---|---|---|
| Single . . . . . | 37 | . | 4·3 |
| Married . . . . | 644 | 819 | 95·3 |
| Widowed . . . . | 175 | | |
| Not stated . . . . | 3 | . | 0·4 |

Lane-Claypon and McCullagh (1927) give the civil states of 815 women suffering from cancer of the cervix as 3·2 per cent single and 96·8 per cent married or widowed.

*Age Distribution.*

| Age groups. | Single. | Married and widowed. | Not stated. | Total. |
|---|---|---|---|---|
| 25–34 | 4 | 22 | — | 26 |
| 35–39 | 1 | 45 | 1 | 47 |
| 40–44 | 8 | 85 | — | 93 |
| 45–49 | 4 | 129 | — | 133 |
| 50–54 | 5 | 147 | — | 152 |
| 55–59 | 8 | 145 | — | 153 |
| 60–64 | — | 98 | 1 | 99 |
| 65–69 | 2 | 75 | — | 77 |
| 70–74 | 4 | 47 | — | 51 |
| 75–79 | 1 | 19 | — | 20 |
| 80– | — | 7 | 1 | 8 |
| Mean age | $51 \cdot 5 \pm 2 \cdot 09$ | $54 \cdot 7 \pm 0 \cdot 37$ | — | $54 \cdot 6 \pm 0 \cdot 37$ |
| Standard deviation | $12 \cdot 7 \pm 1 \cdot 48$ | $10 \cdot 7 \pm 0 \cdot 26$ | — | $10 \cdot 9 \pm 0 \cdot 26$ |

This table, together with the corresponding one for cases of the corpus uteri (p. 454), was submitted to Dr. Percy Stocks, Medical Statistical Officer, General Register Office, for his opinion on the statistical significance of the figures. He comments as follows :

" The incidence of cancer of the uterine cervix and corpus uteri according to civil condition and age can best be studied by comparing the ratios of married and widowed to single women amongst the patients suffering from each form of cancer with that to be expected amongst living women on the one hand and amongst women dying of all causes on the other, at separate age groups.

" *Ratios of Married and Widowed to Single Women.*

| Age group. | Cancer of cervix cases 1938–9. | Cancer of corpus uteri cases 1938–9. | Living women (estimated). | All women dying in 1938. |
|---|---|---|---|---|
| 25– | 5·5 | Indeterminate | 2·4 | 2·0 |
| 35– | 14·4 | 2·0 | 4·1 | 3·6 |
| 45– | 30·7 | 3·2 | 4·8 | 4·6 |
| 55– | 30·4 | 3·3 | 5·2 | 5·6 |
| 65– | 21·1 | 4·0 | 4·9 | 5·6 |

" Provided that it can be assumed that the hospital sample contains the same proportions of single, married and widowed women as the general population at each age period (or that the preponderance of one or other group is constant throughout the age scale), the following conclusions can be drawn :

" (1) Liability to cancer of the cervix uteri is greater at every age amongst married and widowed than amongst single women, and especially between the ages of 45 and 65, when it is about 7 times as great.

" (2) Liability to cancer of the corpus uteri is somewhat less amongst married and widowed than amongst single women, at any rate between the ages of 35 and 65.

"When the age distributions of the cervix and corpus uteri patients are compared, the percentage distributions with their standard errors are as follows:

| | Single women. | | | Married or widowed. | | |
|---|---|---|---|---|---|---|
| | Cervix. | Corpus. | Difference and standard error. | Cervix. | Corpus. | Difference and standard error. |
| Under 45 . | 35·1±7·8 | 9·1±3·5 | 26·0±8·6 | 18·6±1·4 | 7·7±1·8 | 10·9±2·2 |
| 45–64 . | 45·9±8·2 | 68·2±5·7 | 22·3±10·0 | 63·4±1·7 | 65·4±1·7 | 2·9±3·6 |
| 65 and over | 19·0±6·4 | 22·7±5·15 | 3·7±8·2 | 18·1±1·3 | 27·0±3·0 | 8·9±3·3 |
| All ages . | 100·0 | 100·0 | | 100·1 | 100·0 | |

"Amongst single women the proportion under 45 years of age was significantly greater for cervix than corpus, with a corresponding deficiency at ages 45–65. Amongst married or widowed women the proportion under 45 was again significantly greater for cervix than corpus, but over 65 the proportion with carcinoma of the corpus was significantly greater—27·0 to 18·1."

*Heredity.*

| | No. | Per cent. |
|---|---|---|
| Family history of cancer . . . | 90 | 10·5±1·04 |
| ,, ,, ,, in more than one relative | 8 | 1·1 |
| Family history of cancer of the uterus . | 13 | 1·5 |
| No family history of cancer . . | 543 | 63·2±1·6 |
| Not stated . . . . . . | 226 | 26·3±1·5 |

It was not possible in taking the history to decide whether cancer of the uterus in a relative affected the cervix or the corpus uteri.

The family histories of the 90 patients who gave a history of cancer in the family were examined in more detail, only parents and siblings being included.

| | No. | (1) Per cent of all patients of known family history. | (2) Per cent of all patients. |
|---|---|---|---|
| Father suffered from cancer of any other site | 24 | 3·8±0·77 | 2·8±0·6 |
| Mother suffered from cancer of any other site | 34 | 6·8±1·0 | 5·0±0·7 |
| Mother suffered from cancer of uterus . . | 9 | | |
| Brother(s) suffered from cancer of any other site | 10 | 1·6±0·5 | 1·2±0·4 |
| Sister(s) suffered from cancer of any other site | 18 | 3·5±0·73 | 2·6±0·54 |
| Sister(s) suffered from cancer of cervix uteri . | 4 | | |

The percentages give the high and low values, with the 226 patients for whom no family history was obtained excluded from Column 1 and included in Column 2. There were three instances of both parents having died of cancer among the 631 patients whose family history was known, or 1 in 210. The expectation of this occurring by chance in this series was 1 in 386. Stocks and Karn (1933) found the expected frequency of this event for cancer of all regions to be 1 in 180, the numbers involved being 364 fathers and 373 mothers. There is, therefore, no indication that heredity has any influence on the incidence of this disease. These figures may be compared with the corresponding ones for cancer of the breast (Harnett, 1948), all of which are almost exactly double those for the cervix uteri, with the same percentage of "not stated."

### *Relevant Past History.*

*Menstrual cycle.*

Questions were put as to length of interval between the periods, duration of flow, amount lost, and the occurrence of excessive pain. The questions were answered by 84 to 86 per cent of the patients, and the resulting figures were :

| | | No. | Per cent. |
|---|---|---|---|
| Interval : | Short or irregular | 23 | 2·7 |
| | Normal | 704 | 82·0 |
| | Prolonged, or abnormal but irregular | 6 | 0·7 |
| | Not stated | 126 | 14·7 |
| Duration : | Short, up to 3 days | 132 | 15·4 |
| | Normal, 4–6 days | 455 | 53·0 |
| | Prolonged, over 6 days | 144 | 16·8 |
| | Not stated | 128 | 14·9 |
| Amount of flow : | Scanty | 98 | 11·4 |
| | Normal | 485 | 56·5 |
| | Profuse | 131 | 15·3 |
| | Not stated | 145 | 16·9 |
| Pain : | None or normal in amount | 652 | 75·9 |
| | Excessive at all times or before marriage only | 64 | 7·5 |
| Menstrual cycle not stated | | 143 | 16·6 |

The percentage of deviation from normal in the amount of the flow is greater than Lane-Claypon (1926) found in a control series of 509 women, but those for intervals and duration are much the same.

*The menopause.*

The relationship of the onset of the disease to the menopause and the age at which the latter occurred were recorded separately for the single women and for the married and widowed.

| | Single. | Married and widowed. | Not stated. | Total. No. | Total. Per cent. |
|---|---|---|---|---|---|
| Menopause not yet reached | 14 | 277 | 1 | 292 | 34·0 |
| ,, was artificially induced | 2 | 17 | — | 19 | 2·2 |
| Patient was past the menopause | 13 | 479 | 1 | 493 | 57·4 |
| Not stated | 8 | 46 | 1 | 55 | 6·4 |

| *Age at Menopause.* | Single. | Married and widowed | Not stated. | Total. No. | Total. Per cent. |
|---|---|---|---|---|---|
| Under 40 years | 1 | 22 | — | 23 | 4·5 |
| 40– | 3 | 63 | — | 66 | 12·9 |
| 44– | 2 | 148 | — | 150 | 29·3 |
| 48– | 7 | 175 | — | 182 | 35·5 |
| 52– | 2 | 78 | 1 | 81 | 15·8 |
| 56 and over | — | 10 | — | 10 | 2·0 |
| *Interval between the menopause and onset of symptoms.* | | | | | |
| Under 5 years | 5 | 132 | — | 137 | 26·8 |
| 5–10 years | 3 | 97 | 1 | 101 | 19·7 |
| 10 and over | 7 | 267 | — | 274 | 53·5 |
| Total number who were past the menopause | 15 | 496 | 1 | 512 | 100·0 |

The menopause had been artificially induced by sub-total hysterectomy or bilateral oöphorectomy in 15 patients, by radium in 1, and by X-rays in 3 for the treatment of menorrhagia due to fibroids. Lane-Claypon (1926) found that 29 of 509 control patients (5·7 per cent) and 15 of 508 patients with cancer of the breast had had an artificial menopause induced by operation for conditions other than cancer.

| *Children and miscarriages.* | Single. | Married and widowed. | Not stated. | Total. |
|---|---|---|---|---|
| No children | 19 | 71 | 1 | 91 |
| 1 child | 7 | 114 | — | 121 |
| 2 children | 1 | 132 | — | 133 |
| 3 ,, | 2 | 98 | 1 | 101 |
| 4 ,, | — | 91 | — | 91 |
| 5 or more children | — | 281 | — | 281 |
| Not stated | 8 | 32 | 1 | 41 |
| | | | | |
| No miscarriages | 25 | 499 | 2 | 526 |
| 1 miscarriage | 1 | 137 | — | 138 |
| 2 miscarriages | — | 49 | — | 49 |
| 3 or more miscarriages | — | 43 | — | 43 |
| Not stated | 11 | 91 | 1 | 103 |

The 91 nulliparous patients were divided into those who were totally barren and those who had had one or more miscarriages :

| | Single. | Married and widowed. | Not stated. | Total. |
|---|---|---|---|---|
| No children, no miscarriages | 18 | 49 | 1 | 68 |
| No children, but one or more miscarriages | 1 | 15 | — | 16 |
| Not stated | — | 7 | — | 7 |
| Total | 19 | 71 | 1 | 91 |

Of 818 women of known parity (859, less 41 unknown), 91 or 11·1 per cent were nulliparous and 68 or 8·3 per cent were completely sterile. Lane-Claypon (1927) found that 9·3 per cent of 375 women with cancer of the cervix were sterile.

Dr. Stocks comments as follows on these figures when compared with those for corpus uteri (Part II, page 456):

"There were 71 married and widowed women who had not had a child out of 787 of known parity with cancer of the cervix. The expected number, calculated by multiplying the numbers in each age group by the percentages of married and widowed women who were recorded as having had no child at the registration of deaths from all causes in England and Wales in 1939, was 135. There was, therefore, a pronounced deficiency of childless women amongst those suffering from cancer of the cervix. In sharp contrast there were 44 married and widowed women who had not had a child out of 202 of known parity with cancer of the corpus uteri, the expected number by the same method of calculation being only 33."

*Interval between last pregnancy and first symptom of cancer in* 818 *patients of known parity.*

| | |
|---|---|
| No children or miscarriages | 68 |
| Pregnant at the time | 2 |
| Less than 1 year | 5 |
| 1–5 years ago | 26 |
| 5–10 ,, | 51 |
| 10–15 ,, | 108 |
| 15–20 ,, | 95 |
| 20–25 ,, | 126 |
| 25–30 ,, | 87 |
| 30–35 ,, | 60 |
| 35–40 ,, | 39 |
| Over 40 years ago | 37 |
| Not stated | 114 |

The period of maximum incidence was 10–25 years after the birth of the last child. One patient was about 5 months pregnant when the cancer was discovered during examination; she was treated by Caesarian section and Wertheim's hysterectomy, but died from recurrence 18 months later. Another patient passed some pieces of tissue whilst in labour which were found to be carcinomatous; she was treated by radium and X-rays, but died from recurrence 18 months later.

*History of instrumental deliveries.*

| | No. | Per cent. |
|---|---|---|
| No children or miscarriages | 68 | 7·9 |
| Has had one instrumental delivery | 121 | 14·1 |
| ,, more than one instrumental delivery | 36 | 4·2 |
| None | 549 | 63·9 |
| Not stated | 85 | 9·9 |

| *History of previous operations.* | No. | Per cent. |
|---|---|---|
| Cervix operation | 29 | 3·4 |
| Sub-total hysterectomy | 16 | 1·9 |
| Myomectomy | 2 | 0·2 |
| Unilateral oöphorectomy | 19 | 2·2 |
| Bilateral ,, | 4 | 0·5 |
| Mastectomy for cancer | 4 | 0·5 |
| Some other abdominal operation (usually appendicectomy) | 53 | 6·2 |
| None | 675 | 78·6 |
| Not stated | 56 | 6·5 |
| *History of wearing a pessary.* | | |
| Present | 23 | 2·7 |
| No history | 92 | 10·7 |
| Not stated | 744 | 86·6 |
| *History of puerperal lacerations.* | | |
| Perineal or other parturition lacerations | 177 | 20·6 |
| No history | 488 | 56·8 |
| Not stated | 194 | 22·6 |
| *History of pelvic infections.* | | |
| Any pelvic infection | 30 | 3·5 |
| No history | 615 | 71·6 |
| Not stated | 214 | 24·9 |

It is probable that in many cases the blanks in the replies to the questions were meant to be negative answers, but as the figures stand they do not admit of any conclusions being drawn.

*First Symptom.*

| | No. | Per cent. | |
|---|---|---|---|
| Irregular haemorrhage | 457 | 53·2 | 67·5 |
| Sudden profuse haemorrhage | 98 | 11·4 | |
| Bleeding after coitus | 25 | 2·9 | |
| Vaginal discharge | 172 | 20·0 | |
| Pain | 64 | 7·5 | |
| Disorders of micturition | 10 | 1·2 | |
| Symptoms due to secondaries | 7 | 0·8 | |
| Rectal symptoms | 6 | 0·7 | |
| Loss of weight | 4 | 0·5 | |
| No pelvic symptoms ; growth discovered during examination | 3 | 0·3 | |
| Not stated | 13 | 1·5 | |

Symptoms due to secondaries in 7 patients were due to intra-abdominal metastases in 3 patients, to metastases in the brain in 1, in the lung in 1, and in the spinal column and pelvic bones in 2.

*Interval from First Symptom to First Consultation.*

| | No. | Per cent. |
|---|---|---|
| 1 month and under | 214 | |
| 1–2 months | 101 | 45·5 |
| 2–3 ,, | 76 | |
| 3–4 ,, | 66 | 20·7 |
| 4–6 ,, | 112 | |
| 6–9 ,, | 69 | |
| 9–12 ,, | 57 | |
| 12–18 ,, | 41 | 25·6 |
| 18–24 ,, | 26 | |
| 24–48 ,, | 21 | |
| Over 48 months | 6 | |
| Not stated | 70 | 8·1 |

These figures show that there was undue delay in consulting a doctor by more than half the patients, and in 25·6 per cent more than six months elapsed before advice was sought.

*Interval from First Consultation to First Vaginal Examination.*

| | No. | Per cent. |
|---|---|---|
| Done at first consultation | 610 | 71·0 |
| Within 1 month | 79 | 9·2 |
| 1–2 months later | 19 | 3·8 |
| 2–3 ,, ,, | 14 | |
| 3–4 ,, ,, | 8 | |
| 4–5 ,, ,, | 13 | 3·6 |
| 5–6 ,, ,, | 10 | |
| 6–8 ,, ,, | 8 | |
| 8–10 ,, ,, | 2 | 2·1 |
| 10–12 ,, ,, | 8 | |
| Over 12 months later | 5 | 0·6 |
| Not stated | 83 | 9·7 |

*Advice and Treatment before Admission to Hospital.*

| | No. |
|---|---|
| No doctor consulted prior to coming to hospital | 122 |
| Referred to hospital without delay | 583 |
| ,, ,, ,, but delayed going there | 27 |
| Treated symptomatically for periods up to 3 months before reference | 29 |
| Treated symptomatically for periods over 3 months before reference | 57 |
| Reassured or kept under observation | 25 |
| Not stated | 16 |
| Refused treatment | 4 |

The figures in the last two tables show that in 80·2 per cent of patients a vaginal examination was made at, or within one month of the first consultation, and that of the 737 who consulted a doctor, 79·1 per cent were referred to hospital at once. A percentage of 7·7 was kept under symptomatic treatment for more than three months, but this figure included patients who were unsuitable for treatment, either by reason of poor general condition or the disease being too advanced. In 25 instances (3·4 per cent) the patient was not examined *per vaginam* for three or four months and then, when the examination revealed the true nature of the disease, was referred to hospital.

*Symptoms on Admission to Hospital.*

These are shown in order of frequency of occurrence, though most patients complained of more than one symptom.

| | No. | Per cent. |
|---|---|---|
| Vaginal haemorrhage | 777 | 90·5 |
| ,, discharge | 610 | 71·0 |
| Pain | 417 | 48·5 |
| Loss of weight | 334 | 38·9 |
| Disorders of micturition | 119 | 13·9 |
| Anaemia | 64 | 7·5 |
| Rectal symptoms | 11 | 1·3 |
| Symptoms due to secondaries | 8 | 0·9 |
| ,, ,, intestinal obstruction | 1 | 0·1 |

*Findings on Examination.*

The type and extent of the disease as found clinically was recorded in the stages defined in the League of Nations Classification (1937).

| | No. | Per cent. |
|---|---|---|
| Stage I: The carcinoma is strictly confined to the cervix | 201 | 23·4 |
| Stage II: The carcinoma infiltrates the parametrium on one or both sides, but has not invaded the pelvic wall. Upper one-third of vagina infiltrated. Spread to corpus | 318 | 37·0 |
| Stage III: The carcinomatous infiltration of the parametrium has invaded the pelvic wall on one or both sides. Lower one-third of vagina involved. Isolatedmetastases on pelvic wall | 221 | 23·7 |
| Stage IV: Carcinoma involves bladder as determined cystoscopically, or a vesico-vaginal fistula is present. Rectum involved. Distant metastases present | 113 | 13·2 |
| Not staged for lack of data | 6 | 0·7 |
| Totals | 859 | 100·0 |

Cytoscopy—Stage IV cases only.

| | No. | Per cent. |
|---|---|---|
| Done and confirms invasion of bladder by growth | 10 | — |
| ,, does not confirm invasion of bladder by growth | 2 | — |
| Not done | 101 | — |
| **Recto-vaginal and vesico-vaginal fistulae :** | | |
| Recto-vaginal fistula present | 8 | — |
| Vesico-vaginal ,, ,, | 11 | — |
| No fistula present | 76 | — |
| Not stated | 18 | — |
| **Remote metastases—outside pelvis :** | | |
| None found on clinical examination | 693 } | 81·5 |
| ,, after radiological examination | 7 } | |
| Metastases present | 34 | 4·0 |
| Not stated | 125 | 14·6 |
| Sites of metastases in 34 patients—multiple in two : | | |
| Abdominal lymph nodes and peritoneum | 23 | — |
| Liver | 7 | — |
| Skin and subcutaneous tissues | 2 | — |
| Lungs | 1 | — |
| Spine | 1 | — |
| Bones of pelvis | 1 | — |
| Brain | 1 | — |

*Wassermann Reaction.*

The Wassermann reaction was tested in 42 patients, and was found to be positive in 12, negative in 30.

*General Condition by Stages.*

| No. in each stage. | Stage I. 201. | Stage II. 318. | Stage III. 221. | Stage IV. 113. | Not staged. 6. | Total. 859. |
|---|---|---|---|---|---|---|
| Good ; no weight loss | 143 | 176 | 61 | 12 | 1 | 393 |
| Obese, but otherwise good | 6 | 12 | 7 | — | 1 | 26 |
| Fair ; moderate weight loss (up to 2 st.) | 38 | 89 | 94 | 35 | 1 | 257 |
| Poor ; considerable weight loss (over 2 st.) | 9 | 28 | 44 | 44 | 3 | 128 |
| Emaciated | 1 | 4 | 10 | 15 | — | 30 |
| Moribund | — | — | — | 6 | — | 6 |
| Not stated | 4 | 9 | 5 | 1 | — | 19 |
| Per cent in good condition | 74·1 | 59·1 | 30·8 | 10·6 | 33·3 | 48·8 |

Other co-existing diseases: Seven patients were found to be suffering from diabetes, 18 from cardio-vascular disease, 1 from pulmonary tuberculosis, and 6 from mental affection.

### *Clinical Stages.*

On page 441 the cases were grouped in the four clinical stages defined in the League of Nations Classification (1937), and in the following paragraphs the methods of treatment and the results are analysed in the same groups, so that they may be comparable with similar published series of cases. In the cases of patients who were operated on, the operation findings, or in those who died soon after admission, the autopsy findings, were used to correct the staging based on clinical signs alone, with the result that 18 cases previously classified as belonging to Stages I and II were transferred to lower stages.

| | Clinical stages. | Final stages. | Difference. |
|---|---|---|---|
| Stage I | 201 | 191 | — 10 |
| ,, II | 318 | 310 | — 8 |
| ,, III | 221 | 238 | + 17 |
| ,, IV | 113 | 115 | + 2 |
| Not staged | 6 | 5 | — 1 |

### *Methods of Treatment and Five-year Results.*

All deaths from any cause within one month of an operation, whether radical or palliative, were counted as "operation fatalities." In the case of radiotherapeutic treatment, those patients whose death appeared to have been accelerated by the effects of radiotherapy have been classified as " died from the effects of treatment," regardless of the time which had elapsed since treatment was completed.

| *Radical surgical methods.* | No. | Operation fatalities. | Died from effects of treatment. | Survived 5 years. | Died with cancer. | Died without cancer. | Not traced. |
|---|---|---|---|---|---|---|---|
| Wertheim's hysterectomy | 34 | 5 | — | 18 | 9 | 1 | 1 |
| Panhysterectomy with excision of tubes and ovaries | 1 | — | — | — | 1 | — | — |
| Panhysterectomy | 1 | — | — | 1 | — | — | — |
| Vaginal hysterectomy | 1 | — | — | — | — | — | 1 |
| | 37 | 5 | — | 19 | 10 | 1 | 2 |
| *Radical combined methods.* | | | | | | | |
| Wertheim's hysterectomy preceded by radium | 11 | 1 | — | 6 | 3 | 1 | — |
| Wertheim's hysterectomy preceded by H.V. X-rays | 1 | — | — | 1 | — | — | — |
| Wertheim's hysterectomy followed by H.V. X-rays | 11 | — | — | 5 | 6 | — | — |
| Panhysterectomy with excision of tubes and ovaries preceded by radium | 3 | — | — | — | 2 | — | 1 |
| Panhysterectomy preceded by H.V. X-rays | 1 | — | — | 1 | — | — | — |
| Panhysterectomy followed by H.V. X-rays | 9 | — | — | 6 | 3 | — | — |
| | 36 | 1 | — | 19 | 14 | 1 | 1 |

| | No. | Operation fatalities | Died from effects of treatment. | Survived 5 years. | Died with cancer. | Died without cancer. | Not traced. |
|---|---|---|---|---|---|---|---|
| *Palliative combined methods.* | | | | | | | |
| Radium combined with palliative operation (colostomy) | 3 | — | — | — | 3 | — | — |
| Radium after abandoned radical operation | 2 | — | — | 1 | 1 | — | — |
| | 5 | — | — | 1 | 4 | — | — |
| *Radiotherapy alone.* | | | | | | | |
| Radium alone | 313 | 4 | 7 | 97 | 185 | 6 | 14 |
| Radium followed by H.V. X-rays | 261 | — | 4 | 77 | 170 | 5 | 5 |
| H.V. X-rays followed by radium | 57 | — | 1 | 19• | 36 | 1 | — |
| H.V. X-rays alone | 52 | — | 3 | 2 | 44 | 1 | 2 |
| Diathermy excision followed by X-rays and radium | 2 | — | — | — | 2 | — | — |
| Radium preceded by drainage of pyometra | 12 | 1 | — | 3 | 8 | — | — |
| | 697 | 5 | 15 | 198 | 445 | 13 | 21 |
| *Palliative operations and not treated.* | | | | | | | |
| Exploratory laparotomy alone | 8 | 2 | — | — | 6 | — | — |
| Laparotomy for intestinal obstruction | 2 | 2 | — | — | — | — | — |
| Refused the treatment advised | 10 | — | — | 1 | 8 | — | 1 |
| Not treated by surgery or radiotherapy | 64 | — | — | — | 64 | — | — |
| | 84 | 4 | — | 1 | 78 | — | 1 |
| Totals of all cases | 859 | 15 | 15 | 238 | 551 | 15 | 25 |

There were 5 "operation fatalities" following application of radium; 2 of these were due to pulmonary embolism, 1 to femoral thrombosis, 1 to general peritonitis, and 1 to post-anaesthetic pneumonia. Fifteen patients were classified as "died from effects of treatment," 5 were from radio-necrosis, 2 from general peritonitis, 3 from local peritonitis, 2 from sepsis, and 1 each from pulmonary complications, femoral thrombosis and radiation sickness. In the case of one patient who refused treatment but survived five years, the diagnosis was considered to have been mistaken, as subsequent examination revealed no clinical evidence of malignancy.

*Duration of Symptoms at Time of Commencing Treatment.*

| | No. | Survived. | Per cent. |
|---|---|---|---|
| 1 month and under | 80 | 18 | |
| 1–3 months | 166 | 64 | 30·9 (first three rows) |
| 3–6 ,, | 213 | 60 | |
| 6–12 ,, | 158 | 56 | 30·1 (next two rows) |
| Over 12 months | 134 | 32 | |
| Not known | 24 | 7 | — |
| | 775 | 237 | |

The estimates of the duration of symptoms, like those given on page 440, are based on the patients' statements. It will be seen that there is no significant difference between the survival rates of those who came under treatment within the first six months of noticing symptoms and those in whose cases the delay was longer. The former constitute 59·3 per cent of the treated patients, and the latter 37·6 per cent. This must not be taken to mean that there is no difference between the survival rates of patients in whom the disease was diagnosed and treated in the early stages and those who were in the later stages when they came under treatment.

*Operation Findings.*

Forty-six patients who were classified clinically as Stage I underwent laparotomy, 8 of whom were found to have metastases in the pelvic lymph nodes, which placed them in Stage III, 1 had metastases in the paraortic lymph nodes, and 1 had involvement of the bladder, so they belonged to Stage IV. Of 24 patients classified clinically as Stage II and operated on, 8 were found to have metastases in the pelvic lymph nodes and so to be in Stage III.

*Five-year Follow-up Results for all Cases.*

| | First year. | Second year. | Third year. | Fourth year. | Fifth year. | Totals. | | |
|---|---|---|---|---|---|---|---|---|
| Operation fatalities | 15 | — | — | — | — | 15 | 596 | 859 |
| Died from effects of treatment | 14 | 1 | — | — | — | 15 | | |
| Died with cancer | 292 | 136 | 73 | 35 | 15 | 551 | | |
| „ without cancer | 3 | 2 | 5 | 2 | 3 | 15 | | |
| Alive and well | .. | .. | .. | .. | 150 | .. | 238 | |
| „ with cancer | .. | .. | .. | .. | 25 | | | |
| „ state unknown | .. | .. | .. | .. | 63 | | | |
| Untraced | .. | .. | .. | .. | 25 | .. | 25 | |
| Total died each year | 324 | 139 | 78 | 37 | 18 | | | |
| Percentage of all cases | 37·7 | 16·2 | 9·1 | 4·3 | 2·1 | | | |

The 5-year survival rate for all cases was 27·7 per cent. Of the 238 survivors, 6 are known to have died of cancer in the sixth year, 1 in the seventh, 2 in the eighth, and 1 in the tenth year. Two patients who had been successfully treated by radium died of other causes in the sixth and seventh years respectively.

*Analysis of Five-year Results by Methods of Treatment and Stages.*

| *Radical surgical methods.* | Stage I. | Stage II. | Stage III. | Stage IV. | Not staged. | Total. |
|---|---|---|---|---|---|---|
| Wertheim's hysterectomy | 22 | 12 | — | — | — | 34 |
| Known survivors | 14 | 4 | — | — | — | 18 |
| Per cent survived | 63·3 | 33·3 | — | — | — | 53·0 |
| Per cent of traced cases | 66·6 | 33·3 | — | — | — | 54·5 |
| Panhysterectomy, abdominal or vaginal | 2 | 1 | — | — | — | 3 |
| Known survivors | 1 | — | — | — | — | 1 |
| Per cent survived | 50·0 | 0·0 | — | — | — | 33·3 |
| Per cent of traced cases | 100·0 | 0·0 | — | — | — | 50·0 |
| Total of radical surgical methods | 24 | 13 | — | — | — | 37 |
| Known survivors | 15 | 4 | — | — | — | 19 |
| Per cent survived | 62·5 | 30·8 | — | — | — | 51·4 |
| Per cent of traced cases | 68·1 | 30·8 | — | — | — | 54·3 |
| *Radical combined methods.* | | | | | | |
| Wertheim's hysterectomy preceded by radium | 7 | 4 | — | — | — | 11 |
| Survivors | 5 | 1 | — | — | — | 6 |
| Per cent survived | 71·4 | 25·0 | — | — | — | 54·5 |
| Wertheim's hysterectomy preceded or followed by H.V. X-rays | 5 | 5 | 1 | — | 1 | 12 |
| Survivors | 4 | 1 | — | — | 1 | 6 |
| Per cent survived | 80·0 | 20·0 | 0·0 | — | 100·0 | 50·0 |

| *Radical combined methods*—Cont. | Stage I. | Stage II. | Stage III. | Stage IV. | Not staged. | Total. |
|---|---|---|---|---|---|---|
| Panhysterectomy preceded by radium | 1 | 2 | — | — | — | 3 |
| Known survivors | — | — | — | — | — | — |
| Per cent survived | 0·0 | 0·0 | — | — | — | 0·0 |
| Panhysterectomy preceded or followed by H.V. X-rays | 9 | — | — | 1 | — | 10 |
| Survivors | 7 | — | — | — | — | 7 |
| Per cent survived | 77·7 | — | — | 0·0 | — | 70·0 |
| Total of radical combined methods | 22 | 11 | 1 | 1 | 1 | 36 |
| Known survivors | 16 | 2 | — | — | 1 | 19 |
| Per cent survived | 72·7 | 18·2 | 0·0 | 0·0 | 100·0 | 52·8 |
| Per cent of traced cases | 72·7 | 20·0 | 0·0 | 0·0 | 100·0 | 54·3 |
| *Radiotherapy alone.* | | | | | | |
| Radium alone | 87 | 134 | 76 | 16 | — | 313 |
| Known survivors | 45 | 45 | 7 | — | — | 97 |
| Per cent survived | 51·7 | 33·6 | 9·2 | 0·0 | — | 31·0 |
| Per cent of traced cases | 55·5 | 34·9 | 9·5 | 0·0 | — | 32·4 |
| Radium followed by H.V. X-rays | 43 | 126 | 75 | 15 | 2 | 261 |
| Known survivors | 20 | 37 | 17 | 2 | 1 | 77 |
| Per cent survived | 46·5 | 29·4 | 22·7 | 13·3 | 50·0 | 29·5 |
| Per cent of traced cases | 47·6 | 30·1 | 23·0 | 13·3 | 50·0 | 30·1 |
| H.V. X-rays followed by radium | 13 | 15 | 20 | 8 | 1 | 57 |
| Survivors | 5 | 6 | 5 | 2 | 1 | 19 |
| Per cent survived | 38·5 | 40·0 | 25·0 | 25·0 | 100·0 | 33·3 |
| H.V. X-rays alone | 3 | 6 | 23 | 20 | — | 52 |
| Known survivors | 1 | — | 1 | — | — | 2 |
| Per cent survived | 33·3 | 0·0 | 4·3 | 0·0 | — | 3·8 |
| Per cent of traced cases | 33·3 | 0·0 | 4·5 | 0·0 | — | 4·0 |
| Total of radiotherapeutic methods | 149 | 288 | 198 | 59 | 3 | 697 |
| Known survivors | 72 | 89 | 31 | 4 | 2 | 198 |
| Per cent survived | 48·3 | 30·9 | 15·7 | 6·8 | 66·6 | 28·4 |
| Per cent of traced cases | 50·7 | 31·8 | 16·0 | 7·3 | 66·6 | 29·3 |
| Total of all cases, treated and not treated | 201 | 318 | 221 | 113 | 6 | 859 |
| Known survivors | 104 | 95 | 31 | 5 | 3 | 238 |
| Per cent survived | 51·7 | 29·9 | 14·0 | 4·4 | 50·0 | 27·7 |
| Per cent of traced cases | 54·2 | 30·7 | 14·3 | 4·5 | 50·0 | 28·5 |

### *Analysis of Five-year Results by Ages in Relation to Methods of Treatment. Stages I and II only.*

| Age group. | Wertheim's hysterectomy with or without radiotherapy. | | | Radium alone. | | |
|---|---|---|---|---|---|---|
| | Total No. | Known survivors. | Per cent. | Total No. | Known survivors. | Per cent. |
| 25– | 4 | 2 | — | 6 | 3 | — |
| 35– | 16 | 10 | — | 40 | 20 | — |
| 45– | 17 | 6 | — | 71 | 26 | — |
| 55– | 17 | 10 | — | 65 | 25 | — |
| 65– | 1 | 1 | — | 33 | 14 | — |
| 75– | — | — | — | 6 | 2 | — |
| | 55 | 29 | 52·7 | 221 | 90 | 40·7 |
| | $\chi^2 = 4{\cdot}00$ <br> $P < 0{\cdot}50 > 0{\cdot}30$ | | | $\chi^2 = 2{\cdot}43$ <br> $P < 0{\cdot}80 > 0{\cdot}70$ | | |

These figures show that there are no statistically significant variations in the survival rates of the different age-groups. Similar calculations for the two stages separately gave the same results.

*Estimation of Survival after Treatment.*

Dr. Stocks, to whom this question was referred, advised that unless the follow-up of cases makes it possible to assign accurately every death, either to cancer on the one hand, or to intercurrent causes on the other, the only sound method of dealing with the duration of survival is an actuarial one, which means calculating from a life-table the total months which would be lived in the period of observation by a group of people in the general population having the same sex-age distribution as the group of patients dealt with. This gives the mean number of months expected to be lived during the five years by each group. The mean number of months actually lived is then calculated and expressed as a percentage of the normal expectation for that group, making allowances for cases followed up for less than five years.

English Life Table No. 10 (1930–32) was used for ascertaining the expectation of life.

*Radical surgery alone.*

| | | |
|---|---|---|
| Wertheim's hysterectomy | 34 cases | 37 |
| Panhysterectomy | 3 ,, | |

| | | Stage I. | Stage II. |
|---|---|---|---|
| Number of cases of known duration | | 23 | 13 |
| Mean number of months lived in 5 years from onset | Maximum possible | 59·48 | 60·00 |
| | Expected | 57·76 | 57·91 |
| | Actual | 50·35 | 34·31 |
| | Per cent of Expected | 87·17 | 59·25 |

*Radical surgery combined with radiotherapy.*

| | | |
|---|---|---|
| Wertheim's hysterectomy | 23 cases | 36 |
| Panhysterectomy | 13 ,, | |

| | | Stage I. | Stage II. |
|---|---|---|---|
| Number of cases of known duration | | 22 | 10 |
| Mean number of months lived in 5 years from onset | Maximum possible | 60·00 | 60·00 |
| | Expected | 58·46 | 58·74 |
| | Actual | 51·41 | 30·10 |
| | Per cent of Expected | 87·94 | 51·24 |

*Radium alone.*

| | | Stage I. | Stage II. | Stage III. |
|---|---|---|---|---|
| Number of cases of known duration | | 79 | 126 | 69 |
| Mean number of months lived in 5 years from onset | Maximum possible | 59·85 | 60·00 | 59·65 |
| | Expected | 56·83 | 57·06 | 56·21 |
| | Actual | 48·71 | 39·10 | 24·14 |
| | Per cent of Expected | 85·71 | 68·52 | 42·95 |

*Radium followed by X-rays.*

| | | Stage I. | Stage II. | Stage III. |
|---|---|---|---|---|
| Number of cases of known duration | | 40 | 122 | 73 |
| Mean number of months lived in 5 years from onset | Maximum possible | 60·00 | 59·90 | 59.84 |
| | Expected | 57·36 | 57·08 | 56·86 |
| | Actual | 45·65 | 37·84 | 32·22 |
| Per cent of Expected | | 79·59 | 66·29 | 56·67 |

*H.V. X-rays followed by radium.*

| | | Stage I. | Stage II. | Stage III. |
|---|---|---|---|---|
| Number of cases of known duration | | 13 | 14 | 20 |
| Mean number of months lived in 5 years from onset | Maximum possible | 60·00 | 60·00 | 60·00 |
| | Expected | 58·29 | 57·87 | 58.12 |
| | Actual | 36·85 | 42·14 | 30·25 |
| Per cent of Expected | | 63·22 | 72·82 | 52·05 |

*X-rays alone.*

| | | Stage I. | Stage II. | Stage III. |
|---|---|---|---|---|
| Number of cases of known duration | | 3 | 5 | 22 |
| Mean number of months lived in 5 years from onset | Maximum possible | 60·00 | 60·00 | 60·00 |
| | Expected | 57·84 | 54·64 | 56·04 |
| | Actual | 39·33 | 20·80 | 19·86 |
| Per cent of Expected | | 68·00 | 38·07 | 35·44 |

*Not treated by surgery or radiotherapy.*

| | | All stages. |
|---|---|---|
| Number of cases of known duration | | 64 |
| Mean number of months lived in 5 years from onset | Maximum possible | 60·00 |
| | Expected | 55·44 |
| | Actual | 14·36 |
| Per cent of Expected | | 25·90 |

These figures show that in Stage I hysterectomy gave the best results, which slightly improved if operation was preceded by radium treatment; the results with radium alone were almost as good as those of operation, and were not improved by courses of X-rays in addition. In Stage II both the 5-year survival rate and the expectation of life were better after radium treatment than after surgery, and in one small group a preliminary course of X-rays slightly improved the results. In Stage III almost all patients were treated by radium, and it was found that a course of X-rays, either before or after the radium treatment, gave improved 5-year survival rate and expectation of life.

*Biopsy.*

| | No. | Per cent. |
|---|---|---|
| Biopsy done before or at commencement of treatment | 629 | 79·6 |
| ,, ,, during radiation treatment | 55 | |
| Not done | 175 | 20·4 |

*Pathological Report.*

| | No. | Per cent. |
|---|---|---|
| No histological examination before or after death | 141 | 16·4 |
| Histological examination done before or after death | 718 | 83·6 |
| Result doubtful | 3 | |
| The specimen examined was non-malignant | 13 | |
| Squamous cell carcinoma, keratinizing | 541 | |
| ,, ,, ,, non-keratinizing | 29 | |
| ,, ,, ,, undifferentiated | 37 | |
| Carcinoma, type unspecified | 23 | |
| Spheroidal cell carcinoma | 10 | |
| Adenocarcinoma | 44 | |
| ,, colloid | 1 | |
| ,, papillary | 10 | |
| Transitional cell carcinoma | 2 | |
| Basal cell carcinoma | 3 | |
| Chorion-carcinoma | 1 | |
| Spindle cell sarcoma | 1 | |

*Basis of diagnosis in* 859 *cases.*

*Clinically malignant:*

| | No. | Per cent. |
|---|---|---|
| Confirmed by histological examination and/ or autopsy | 781 | 90·9 |
| ,, by appearance of metastases or recurrence | 11 | 1·3 |
| Diagnosis based on clinical evidence only | 66 | 7·7 |

*Clinically benign:*

| | No. | Per cent. |
|---|---|---|
| Proved malignant by appearance of recurrence | 1 | 0·1 |

*Carcinoma of Cervix following Supravaginal Hysterectomy.*

There were 16 patients (1·9 per cent) who had undergone supravaginal hysterectomy, usually for the treatment of fibroids 1–18 years previously, mean interval 9·5 years. The mean age was 53 years. There were 3 nulliparae, one 1-para, and the remainder were multiparae. Five patients were classified as in Stage I, 5 in Stage II, 5 in Stage III, and one in Stage IV. Two patients were treated by radical surgery with one operation fatality and one survival; 5 by radium alone, of whom 2 have survived 5 years, but one of these died of cancer in the eighth year; 9 by radium with X-rays, of whom 3 have survived 5 years. The diagnosis of carcinoma was confirmed by biopsy in 15 patients, in 12 of whom the specimen was reported as keratinizing squamous cell carcinoma, in one as undifferentiated squamous cell carcinoma, in one as columnar cell carcinoma, and in one as papillary adenocarcinoma, the latter being the patient who died of recurrence in the eighth year.

30

*Other Primary Growths.*

*Previous primary tumours*—3 patients.

1. Age 58. Simple mastectomy for histologically confirmed spheroidal cell carcinoma of breast, two years previously. Admitted with metastases in vertebrae and a Stage I growth in the cervix which proved to be a papillary adenocarcinoma. Treated by X-rays. Known to be alive at the end of the fifth year.

2. Age 64. Had had partial cystectomy for cancer of the bladder (histology not recorded) 5 years previously; no recurrence. Admitted with Stage II prickle-cell carcinoma of cervix. Radium treatment. Died in first year.

3. Age 69. Had oöphorectomy for papillary adenocarcinoma 4 years previously; no recurrence. Admitted with a Stage II carcinoma of cervix of same histology. Radium and H.V. X-ray treatment. Died in the fourth year.

*Simultaneous primary growths*—4 patients.

4. Age 50. Stage II carcinoma of cervix and rodent ulcer of forehead. No histology. H.V. X-rays to cervix; refused to continue treatment. Died in second year.

5. Age 58. Stage II squamous cell carcinoma of cervix. Treated by radium and H.V. X-rays; no recurrence. Simultaneous spheroidal cell carcinoma of breast, Stage III. Treated by H.V. X-rays and radium. Died in third year from metastases of the breast growth.

6. Age 65. Carcinoma of cervix, Stage IV, no histology. Treated by radium. Also rodent ulcer of right eyelid, which recurred after excision and was histologically carcinoma. Metastases in cervical nodes led to death in third year.

7. Age 65. Carcinoma of cervix, Stage III. No treatment. A separate cauliflower growth found in rectum at autopsy. No histology of either growth.

*Subsequent primary growths*—5 patients.

8. Age 55. Squamous cell carcinoma of cervix, Stage II. Radium and H.V. X-ray treatment. Local recurrence in third year. A carcinoma of the right breast was found in the third year and treated by simple mastectomy. Alive with cancer at the end of the fifth year.

9. Age 50. Carcinoma of cervix, Stage II; histologically not malignant. Treated by radium and H.V. X-rays. No recurrence. Carcinoma of breast in the seventh year (no histology), treated by radon needling. Alive with cancer at the end of the seventh year.

10. Age 46. Squamous cell carcinoma of cervix, Stage I. Treated by radium, no recurrence. Died in the third year from an adenocarcinoma of the bronchus.

11. Age 58. Squamous cell carcinoma of cervix, Stage II. Treated by radium and H.V. X-rays. Generalized recurrence in the third year. Clinically and radiologically there was also a carcinoma of oesophagus; no biopsy. Died in the third year.

12. Age 64. Squamous cell carcinoma of cervix, Stage II. Treated by radium and H.V. X-rays. Carcinoma of breast in the seventh year, spheroidal cell carcinoma, with involvement of nodes. Died two and a half years later from recurrence of the growth in the cervix.

*Cause of Death in* 596 *Patients.*

| | No. |
|---|---|
| Cachexia | 428 |
| Uraemia | 34 |
| Pulmonary complications | 32 |
| Haemorrhage | 17 |
| Peritonitis | 14 |
| Cardio-vascular disease | 13 |
| Sepsis | 11 |
| Embolism (pulmonary 5, cerebral 1, local 1) | 7 |
| Intestinal obstruction | 6 |
| Cerebral | 4 |
| Surgical shock | 3 |
| Intercurrent disease or unknown cause | 27 |

*Autopsy.*

| | No. | Per cent. |
|---|---|---|
| No autopsy | 514 | 86·3 |
| Autopsy done | 82 | 13·8 |

*Relevant autopsy findings* (*multiple in some cases*).

| | No. |
|---|---|
| Local growth only | 11 |
| Extension to neighbouring parts | 47 |
| Metastases in pelvic lymph nodes | 12 |
| ,, abdominal lymph nodes | 22 |
| ,, liver and abdominal organs | 20 |
| ,, lungs and pleurae | 8 |
| ,, brain | 3 |
| ,, skeletal system | 6 |
| Pulmonary complications | 19 |
| Abdominal ,, | 16 |
| No growth found (operated cases) | 4 |

RECURRENT CASES.

There were 96 recurrent cases, of which 13 followed Wertheim's hysterectomy, and 83 treatment by radium, with or without H.V. X-rays. The relation of the type of recurrence to the method of treatment of the primary with the mean intervals of freedom are shown below.

| *Following Wertheim's hysterectomy*—13 cases. | No. | Mean interval of freedom in months. |
|---|---|---|
| Local recurrence only | 7 | 14·7 |
| Recurrence deep in pelvis | 3 | 70·0 |
| Both local and pelvic | 2 | 6·0 |
| Distant metastases | 2 | 8·0 |

In one patient there were both distant and pelvic metastases. The shortest interval was 3 months and the longest 144 months.

| *Following radium treatment*—83 cases. | No. | Mean interval of freedom in months. |
|---|---|---|
| Local recurrence | 13 | 18·0 |
| Recurrence deep in pelvis | 25 | 25·4 |
| Both local and pelvic | 38 | 24·4 |
| Distant metastases | 13 | 23·2 |

In 6 patients there were both distant and pelvic metastases. The shortest interval was 2 months, and the longest 144 months.

Six of the 13 patients with recurrences following hysterectomy received further treatment: with radium, one patient; radium and H.V. X-rays, 2; X-rays alone, 3; colostomy was performed for the relief of intestinal obstruction in 2, and 5 were not treated. All 13 patients are dead.

Thirty of the 83 patients with recurrences following radium treatment received further treatment. Three with radium alone for local recurrence; 4 with radium and H.V. X-rays; 20 with H.V. X-rays alone; 2 with H.V. X-rays following colostomy; one by colostomy alone, and 53 were not treated. One patient is still alive, 81 are dead and 1 is untraced.

SUMMARY.

1. A statistical analysis of 955 cases of cancer of the cervix uteri. 859 of these were primary cases.

2. 4·3 per cent of the patients were single women and 95·3 per cent were married or widowed. The mean age of the single women was 51·5 years, and that of the married and widowed 54·7.

3. Analysis of the ratios of married and widowed to single women by age groups led to the conclusion that liability to cancer of the cervix uteri was greater at every age amongst married and widowed than amongst single women, and that between the ages of 45 and 65 it was about seven times as great.

4. There were 91 nulliparous patients in all, and amongst 787 married and widowed patients of known parity there were 71 nulliparae against 135 expected (calculated from the percentages of childless married and widowed women in each age group registered as dying from all causes in England and Wales in 1939), indicating that parous married women were more liable to this form of cancer than nulliparous women.

5. Irregular haemorrhage was the first symptom in 67·5 per cent of the patients, vaginal discharge in 20 per cent.

6. 45·5 per cent of the patients consulted a doctor within three months of noticing the first symptoms, 20·7 per cent within the next three months, and in 25·6 per cent the symptoms were of more than six months' duration before advice was sought. In 27 cases the delay was more than two years.

7. In 71 per cent a vaginal examination was made at the first consultation, and in a further 9·2 per cent it was done within one month of the first consultation. In only 10·1 per cent was the examination delayed longer than one month.

8. 79·1 per cent of the patients who consulted a doctor were referred to hospital forthwith, 3·9 per cent were treated symptomatically for periods up to three months before reference, and 7·7 per cent were kept under treatment for more than three months, but this figure included patients who were unsuitable for active treatment by reason of poor general condition or the disease being too advanced. In 3·4 per cent the patient was told that there was nothing serious the matter until, some months later, examination revealed the true nature of the disease.

9. On admission to hospital the cases were classified according to the League of Nations Classification (1937) into four stages, Stage I 23·4 per cent, Stage II 37·0, Stage III 25·7, Stage IV 13·2, and cases not staged for lack of data 0·7 per cent,

10. Thirty-four patients in Stages I and II were treated by Wertheim's hysterectomy with an operation mortality of 14·7 per cent, and a 5-year survival rate of 66·6 per cent of traced cases in Stage I and 33·3 per cent in Stage II. Estimated actuarially, the 5-year expectation of life for radical operations was 87·17 and 59·25 per cent of normal expectation respectively.

11. Thirty-six patients were treated by Wertheim's hysterectomy or panhysterectomy preceded or followed by radiotherapy, with an operative mortality of 2·8 per cent. The 5-year survival rate averaged 72·7 per cent of traced cases in Stage I and 20 per cent in Stage II. Estimated actuarially the 5-year expectation of life was 87·9 per cent of normal expectation in Stage I and 51·2 per cent in Stage II.

12. Six hundred and ninety-seven patients were treated by radiotherapy alone with 5 operation fatalities following radium applications, and 15 in whom death was due to the effects of treatment—radio-necrosis, peritonitis, pulmonary complications, etc.—making 2·9 per cent.

Three hundred and thirteen patients were treated by radium alone with a 5-year survival rate of 55·5 per cent of traced cases in Stage I, and 34·9 per cent of traced cases in Stage II. Actuarially estimated, the 5-year expectation of life was 85·7 per cent of normal expectation in Stage I and 68·5 per cent in Stage II. For all methods of radiotherapy the average 5-year survival rates were 50·7 per cent of traced cases in Stage I, 31·8 per cent in Stage II, 16 per cent in Stage III, and 7·3 per cent in Stage IV.

13. Sixty-four patients were not treated either by surgery or by radiotherapy; all are dead after an average duration of life of 14·36 months, which is 25·90 per cent of the normal 5-year expectation of life.

14. Biopsy was done in 79·6 per cent of the patients and there were histological reports for 83·6 per cent.

15. In 16 patients the growth arose in the stump of the cervix remaining after supravaginal hysterectomy, performed on an average 9·5 years previously. Two patients were treated by radical surgery of whom 1 survived, and 14 by radiotherapy of whom 5 survived five years.

16. There were 13 patients with recurrent growths following Wertheim's hysterectomy, and 83 following radiotherapy.

### PART II: CANCER OF THE CORPUS UTERI.

There were 288 primary and 33 recurrent cases.

### Primary Cases.

*Civil State.*

| | No. | | Per cent. |
|---|---|---|---|
| Single | 66 | | 22·9 |
| Married | 151 | 222 | 77·1 |
| Widowed | 71 | | |

Lane-Claypon and McCullagh (1927) give the civil state of 207 women suffering from cancer of the corpus uteri as 22·7 per cent single and 77·3 per cent married or widowed ; the percentage of single women amongst those suffering from cancer of the cervix was 3·2 and the difference of 19·5±2·01 was statistically significant. Our figures of 22·9 per cent and 4·3 per cent (Part I) also show a statistically significant difference of 18·6±2·57.

*Age Distribution.*

| Age groups. | Single. | Married and widowed. | Total. |
|---|---|---|---|
| 25–34 | — | 5 | 5 |
| 35–39 | 2 | 4 | 6 |
| 40–44 | 4 | 8 | 12 |
| 45–49 | 4 | 25 | 29 |
| 50–54 | 15 | 35 | 50 |
| 55–59 | 16 | 41 | 57 |
| 60–64 | 10 | 44 | 54 |
| 65–69 | 5 | 28 | 33 |
| 70–74 | 6 | 24 | 30 |
| 75– | 4 | 8 | 12 |
| Mean age | 58·3±1·17 | 58·6±0·68 | 58·5±0·59 |
| Standard deviation | 9·5±0·83 | 10·2±0·48 | 10±0·42 |

The higher mean age as compared with that of patients with cancer of the cervix is in accord with general experience. The ratios of married and widowed to single women by age groups compared with the corresponding ratios for cancer of the cervix, with Dr. Stocks' comments, have already been given in Part I, page 434.

*Heredity.*

| | No. | Per cent. |
|---|---|---|
| Family history of cancer | 35 | 12·2±1·9 |
| ,, ,, ,, in more than one relative | 9 | 3·1 |
| ,, ,, ,, of the uterus | 3 | 1·0 |
| No family history of cancer | 154 | 53·5±2·9 |
| Not stated | 99 | 34·3±2·8 |

The family histories of the 35 patients who gave a history of cancer in the family were examined in more detail, only parents and siblings being included.

| | No. | (1) Per cent of all patients of known family history. | (2) Per cent of all patients. |
|---|---|---|---|
| Father suffered from cancer of any other site | 9 | 4·8±1·55 | 3·1±1·02 |
| Mother suffered from cancer of any other site | 11 } | 7·4±1·9 | 4·9±1·27 |
| Mother suffered from cancer of uterus | 3 } | | |
| Brother(s) suffered from cancer of any other site | 7 | 3·7±1·37 | 2·4±0·9 |
| Sister(s) suffered from cancer of any other site | 14 | 7·4±1·9 | 4·9±1·27 |
| Sister(s) suffered from cancer of uterus | — | — | — |

The percentages give the high and low values with the 99 patients for whom no family history was obtained excluded from Column 1 and included in Column 2. There was only one instance of both parents having died of cancer among the 189 patients whose family history was known, which is about the same proportion as the expected frequency for all regions of the body (Stocks and Karn, 1933). Although the percentages are slightly higher than those for the cervix uteri, they are not so high as to suggest that heredity is a factor in the aetiology.

### *Relevant Past History.*

*Menstrual cycle.*

Questions were put as to the length of the cycle, duration and amount of flow and presence of any dysmenorrhoea. The questions were answered by 74·0 per cent of the patients. The interval was normal in 67·7 per cent, duration was normal in 43·7 per cent, prolonged in 17·4 per cent. The amount lost was recorded as scanty in 7·3 per cent, normal in 46·2 per cent, and excessive in 17 per cent. Dysmenorrhoea, either premarital only, or at all times was reported by 6·2 per cent. The percentages of deviation from normal are greater than in the case of the cervix uteri, and greater than those recorded for a control series of 509 women (Lane-Claypon, 1926), but the percentages are invalidated by 26 per cent of " not stated."

*The menopause.*

The relationship of the onset of the disease to the menopause and the age at which the latter occurred were recorded separately for the single and for the married and widowed.

| | Single. | Married and widowed. | Total. | |
|---|---|---|---|---|
| | | | No. | Per cent. |
| Menopause not yet reached | 12 | 43 | 55 | 19·1 |
| ,, was artificially induced | 1 | 3 | 4 | 1·4 |
| Patient was past the menopause | 45 | 155 | 200 | 69·4 |
| Not stated | 8 | 21 | 29 | 10·1 |
| Age at menopause : Under 40 | — | 6 | 6 | 2·9 |
| 40– | 1 | 8 | 9 | 4·4 |
| 44– | 10 | 25 | 35 | 17·2 |
| 48– | 21 | 71 | 92 | 45·1 |
| 52– | 13 | 42 | 55 | 27·0 |
| 56 and over | 1 | 6 | 7 | 3·4 |
| Interval between the menopause and onset of symptoms : 0–5 years | 15 | 40 | 55 | 27·0 |
| 5–10 ,, | 13 | 36 | 49 | 24·0 |
| Over 10 ,, | 18 | 82 | 100 | 49·0 |
| Total number who were past the menopause | 46 | 158 | 204 | 100·0 |

When compared with the corresponding figures for the cervix uteri it will be seen that 11·2 per cent more of those with cancer of the corpus uteri were past the menopause, owing to the later age of onset of the disease. Three patients had had an artificial menopause induced by means of radium for the treatment of menorrhagia and one by oöphorectomy. Lane-Claypon (1926) found that 29 of 509 control patients (5·7 per cent) had had non-cancerous conditions (Part I, p. 435).

| *Children and miscarriages.* | Single. | Married and widowed. | Total. |
|---|---|---|---|
| No children | 55 | 44 | 99 |
| One child | — | 59 | 59 |
| Two children | — | 31 | 31 |
| Three ,, | — | 29 | 29 |
| Four ,, | — | 16 | 16 |
| Five or more children | — | 23 | 23 |
| Not stated | 11 | 20 | 31 |
| No miscarriages | 53 | 141 | 194 |
| One miscarriage | — | 34 | 34 |
| Two miscarriages | — | 7 | 7 |
| Three or more miscarriages | — | 7 | 7 |
| Not stated | 13 | 33 | 46 |

The 99 nulliparous patients were divided into those who were totally barren and those who had had one or more miscarriages :

| | Single. | Married and widowed. | Total. |
|---|---|---|---|
| No children, no miscarriages | 55 | 37 | 92 |
| ,, but one or more miscarriages | — | 7 | 7 |

Of 257 women of known parity (288, less 31 unknown), 99 or 34·6 per cent were nulliparous, and 92 or 31·9 per cent were completely sterile. Lane-Claypon (1927) found from data in the literature that 29 per cent of 389 patients with cancer of the corpus were nulliparous. Dr. Stocks' comments on the figures have already been given in Part I, page 438.

*Interval between last pregnancy and first symptom of cancer in* 257 *patients of known parity.*

| | |
|---|---|
| No children or miscarriages | 92 |
| Pregnant at the time | — |
| Less than one year ago | 1 |
| 1–5 years ago | 3 |
| 5–10 ,, | 6 |
| 10–15 ,, | 14 |
| 15–20 ,, | 24 |
| 20–25 ,, | 15 |
| 25–30 ,, | 27 |
| 30–35 ,, | 20 |
| 35–40 ,, | 21 |
| Over 40 years ago | 15 |
| Not stated | 19 |

The maximum incidence is 15–30 years after the birth of the last child, which is five years later than in the case of cancer of the cervix.

| | No. | Per cent. |
|---|---|---|
| *History of puerperal lacerations.* | | |
| Any puerperal lacerations | 35 | 12·2 |
| No history | 178 | 61·8 |
| Not stated | 75 | 26·0 |
| *History of previous operations.* | | |
| Previous dilatation and curetting | 17 | 5·9 |
| Cervix operation | 9 | 3·1 |
| Myomectomy | 1 | 0·3 |
| Unilateral oöphorectomy | 3 | 1·0 |
| Bilateral ,, | 3 | 1·0 |
| Previous mastectomy for cancer | 1 | 0·3 |
| No history | 195 | 67·7 |
| Not stated | 59 | 20·5 |
| *History of pelvic infections.* | | |
| Any pelvic infection | 6 | 2·1 |
| No history | 174 | 60·4 |
| Not stated | 108 | 37·5 |
| *History of previous vaginal discharge.* | | |
| Present | 54 | 18·8 |
| Not present | 140 | 48·6 |
| Not stated | 94 | 32·6 |
| *History of previous fibromyoma.* | | |
| Present | 15 | 5·2 |
| Not present | 149 | 51·7 |
| Not stated | 124 | 43·1 |

*First Symptom.*

| | No. | Per cent. | |
|---|---|---|---|
| Irregular haemorrhage | 149 | 51·7 | 63·2 |
| Sudden profuse haemorrhage | 33 | 11·5 | |
| Discharge | 63 | 21·9 | |
| Pain | 20 | 6·9 | |
| Disorders of micturition | 6 | 2·1 | |
| Abdominal tumour or swelling | 5 | 1·7 | |
| Loss of weight | 3 | 1·0 | |
| Rectal symptoms | 1 | 0·3 | |
| Symptoms due to secondaries | 1 | 0·3 | |
| No pelvic symptoms, growth discovered during examination | 1 | 0·3 | |
| Not stated | 6 | 2·1 | |

In one patient the first symptom was haemoptysis; exploration revealed an extensive tumour diagnosed as a chorion-carcinoma following hydatiform mole five months before; the haemoptysis was probably due to a metastasis in the lung, but the patient was lost sight of.

*Interval from First Symptom to First Consultation.*

| | No. | Per cent. |
|---|---|---|
| One month and under | 55 | |
| 1–2 months | 24 | 33·7 |
| 2–3 ,, | 18 | |
| 3–4 ,, | 9 | 14·2 |
| 4–6 ,, | 32 | |
| 6–9 ,, | 28 | |
| 9–12 ,, | 28 | |
| 12–18 ,, | 24 | |
| 18–24 ,, | 17 | 39·6 |
| 24–48 ,, | 6 | |
| Over 48 months | 11 | |
| Not stated | 36 | 12·5 |

These figures show that two-thirds of the patients delayed more than three months, and nearly half of them more than six months before consulting a doctor

*Interval from First Consultation to First Vaginal Examination.*

| | No. | Per cent. |
|---|---|---|
| Done at first consultation | 221 | 76·7 |
| Within one month | 20 | 6·9 |
| 1–2 months later | 2 | 2·4 |
| 2–3 ,, ,, | 5 | |
| 3–4 ,, ,, | 1 | |
| 4–5 ,, ,, | 1 | 1·0 |
| 5–6 ,, ,, | 1 | |
| 6–8 ,, ,, | — | |
| 8–10 ,, ,, | 1 | 0·3 |
| 10–12 ,, ,, | 1 | 0·3 |
| Over 12 months later | 1 | 0·3 |
| Not stated | 34 | 11·8 |

*Advice and Treatment before Admission to Hospital.*

| | No. |
|---|---|
| No doctor consulted prior to coming to hospital | 29 |
| Referred to hospital without delay | 191 |
| ,, ,, but delayed going there | 11 |
| Treated symptomatically for periods up to 3 months before reference | 10 |
| ,, ,, ,, over 3 months before reference | 17 |
| Reassured or kept under observation | 6 |
| Not stated | 24 |

The figures in the above two paragraphs show that in 83·6 per cent of the patients a vaginal examination was made at, or within one month of the first consultation, and that of the 259 patients who consulted a doctor 73·7 per cent were referred to hospital at once. 6·6 per cent were kept under symptomatic treatment for more than three months, but this figure includes patients who were unsuitable for treatment, either by reason of poor general condition or the disease being too advanced. In six instances (2·3 per cent) the patient was told that the symptoms were due to the menopause and that she need not worry about them.

*Symptoms on Admission to Hospital.*

These are shown in order of frequency of occurrence, though most patients complained of more than one symptom.

| | No. | Per cent. |
|---|---|---|
| Haemorrhage | 243 | 84·4 |
| Discharge | 177 | 61·5 |
| Pain | 112 | 38·9 |
| Abdominal tumour or swelling | 67 | 23·3 |
| Loss of weight | 40 | 13·9 |
| Disorders of micturition | 36 | 12·5 |
| Anaemia | 11 | 3·8 |
| Rectal symptoms | 1 | 0·3 |
| Symptoms due to secondaries | 3 | 1·0 |

*Findings on Examination.*

| *Size of uterus.* | No. | Per cent. |
|---|---|---|
| Enlarged | 185 | 64·2 |
| Not enlarged | 66 | 22·9 |
| Not stated | 37 | 12·8 |
| *Mobility of uterus.* | | |
| Freely mobile | 194 | 67·4 |
| Mobility limited | 24 | 8·3 |
| Fixed | 42 | 14·6 |
| Not stated | 28 | 9·7 |
| *Involvement of cervix.* | | |
| Involved | 22 | 7·6 |
| Not involved | 233 | 80·9 |
| Not stated | 33 | 11·5 |
| *Fibromyoma present simultaneously.* | | |
| Present | 30 | 10·4 |
| Not present | 216 | 75·0 |
| Not stated | 42 | 14·6 |

| *Type of fibromyoma.* | No. |
|---|---|
| Not specified | 15 |
| Subserous | 4 |
| Submucous | 2 |
| Massive | 9 |

Norris and Vogt (1924) found that 20·8 per cent of cases of fundus carcinoma were associated with myoma, and that the symptoms of myoma usually obscured those of carcinoma. The relationship between sarcoma and myoma in this series of cases is dealt with on page 469.

*Extension to Neighbouring Organs.*

| | No. | Per cent. |
|---|---|---|
| *Vagina.* | | |
| Involved by extension | 12 | 4·2 |
| ,, isolated metastatic nodules | 6 | 2·1 |
| Not involved | 242 | 84·0 |
| Not stated | 28 | 9·7 |
| *Broad ligament.* | | |
| Involved | 39 | 13·5 |
| Not involved | 208 | 72·2 |
| Not stated | 41 | 14·2 |
| *Bladder.* | | |
| Involved | 6 } | 2·1 |
| Involvement confirmed by cystoscopy | 2 } | |
| Not involved | 239 | 83·0 |
| Not stated | 43 | 14·9 |
| *Rectum.* | | |
| Involved | 11 | 3·8 |
| Not involved | 247 | 85·8 |
| Not stated | 30 | 10·4 |

*Local Metastases.*

| | No. | Per cent. |
|---|---|---|
| *Pelvic lymph nodes.* | | |
| Pelvic masses present | 44 | 15·3 |
| ,, ,, not present | 219 | 76·0 |
| Not stated | 25 | 8·7 |
| *Inguinal lymph nodes.* | | |
| Involved | 4 | 1·4 |
| Not involved | 221 | 76·7 |
| Not stated | 63 | 21·9 |

*Remote Metastases.*

| | No. | Per cent. |
|---|---|---|
| None found on clinical examination | 243 } | 84·7 |
| ,, ,, after radiological examination | 1 } | |
| Metastases present | 20 | 6·9 |
| Not stated | 24 | 8·3 |

*Sites of Metastases in 20 Patients (multiple in 2).*

| | No. |
|---|---|
| Abdominal lymph nodes and peritoneum | 11 |
| Liver and abdominal organs | 6 |
| Lungs and pleurae | 2 |
| Bones of pelvis | 1 |
| Skin and subcutaneous tissues | 1 |
| Lymph nodes other than regional | 1 |

*Wassermann Reaction.*

The Wassermann reaction was tested in 11 patients and was found to be negative in all.

*General Condition. Other Co-existing Diseases.*

| | No. | Per cent. |
|---|---|---|
| Good; no weight loss | 130 } | 54·9 |
| Obese; but otherwise good | 28 } | |
| Fair; moderate weight loss (up to 2 st.) | 62 | 21·5 |
| Poor; considerable weight loss (over 2 st.) | 37 | 12·8 |
| Emaciated | 5 | 1·7 |
| Moribund | 5 | 1·7 |
| Not stated | 21 | 7·3 |

Four patients were found to be suffering from diabetes, and 10 from cardiovascular disease.

*Clinical Stages.*

The cases were arranged in five groups according to the clinical findings given on pages 459, and 460.

| | No. | Per cent. |
|---|---|---|
| Group I: Disease limited to corpus uteri | 170 | 59·0 |
| ,, II: ,, has spread into parametrial tissues, tubes or cervix | 37 | 12·8 |
| ,, III: ,, Disease had involved regional nodes, vagina, bladder or rectum | 45 | 15·6 |
| ,, IV: ,, Remote metastases outside pelvis present | 21 | 7·3 |
| Not grouped for lack of data | 15 | 5·2 |

In the cases of patients who underwent operation, the operation and pathological findings, where available, were used for correcting the groups into stages. Patients not operated on were placed in the appropriate stage according to the clinical evidence of extension of the growth and the presence of metastases. The final staging is shown below :

| | No. | | Per cent. |
|---|---|---|---|
| *Stage I :* Disease limited to corpus uteri. | | | |
| Confined to corpus uteri clinically . | 128 | | |
| ,, endometrium histologically | 20 | | |
| Invading myometrium . . . | 41 | | |
| | 189 | . | 65·6 |
| *Stage II :* Disease has spread outside corpus uteri. | | | |
| Growth has involved the cervix uteri . | 10 | | |
| ,, ,, peritoneum covering the uterus . | 6 | | |
| ,, ,, parametrial tissues | 20 | | |
| ,, ,, tubes or ovaries | 8 | | |
| | 44 | . | 15·3 |
| *Stage III :* Disease has involved regional lymph nodes or adjacent organs. | | | |
| Regional lymph nodes involved . . | 9 | | |
| Vagina involved . . . . . | 14 | | |
| Bladder or rectum involved . . | 5 | | |
| | 28 | . | 9·7 |
| *Stage IV :* Remote metastases present. | | | |
| Remote metastases outside the pelvis present . . . . . . | 27 | . | 9·4 |

The differences between the clinical and final staging will be seen from the following table, which shows that more patients are still in Stages I and II than appear to be so on the clinical findings.

| | Clinical stages. | Final stages. | Difference. |
|---|---|---|---|
| Stage I | 170 | 189 | +19 |
| ,, II | 37 | 44 | +7 |
| ,, III | 45 | 28 | —17 |
| ,, IV | 21 | 27 | +6 |
| Not staged | 15 | — | —15 |

*Methods of Treatment and 5-year Results.*

All deaths from any cause within one month of an operation, whether radical or palliative, were counted as "operation fatalities." In the case of radiotherapeutic treatment those patients whose death appeared to have been accelerated by the effects of radiotherapy have been classified as " died from effects of treatment," regardless of the time which had elapsed since treatment was completed.

| *Surgical and combined methods.* | No. | Operation fatalities. | Died from effects of treatment. | Survived 5 years. | Died with cancer. | Died without cancer. | Not traced. |
|---|---|---|---|---|---|---|---|
| Panhysterectomy with excision of tubes and ovaries | 74 | 4 | — | 42 | 17 | 1 | 10 |
| Panhysterectomy with excision of tubes and ovaries preceded by radium or by radium and X-rays | 12 | 1 | — | 9 | 2 | — | — |
| Panhysterectomy with excision of tubes and ovaries followed by H.V. X-rays | 22 | — | — | 12 | 6 | 1 | 3 |
| Panhysterectomy | 22 | 5 | — | 8 | 7 | 1 | 1 |
| Panhysterectomy preceded by radium or by radium and H.V. X-rays | 5 | — | — | 3 | 1 | — | 1 |
| Panhysterectomy followed by H.V. X-rays | 9 | — | — | 7 | 2 | — | — |
| Supravaginal hysterectomy | 14 | — | — | 5 | 7 | — | 2 |
| Supravaginal hysterectomy followed by H.V. X-rays | 7 | — | — | 5 | 2 | — | — |
| Supravaginal hysterecomy followed by radium and H.V. X-rays | 1 | — | — | — | 1 | — | — |
| Vaginal hysterectomy | 2 | — | — | 2 | — | — | — |
| Exploratory laparotomy | 9 | 1 | — | — | 7 | — | 1 |
| | 177 | 11 | — | 93 | 52 | 3 | 18 |
| *Radiotherapy alone.* | | | | | | | |
| Radium alone | 50 | 2 | 1 | 22 | 20 | 5 | — |
| Radium with H.V. X-rays | 24 | — | 2 | 10 | 12 | — | — |
| H.V. X-rays alone | 5 | — | — | 2 | 3 | — | — |
| H.V. X-rays following exploratory laparotomy | 7 | — | — | — | 7 | — | — |
| | 86 | 2 | 3 | 34 | 42 | 5 | — |
| *Not treated by surgery or radiotherapy.* | 25 | — | — | — | 25 | — | — |
| Total of all cases | 288 | 13 | 3 | 127 | 119 | 8 | 18 |

There were 11 operation fatalities in 177 cases, or 6·2 per cent. There were 2 fatalities due to perforation of the infiltrated uterine wall, and one death from a pelvic abscess following radium treatment and 2 deaths from pelvic peritonitis following radium and X-ray treatment.

### *Analysis of Methods of Treatment by Stages.*

| | Stage I. | Stage II. | Stage III. | Stage IV. | Total. | Per cent. of total. |
|---|---|---|---|---|---|---|
| Number in each stage | 189. | 44. | 28. | 27. | 288. | 100·0. |
| Radical surgical methods | 86 | 16 | 8 | 2 | 112 | 38·9 |
| „ combined „ | 41 | 11 | 3 | 1 | 56 | 19·4 |
| Exploratory laparotomy alone or with radiotherapy | 1 | 6 | 2 | 7 | 16 | 5·6 |
| Radiotherapeutic methods | 58 | 9 | 9 | 3 | 79 | 27·4 |
| Not treated by surgery or radiotherapy | 3 | 2 | 6 | 14 | 25 | 8·7 |
| Percentage treated by radical surgical or combined methods | 67·2 | 61·4 | 39·3 | 11·1 | 58·3 | — |

### *Duration of Symptoms at Time of Commencing Treatment.*

| | No. | Survived. | Per cent. |
|---|---|---|---|
| 1 month and under | 10 | 6 | |
| 1–3 months | 45 | 19 | 49·5 |
| 3–6 „ | 44 | 24 | |
| 6–12 „ | 72 | 33 | |
| Over 12 months | 84 | 43 | 48·7 |
| Not known | 8 | 2 | |
| Total | 263 | 127 | |

The estimates of the duration of symptoms, like those given on page 458, are based on the patients' statements. It will be seen that there is no significant difference between the survival rates of those who came under treatment within the first six months and those in whose cases the delay was longer. The former constitute 37·6 per cent of the treated patients and the latter 59·3 per cent.

*Five-year Follow-up Results for all Cases.*

| | First year. | Second year. | Third year. | Fourth year. | Fifth year. | Totals. | | |
|---|---|---|---|---|---|---|---|---|
| Operation fatalities | 12 | 1 | — | — | — | 13 | | |
| Died from effects of treatment | 3 | — | — | — | — | 3 | 143 | |
| „ with cancer | 73 | 30 | 4 | 7 | 4 | 118 | | |
| „ without cancer | 1 | 3 | 1 | 1 | 3 | 9 | | |
| Alive and well | — | — | — | — | 82 | | | |
| „ with cancer | — | — | — | — | 9 | | 127 | 288 |
| „ state unknown | — | — | — | — | 36 | | | |
| Untraced | — | — | — | — | 18 | | 18 | |
| Total died each year | 89 | 34 | 5 | 8 | 7 | | | |
| Percentage of all cases | 30·9 | 11·8 | 1·7 | 2·8 | 2·4 | | | |

The five-year survival rate for all cases was 44·1 per cent. Two of the 127 survivors are known to have died of cancer early in the sixth and ninth year respectively; six patients died of other causes without evidence of recurrence, 3 in the sixth year, 2 in the seventh, and 1 in the eighth year.

*Analysis of 5-year Results by Methods of Treatment and Stages.*

*Surgical or combined methods.*

| | Stage I. | Stage II. | Stage III. | Stage IV. | Total. |
|---|---|---|---|---|---|
| Panhysterectomy with excision of tubes and ovaries | 57 | 11 | 6 | — | 74 |
| Known survivors | 36 | 4 | 2 | — | 42 |
| Per cent survived | 63·2 | 36·4 | 33·3 | — | 56·8 |
| Per cent of traced cases | 73·5 | 44·4 | 33·3 | — | 65·6 |
| Panhysterectomy with excision of tubes and ovaries preceded by radiotherapy | 8 | 3 | 1 | — | 12 |
| Known survivors | 6 | 2 | 1 | — | 9 |
| Per cent survived | 75·0 | 66·6 | 100·0 | — | 75·0 |
| Panhysterectomy with excision of tubes and ovaries followed by H.V. X-rays | 15 | 5 | 2 | — | 22 |
| Known survivors | 8 | 3 | 1 | — | 12 |
| Per cent survived | 53·3 | 60·0 | 50·0 | — | 54·5 |
| Per cent of traced cases | 57·2 | 100·0 | 50·0 | — | 63·2 |
| Panhysterectomy alone | 15 | 3 | 2 | 2 | 22 |
| Known survivors | 7 | 1 | — | — | 8 |
| Per cent survived | 46·6 | 33·3 | 0·0 | 0·0 | 36·6 |
| Panhysterectomy preceded by radiotherapy | 4 | 1 | — | — | 5 |
| Known survivors | 3 | — | — | — | 3 |
| Per cent survived | 75·0 | 0·0 | — | — | 60·0 |
| Per cent of traced cases | 100·0 | 0·0 | — | — | 75·0 |
| Panhysterectomy followed by H.V. X-rays | 9 | — | — | — | 9 |
| Known survivors | 7 | — | — | — | 7 |
| Per cent survived | 77·7 | — | — | — | 77·7 |
| Supravaginal hysterectomy alone | 12 | 2 | — | — | 14 |
| Known survivors | 3 | 2 | — | — | 5 |
| Per cent survived | 25·0 | 100·0 | — | — | 35·7 |
| Per cent of traced cases | 30·0 | 100·0 | — | — | 41·7 |

| *Surgical or combined methods*—cont. | Stage I. | Stage II. | Stage III. | Stage IV. | Total. |
|---|---|---|---|---|---|
| Supravaginal hysterectomy followed by H.V. X-rays | 5 | 2 | — | 1 | 8 |
| Known survivors | 3 | 2 | — | — | 5 |
| Per cent survived | 60·0 | 100·0 | — | 0·0 | 62·5 |
| Total of radical surgical and combined methods | 127 | 27 | 11 | 3 | 168 |
| Known survivors | 75 | 14 | 4 | — | 93 |
| Per cent survived | 59·1 | 51·9 | 36·4 | 0·0 | 55·4 |
| Per cent of traced cases | 65·2 | 60·9 | 36·4 | 0·0 | 61·6 |
| *Radiotherapy alone.* | | | | | |
| Radium alone | 40 | 5 | 3 | 2 | 50 |
| Known survivors | 22 | — | — | — | 22 |
| Per cent survived | 55·0 | 0·0 | 0·0 | 0·0 | 44·0 |
| Combinations of radium and X-rays | 18 | 3 | 3 | — | 24 |
| Known survivors | 8 | 1 | 1 | — | 10 |
| Per cent survived | 44·4 | 33·3 | 33·3 | — | 41·7 |
| H.V. X-rays alone | — | 1 | 3 | 1 | 5 |
| Known survivors | — | — | 2 | — | 2 |
| Per cent survived | — | 0·0 | 66·6 | 0·0 | 40·0 |
| Total of radiotherapeutic methods | 58 | 9 | 9 | 3 | 79 |
| Known survivors | 30 | 1 | 3 | — | 34 |
| Per cent survived | 51·7 | 11·1 | 33·3 | 0·0 | 43·0 |
| *Total of all cases treated and not treated.* | | | | | |
| Total number of cases | 189 | 44 | 28 | 27 | 288 |
| Known survivors | 105 | 15 | 7 | — | 127 |
| Per cent survived | 55·5 | 34·1 | 25·0 | 0·0 | 44·1 |
| Per cent of traced cases | 59·3 | 38·5 | 25·0 | 0·0 | 47·0 |

*Analysis of 5-year Results by Ages in Relation to Methods of Treatment in Stage 1 Cases only.*

| | Panhysterectomy with excision of tubes and ovaries, with or without radiotherapy. | | | Radium alone. | | |
|---|---|---|---|---|---|---|
| Age group. | Total No. | Known survivors. | Per cent. | Total No. | Known survivors. | Per cent. |
| 25– | 1 | 1 | — | — | — | — |
| 35– | 4 | 4 | — | — | — | — |
| 45– | 32 | 22 | — | 4 | 4 | — |
| 55– | 33 | 18 | — | 20 | 13 | — |
| 65– | 10 | 5 | — | 13 | 4 | — |
| 75– | — | — | — | 3 | 1 | — |
| | 80 | 50 | 62·5 | 40 | 22 | 55·0 |
| | $\chi^2 = 5{\cdot}08$ | $P < 0{\cdot}30 > 0{\cdot}20$ | | $\chi^2 = 7{\cdot}72$ | $P < 0{\cdot}20 > 0{\cdot}10$ | |

In both the above examples the figures for survival by age groups are not statistically significant, as the value of *P* shows that this distribution might occur by chance 1 in 5 times and 1 in 10 times respectively.

*Estimation of Survival after Treatment.*

| | | Stage I. | Stage II. | Stage III. |
|---|---|---|---|---|
| **Panhysterectomy with excision of tubes and ovaries—74 cases** | | | | |
| Number of cases of known duration | | 53 | 8 | 5 |
| Mean number of months lived in 5 years from onset | Maximum possible | 58·64 | 58·50 | 60·00 |
| | Expected | 56·09 | 55·47 | 56·27 |
| | Actual | 49·96 | 41·37 | 30·20 |
| Per cent of Expected | | 89·07 | 74·58 | 53·67 |
| **Panhysterectomy with excision of tubes and ovaries preceded by radiotherapy—12 cases.** | | | | |
| Number of cases of known duration | | 8 | 3 | 1 |
| Mean number of months lived in 5 years from onset | Maximum possible | 60·00 | 60·00 | — |
| | Expected | 57·67 | 56·23 | — |
| | Actual | 49·50 | 60·00 | — |
| Per cent of Expected | | 85·83 | 106·70 | — |
| **Panhysterectomy with excision of tubes and ovaries followed by radiotherapy—22 cases.** | | | | |
| Number of cases of known duration | | 15 | 4 | 2 |
| Mean number of months lived in 5 years from onset | Maximum possible | 59·20 | 57·00 | — |
| | Expected | 56·76 | 55·11 | — |
| | Actual | 45·07 | 57·00 | — |
| Per cent of Expected | | 79·40 | 103·43 | — |
| **Panhysterectomy alone—22 cases.** | | | | |
| Number of cases of known duration | | 15 | 3 | 2 |
| Mean number of months lived in 5 years from onset | Maximum possible | 60·00 | 60·00 | — |
| | Expected | 57·46 | 58·16 | — |
| | Actual | 46·20 | 23·66 | — |
| Per cent of Expected | | 80·40 | 40·68 | — |
| **Panhysterectomy alone preceded by radiotherapy—5 cases.** | | | | |
| Number of cases of known duration | | 3 | 2 | — |
| Mean number of months lived in 5 years from onset | Maximum possible | 60·00 | — | — |
| | Expected | 56·43 | — | — |
| | Actual | 60·00 | — | — |
| Per cent of Expected | | 106·43 | — | — |

| | | Stage I. | Stage II. | Stage III. |
|---|---|---|---|---|
| **Panhysterectomy alone followed by radiotherapy—9 cases.** | | | | |
| Number of cases of known duration | | 9 | — | — |
| Mean number of months lived in 5 years from onset | Maximum possible | 60·00 | — | — |
| | Expected | 58·56 | — | — |
| | Actual | 60·00 | — | — |
| Per cent of Expected | | 102·46 | — | — |
| **Supravaginal hysterectomy alone—14 cases.** | | | | |
| Number of cases of known duration | | 10 | 2 | — |
| Mean number of months lived in 5 years from onset | Maximum possible | 56·40 | — | — |
| | Expected | 53·96 | — | — |
| | Actual | 38·10 | — | — |
| Per cent of Expected | | 79·87 | — | — |
| **Supravaginal hysterectomy followed by radiotherapy—8 cases.** | | | | |
| Number of cases of known duration | | 4 | 2 | — |
| Mean number of months lived in 5 years from onset | Maximum possible | 60·00 | — | — |
| | Expected | 58·44 | — | — |
| | Actual | 58·50 | — | — |
| Per cent of Expected | | 100·10 | — | — |
| **Radium alone—50 cases.** | | | | |
| Number of cases of known duration | | 39 | 5 | 3 |
| Mean number of months lived in 5 years from onset | Maximum possible | 60·00 | 60·00 | — |
| | Expected | 54·90 | 51·80 | — |
| | Actual | 46·72 | 39·40 | — |
| Per cent of Expected | | 85·10 | 76·07 | — |
| **Combinations of radium and X-rays—24 cases.** | | | | |
| Number of cases of known duration | | 18 | 3 | 3 |
| Mean number of months lived in 5 years from onset | Maximum possible | 60·00 | — | — |
| | Expected | 54·18 | — | — |
| | Actual | 46·11 | — | — |
| Per cent of Expected | | 85·11 | — | — |

| Not treated by surgery or by radiotherapy—25 cases. | | All stages. |
|---|---|---|
| Number of cases of known duration | | 17 |
| Mean number of months lived in 5 years from onset | Maximum possible | 60·00 |
| | Expected | 54·40 |
| | Actual | 17·41 |
| Per cent of Expected | | 32·00 |

These figures show that in Stage I hysterectomy with excision of tubes and ovaries gave the best results, which were not improved by the use of radiotherapy either before or after operation. The 5-year survival rate and the expectation of life after panhysterectomy alone in Stage I were both higher when operation was supplemented by radiotherapy, but the number of cases so treated was small. Supravaginal hysterectomy alone in Stage I gave a very poor survival rate, but a fair expectation of life which was increased by the use of radiotherapy in addition. Treatment by radium alone in Stage I gave a 5-year survival rate of 55 per cent as against 73·5 per cent after radical operation, but when estimated by actuarial methods the former showed a 5-year expectation of life of 85·1 per cent as against 89·07 per cent for the latter. The numbers of patients in Stages II and III who were treated were too small for the differences in 5-year survival rates or expectation of life between different methods of treatment to be significant.

*Biopsy.*

| | No. | | Per cent. |
|---|---|---|---|
| Curettage and biopsy done before treatment | 89 | 185 | 64·2 |
| ,, ,, ,, at commencement of treatment | 71 | | |
| ,, ,, ,, during radiation treatment | — | | |
| ,, ,, ,, but time not stated | 25 | | |
| ,, ,, not done | 103 | | 35·8 |

*Pathological Report.*

| | No. | Per cent. |
|---|---|---|
| No histological examination before or after death | 33 | 11·5 |
| Histological examination done before or after death | 255 | 88·5 |
| The specimen examined was non-malignant | 1 | — |
| Result of histological examination was doubtful | 4 | — |
| Adenocarcinoma | 173 | — |
| ,, papillary | 26 | — |
| Spheroidal cell carcinoma | 4 | — |
| Squamous cell carcinoma, keratinizing | 6 | — |
| ,, ,, ,, non-keratinizing | 1 | — |
| ,, ,, ,, undifferentiated | 3 | — |
| Chorion-carcinoma | 2 | — |
| Carcinoma, type unspecified | 17 | — |
| Sarcoma | 14 | — |
| Spindle cell sarcoma | 3 | — |
| Leio-myosarcoma | 1 | — |

*Degree of invasion found on histological examination.*

| | No. |
|---|---|
| Growth confined to endo metrium of corpus uteri | 16 |
| ,, had invaded the muscle ,, ,, | 58 |
| ,, ,, spread to peritoneum | 8 |
| ,, ,, ,, cer vix | 10 |
| ,, ,, ,, tub es or ovaries | 12 |
| ,, ,, ,, pelvic lymph nodes | 8 |
| No pathological report on degree of extension | 138 |

The two patients with chorion-carcinoma were aged 25 and 27 years respectively. One had borne no children but had had a miscarriage two years before admission ; the other had borne a child four and a half months previously. The latter patient is still alive, the former died with metastases soon after admission.

*Relationship between sarcoma and fibromyoma.*

Amongst the 250 tumours which were microscopically examined there were 14 round cell or pleomorphic sarcomata, 3 spindle cell sarcomata, and one leiomyosarcoma. The incidence of fibromyoma amongst these 18 patients was as follows :

| | No. |
|---|---|
| Sarcoma arose in a degenerating fibromyoma . . . | 9 |
| There was a history of previous fibromyoma . . . | 2 |
| ,, no ,, ,, ,, and none was present . | 5 |
| Not stated . . . . . . . . . . | 2 |

*Basis of diagnosis in* 288 *cases.*

*Clinically malignant :*

| | No. | | Per cent. |
|---|---|---|---|
| Proved malignant by histological examination and/or autopsy . . . . | 251 | . | 87·2 |
| ,, ,, appearance or metastases of recurrence . . . . | 13 | . | 4·5 |
| Diagnosis based on clinical evidence only . . | 12 | . | 4·2 |
| *Clinically benign :* | | | |
| Proved malignant by histological examination . . | 12 | . | 4·2 |

*Other Primary Growths.*

*Previous primary tumours*—4 patients.

1. Age 57. Breast amputated three years previously for cancer; no sign of recurrence. Admitted for columnar adenocarcinoma of uterus; treated by panhysterectomy with salpingo-oophorectomy. Died from abdominal metastases in the second year.

2. Age 78. Treated by X-rays two and a half years previously for cancer of the left breast. Diagnosed by biopsy of an axillary node ; no sign of recurrence. Admitted for columnar cell carcinoma of uterus ; treated by radium. Died from cancer in the third year.

3. Age 67. Breast amputated three years previously ; no sign of recurrence. Admitted for adenocarcinoma of uterus, treated by radium and X-rays. Died three months later from metastases in the liver and abdominal organs.

4. Age 59. Radical mastectomy for spheroidal cell carcinoma of breast nine years previously, no sign of recurrence. Admitted for cancer of uterus (no histology) ; treated by radium ; recurrence one year later and death from generalized metastases.

*Simultaneous primary growths*—6 patients.

5. Age 68. Admitted for columnar cell carcinoma of uterus; treated by panhysterectomy with salpingo-oophorectomy. A tumour in the left breast was found on examination, diagnosed as cancer, and treated by radical mastectomy. The patient died in the first year from pelvic metastases.

6. Age 63. Admitted for adenocarcinoma of uterus co-existing with a fibromyoma which had been present for many years. Treated by panhysterectomy with salpingo-oophorectomy. A tumour in the left breast was found on examination, diagnosed as cancer, and treated by X-rays. The patient died in the fourth year from metastases in the lungs, probably arising from the breast tumour.

7. Age 78. Admitted for cancer of uterus of about three years' standing. Found to have a tumour in the left breast, diagnosed as cancer. Died soon after admission.

8. Age 60. Admitted for adenocarcinoma of uterus; treated by panhysterectomy with salpingo-oophorectomy. Found to have a rodent ulcer of face of five years' standing; no note as to treatment. Died in the third year from recurrence of carcinoma in the pelvis.

9. Age 61. Admitted for columnar cell adenocarcinoma of uterus; treated by panhysterectomy. Found to have a rodent ulcer of the nose, which was treated by X-rays. Alive with no sign of recurrence of either growth after five years.

10. Age 60. Admitted for adenocarcinoma of uterus; treated by panhysterectomy and salpingo-oophorectomy. Metastases found in right ovary. Found to have a growth in right breast, diagnosed as cancer, for which treatment was refused. Not traced.

*Subsequent primary growths*—one patient.

11. Age 66. Admitted for columnar cell carcinoma of uterus of four months standing; treated by radium followed by panhysterectomy. One month later a growth in the right breast was found on biopsy to be a highly malignant spheroidal cell carcinoma. No treatment for the breast growth. Died in the first year from the growth in the uterus.

*Cause of Death in* 143 *Patients.*

| | No. |
|---|---|
| Cachexia | 97 |
| Cardio-vascular disease | 8 |
| Pulmonary complications | 8 |
| Uraemia | 5 |
| Peritonitis | 5 |
| Intestinal obstruction | 3 |
| Haemorrhage | 3 |
| Pulmonary embolism | 3 |
| Sepsis | 2 |
| Surgical shock | 1 |
| Cerebral embolism | 1 |
| Intercurrent disease or unknown cause | 7 |

*Autopsy.*

| | No. | Per cent. |
|---|---|---|
| No autopsy | 108 | 75·5 |
| Autopsy done | 35 | 24·5 |

*Relevant autopsy findings (multiple in some cases).*

| | No. |
|---|---|
| Local growth only | 5 |
| Extension to neighbouring parts | 15 |
| Metastases in pelvic lymph nodes | 6 |
| ,, abdominal lymph nodes | 8 |
| ,, liver and abdominal organs | 11 |
| ,, lungs and pleurae | 11 |
| ,, brain | 1 |
| ,, skeletal system | 1 |
| Pulmonary complications | 8 |
| Abdominal ,, | 5 |
| No growth found (operated cases) | 7 |

RECURRENT CASES.

There were 33 recurrent cases, of which 21 followed panhysterectomy (with salpingo-oophorectomy in 4 cases) for cancer, 2 followed hysterectomy for fibroids, and 10 followed radium treatment for cancer. The relation of the type of recurrence to the method of treatment of the primary and the mean intervals of freedom are shown below :

| *Following panhysterectomy for cancer*—21 cases. | No. | Mean interval of freedom in months. |
|---|---|---|
| Local recurrence only | 4 | 16·7 |
| Recurrence deep in pelvis | 9 | 19·7 |
| ,, both local and in pelvis | 2 | 3·5 |
| Distant metastases | 6 | 16·2 |

The shortest interval was two months and the longest four years.

| *Following hysterectomy for fibroids*—2 cases. | | |
|---|---|---|
| Recurrence deep in pelvis | 2 | 125 |

In both these cases the diagnosis of malignancy was made on the histological examination. One operation was a total hysterectomy, followed by 15 years of freedom from recurrence, and one was a subtotal hysterectomy for a fibromyoma, reported histologically to be suggestive of sarcoma, and followed by freedom for nearly 6 years.

| *Following radium treatment for cancer*—10 cases. | No. | Mean interval of freedom in months. |
|---|---|---|
| Recurrence deep in pelvis | 6 | 13·0 |
| ,, both local and in pelvis | 1 | 18·0 |
| Distant metastases | 3 | 25·7 |

The shortest interval was five months, and the longest five years.

There were histological reports, either on the primary or on the recurrence, in 17 cases: Three sarcomata, 2 undifferentiated carcinomata, and 12 adeno-carcinomata.

Fifteen patients received further treatment: by X-rays in 9 cases, radium in 4, radium with X-rays in one, and by hysterectomy followed by X-rays in one patient in whom the primary had been treated by radium. The latter patient was the only one of the 33 who survived five years.

### SUMMARY.

1. A statistical analysis of 321 cases of cancer of the corpus uteri. 288 of these were primary cases.

2. 22·9 per cent of the 288 primary cases were single women and 77·1 per cent were married or widowed. The mean age of the 66 single women was 58·3 years, and that of the 222 married and widowed 58·6 years.

3. Analysis of the ratios of married and widowed to single women by age groups (cervix uteri Part I, page 434) led to the conclusion that liability to cancer of the corpus uteri is somewhat less among married and widowed than amongst single women between the ages of 35 and 65.

4. There were 99 nulliparous patients in all, and amongst 202 married and widowed patients of known parity there were 44 nulliparae against 33 expected (calculated from the percentages of childless married and widowed women in each age group registered as dying from all causes in England and Wales in 1939), indicating a greater liability to this form of cancer amongst nulliparous than amongst parous married women.

5. 5·2 per cent of the patients gave a history of having suffered from uterine fibromyomata, and in 10·4 per cent the patient was found to be suffering from both conditions simultaneously.

6. Irregular haemorrhage was the first symptom in 63·2 per cent of the patients, vaginal discharge in 21·9 per cent.

7. Only 33·7 per cent of the patients consulted a doctor within three months of noticing the first symptom, 14·2 per cent within the next three months, and in 39·6 per cent the symptoms were of more than six months' standing before advice was sought; in 17 cases the delay was more than two years.

8. In 76·7 per cent a vaginal examination was made at the first consultation, and in a further 6·9 per cent it was done within one month of the first consultation. In only 4·3 per cent was the examination delayed longer than one month.

9. 73·7 per cent of those who consulted a doctor were referred to hospital forthwith, 3·8 per cent were treated symptomatically for periods up to three months before reference, and 6·6 per cent for longer periods, but this figure included patients who were unsuitable for active treatment by reason of poor general condition or of the disease being too advanced. In 2·3 per cent the patient was told that the symptoms were due to the menopause.

10. On admission to hospital it was found that in 65·6 per cent of the patients the disease was still confined to the uterus, in 15·3 per cent it had spread to the parametrial tissues or to the cervix or Fallopian tubes, in 9·7 per cent the regional nodes, bladder or rectum were involved, and in 9·4 per cent there were metastases outside the pelvis.

11. Seventy-four patients were treated by panhysterectomy with bilateral salpingo-oophorectomy, with an operative mortality of 5·4 per cent and a five-year survival rate of 73·5 per cent of traced cases in Stage I, 44·4 per cent in Stage II, and 33·3 per cent in Stage III. Estimated actuarially, the five-year expectation of life was 89·07 per cent of normal expectation for Stage I, 74·5 per cent for Stage II, and 53·6 per cent for Stage III.

12. In 12 patients panhysterectomy with bilateral salpingo-oöphorectomy was preceded by radium treatment, with a five-year survival rate of 75·0 per cent, and in 22 it was followed by radiotherapy, with a five-year survival rate of 63·2 per cent of traced cases. The five-year survival rates showed that there was definite improvement in the results in patients who were classed as Stage II and III, but no improvement in those in Stage I. The numbers so treated were too small for the results to be statistically significant.

13. Twenty-two patients were treated by panhysterectomy alone, with an operative mortality of 22·7 per cent and a five-year survival rate of 46·6 per cent in Stage I. When the operation was preceded by radium treatment, 75·0 per cent of four patients survived five years, and when it was followed by radio therapy, 77·7 per cent of nine patients survived five years. These numbers also were too small for the results to be statistically significant.

14. Eighty-six patients were treated by radiotherapy alone, with five deaths due to effects of treatment (5·8 per cent) and a five-year survival rate of 43·0 per cent.

15. Twenty-five patients were not treated either by surgery or radiotherapy; all are dead after an average duration of life of 17·4 months.

16. Biopsy was done in 64·2 per cent, and there were histological reports for 88·5 per cent of the patients.

17. There were 21 patients with recurrent growths following panhysterectomy for cancer, two following hysterectomy for growths diagnosed clinically as fibroids, and ten following radium treatment for cancer. Fifteen of these patients received further treatment, 14 by radiotherapy, and one, whose primary growth had been treated by radium, had hysterectomy performed ; the latter patient was the only one who survived five years.

The Committee wish to thank Dr. Malcolm Donaldson, Dr. Margaret Tod and Dr. Mary Gilmour, who constituted the Sub-Committee which revised and edited this report, for their valuable advice and help.

### REFERENCES.

HARNETT, W. L.—(1948) *Brit. J. Cancer*, **2**, 128.
LANE-CLAYPON, J. E.—(1926) *Ministry of Health Reports*, **32**.—(1927) *Ibid.*, **40**.
*Idem* AND MCCULLAGH, W. McK. H.—(1927) *Ibid.*, **47**.
NORRIS, C. C., AND VOGT, M. E.—(1924) *Amer. J. Obstet. Gynaec.*, **7**, 550.
STOCKS, P., AND KARN, N. M.—(1933) *Ann. Eugen., Camb.*, **5**, 237.